# Insights Into the *Mondia Whitei* Microbiome Across Geographic Regions in Eastern Africa

**DOI:** 10.1111/1758-2229.70200

**Published:** 2025-09-27

**Authors:** Expedito Olimi, Regina Wuggenig, Carolina Lobato, Samuel Bickel, Peter Kusstatscher, Wisnu Adi Wicaksono, Angelika Battisti, Danny Coyne, John Adriko, Tomislav Cernava, Gabriele Berg

**Affiliations:** ^1^ Institute of Environmental Biotechnology Graz University of Technology Graz Austria; ^2^ School of Biological Sciences, Faculty of Environmental and Life Sciences University of Southampton Southampton UK; ^3^ International Institute of Tropical Agriculture (IITA) Nairobi Kenya; ^4^ National Agricultural Research Organization (NARO) Kampala Uganda; ^5^ National Forestry Resources Research Institute (NAFORI) Kampala Uganda; ^6^ Institute for Biochemistry and Biology, University of Potsdam Potsdam Germany; ^7^ Leibniz Institute for Agricultural Engineering and Bioeconomy (ATB) Potsdam Germany

**Keywords:** 16S rRNA/ITS amplicon sequencing, African herbal medicine, *M. whitei*, phytochemicals, plant microbiome, Sub‐Saharan Africa

## Abstract

*Mondia whitei* (Hook.f.) Skeels is an essential medicinal plant in African societies. However, little is known about the plant's metabolome and its microbiota. Here, we examine the root endosphere and rhizosphere from five locations in Uganda using high‐throughput amplicon sequencing, qPCR and multifactorial modelling. Root metabolite profiles obtained with GC/LC–MS were comprehensively catalogued through a deep literature survey using 516 sources. Plant roots were inhabited by microbiota ranging between 50 and 500 ASVs, also with an average microbial abundance of 10^11^ gene (16SrRNA or ITS) copies per gram. The microbiota was dominated by *Gammaproteobacteria*, *Alphaproteobacteria* and *Actinobacteria,* as well as *Sordariomycetes*, *Dothideomycota*, *Eurotiomycetes* and *Agaricomycetes*. We identified that a major portion of the microbiome (i.e., 45%–70%) was potentially transferred from the rhizosphere into the roots. Therefore, the root microbiota showed a strong location‐specific microbial and metabolite fingerprint. Fraxin, 4‐methoxy‐benzaldehyde, monobutyl phthalate, 2‐hydroxy‐4‐methoxybenzaldehyde, and scopoletin were among the 86 compounds found in plant roots that were strongly correlated with the root microbiota. Moreover, the identified plant compounds have been shown to mediate microbe, plant, and animal interactions. Our research advances the research frontiers of endangered African herbal plants, through providing insights into the microbiome and potential compounds of *M. whitei* , a medicinal plant used in sub‐Saharan Africa.

## Graphical abstract

Mondia whitei is a much sought‐after medicinal plant in Sub‐Saharan Africa, yet little is known about its metabolome, and even less about its microbiome composition. Here, we examined the plant's root‐associated microbiota and metabolites using high‐throughput sequencing and GC/LC‐MS. The findings reveal a location‐specific microbiome, core microbial taxa, and metabolite‐microbiome correlations, advancing knowledge of the plant's medicinal potential and bioprospecting.

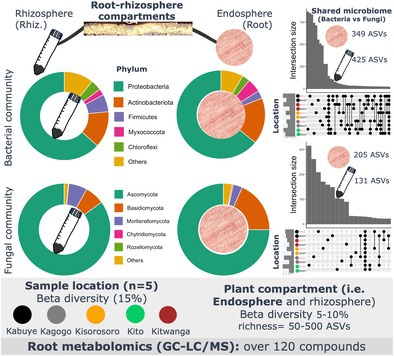

## 1 Introduction

Africa harbours highly diverse microbiomes which have remained underrepresented in global plant, soil, and human microbiome databases (Makhalanyane et al. 2023). Characterizing such microbiomes could uncover the secrets underlying their importance. Traditional herbal medicine has for generations contributed to the health and resilience of indigenous African communities that derive income from and herbal treatments for various ailments (Farnsworth et al. 1985). A classic example involves the gathering and consumption of *M. whitei* (Hook.f.) Skeels, a herbal medicinal plant that is endemic to Sub‐Saharan Africa, where it naturally grows in the wild, but also in arable land (Venter et al. 2009). The plant is a perennial herbaceous climber belonging to the family *Apocynaceae* and subfamily *Periplocoideae*. It comprises of large heart‐shaped light green leaves and clusters of white flowers, and mature plants can reach around 3–6 m in length (Ross 1978). Owing to the extensive collection of wild *M. whitei* roots, the plant is categorized as endangered, justifying the need for research aimed at conservation and the sustainable utilization of naturally existing populations (Baskaran et al. 2015).

The consumption of *M. whitei* roots among African communities has been associated with aphrodisiac effects, especially in males (Oketch‐Rabah 2012). The roots are consumed raw and can be used to make tea or a beer‐like drink, while plant leaves are ground into powder for use as a dietary supplement (Oketch‐Rabah 2012). The other benefits of the plant include its use as a spice and flavouring agent, and as a herbal remedy for: (i) the induction of labour (Ssegawa and Kasenene 2007); (ii) stomach ache (Venter et al. 2009); (iii) malaria infections (Odugbemi et al. 2007); (iv) intestinal worm infections (anthelmintic) (Idu et al. 2010); and stress relief (Wyk and Gericke 2000). A survey conducted in Kenya revealed the potential role of *M. whitei* in deworming, and for the treatment of ringworm fungal infections, asthma, and heart disease (McGeoch 2004). Moreover, mild anti‐bilharzia effects of *M. whitei* extracts (Sparg et al. 2000), and a role in the treatment of sickle cell disease has also been reported from the Congo Basin (Central Africa) (Bongo et al. 2017). Owing to regional and cultural importance, various names including “Tonic Roots” or “White's ginger” (by the English), “Umondi or Mundi” (Zulu people), “Mungurawu” (Shona‐Zimbabwe), and “Mulondo” (Uganda) have been given to *M. whitei* .

The effects described are not only based on empirical observations, but also include a myriad of pharmacological studies on *M. whitei* across various African countries. For instance, there is a growing understanding of the aphrodisiac, antimicrobial, and anti‐inflammatory properties of *M. whitei* (Aremu et al. 2011), yet the wider metabolome may potentially underlie its biological activity, which might also explain the plant microbiome assembly, remains unexplored. Moreover, the potential contribution of the native soil microbiome to plant metabolite production has also been little studied.

Plant‐metabolome‐microbiome interplay is initiated early during plant development (Abdelfattah et al. 2023). Here, the foundational plant microbiome is established through vertical transmission from parent plants and horizontal acquisition from the environment. During development, plants influence the soil microbiome through establishment of the rhizosphere. The rhizosphere forms the primary interface for dynamic metabolic exchange, where plants actively shape microbial communities by secreting diverse root exudates, including aromatic organic acids and secondary metabolites (Wu et al. 2023). These interactions are inherently bidirectional: while plants modulate microbiome composition through targeted metabolite secretion, the established rhizosphere microbiota, in turn, influence plant metabolism by altering root exudate profiles and secondary metabolite production, forming a complex, feedback‐driven system (Pang et al. 2021). However, we are just at the beginning of understanding the complex plant–microbe feedbacks, especially those of traditionally important medicinal plants such as *M. whitei* .

The roots of *M. whitei* have a strong vanilla‐like aroma, which has been attributed to the volatile compound 2‐hydroxy‐4‐methoxybenzaldehyde (Kubo and Kinst‐Hori 1999); while the other bioactive compounds, which are associated with the plant, include epinephrine, norepinephrine, dopamine, serotonin, gamma‐aminobutyric acid, and coumarinolignans (Bunel et al. 2014). Interestingly, in Germany, under the patent DE102009046126A1, the root extract of *M. whitei* , containing 2‐hydroxy‐4‐methoxybenzaldehyde, is used in orally consumable preparations for cosmetics. Plants regularly host a highly abundant and diverse microbiome that provides critical functions, such as biotic and abiotic stress resilience (Bulgarelli et al. 2012; Fitzpatrick, Copeland, et al. 2018). The microbiome is influenced by plant genotype, compartment type, stage of growth, and environmental conditions in a location, like soil physico‐chemical parameters (Xiong et al. 2021). The rhizosphere microbiome is highly diverse and influenced by the plant chemistry (Qi et al. 2012). Moreover, the plant microbiome may contribute to the production of phytotherapeutic compounds (Köberl 2013). Therefore, investigating the links between the plant microbiome and the plant metabolome may offer strategies for the sustainable use of plant resources and uncover their biotechnological potential.

**FIGURE 1:**
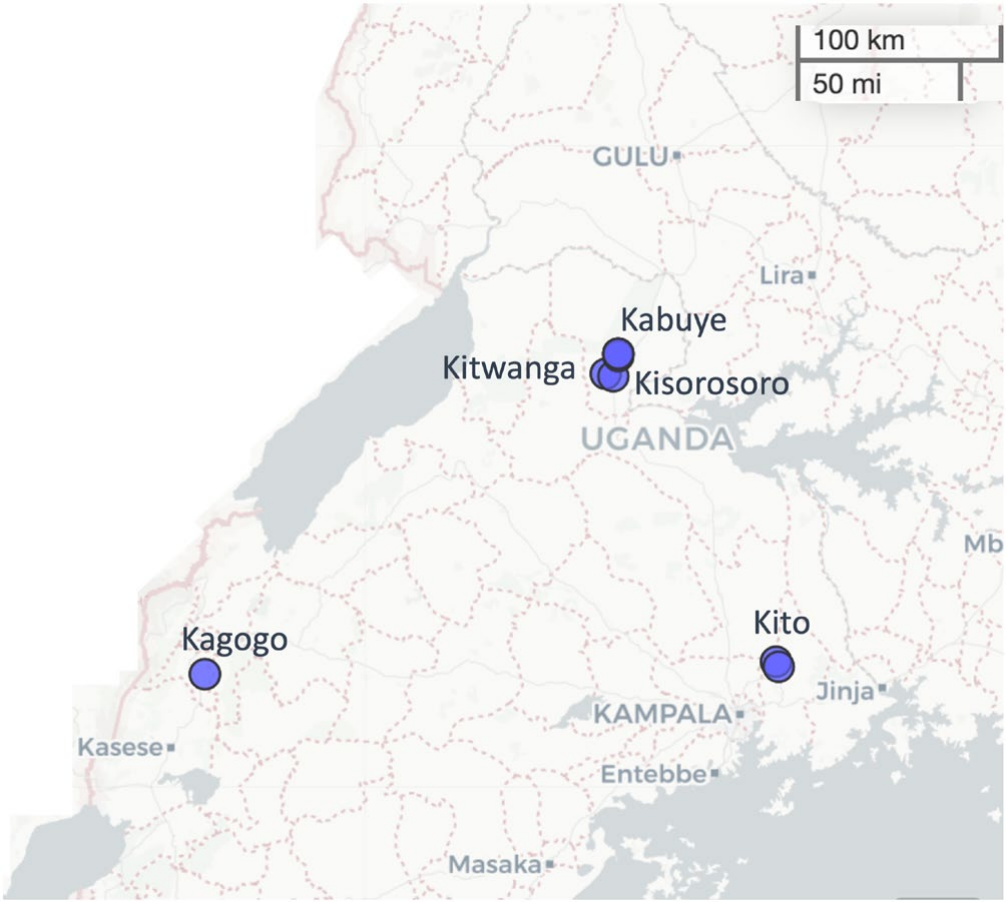
A section of the map of Uganda representing the locations where *M. whitei* roots were obtained. The points on the map represent locations including Kagogo, Kabuye, Kitwanga, Kisorosoro, and Kito, respectively.

Here, we employed amplicon sequencing of bacterial and fungal markers (16S rRNA gene and ITS region, respectively), combined with gas/liquid chromatography followed by mass spectrometry (GC/LC–MS) to examine the microbiota and metabolome of *M. whitei* samples, which were obtained from five geographical regions in Uganda (Figure 1). The study is based on the conjecture that: (i) the plant microbiota is shaped by the root metabolite profiles of *M. whitei* , which could also be of medicinal importance, (ii) the plant microbiome and metabolite profiles are specific across spatial scales (i.e., compartment and regional scales), and that (iii) a common microbiome exists across spatial scales. The current study examines the *M. whitei* microbiome and offers insights into the plant root metabolome and its potential functions.

## 2 Materials and Methods

### 2.1 Sampling and Site Descriptions

Root and rhizosphere samples were obtained from five locations in Uganda. The five locations included: Kabuye (31.9967°N, 1.8129°E), Kitwanga (32.0228°N, 1.7984°E), Kisorosoro (32.0466°N, 1.9014°E), Kagogo (30.2371°N, 0.4914°E), and Kito (32.7477°N, 0.5319°E); corresponding to regions of Uganda including: (i) mid‐west (Kabuye, Kitwanga, and Kisorosoro), (ii) west (Kagogo), and (iii) central (Kito) Uganda (Figure 1). These regions were chosen because of the wide occurrence and use of the plant. For each location, eight biological replicates were obtained and processed to obtain the root and rhizosphere compartments. Briefly, root samples were obtained from the ground using a shovel and stored in water‐resistant polythene bags. The samples were kept in a cool box containing ice packs before preparation for international transport to the Institute of Environmental Biotechnology (Graz, Austria), where they were further processed. For locating plant samples, we relied on the expert guidance of scientists at Uganda's National Agricultural Research Organization and the International Institute of Tropical Agriculture.

### 2.2 Sample Processing

A 5 g sample of root with attached rhizosphere was transferred into a sterile stomacher bag containing 5 mL of 0.85% sodium chloride. The bags were shaken for 30 s using a bag mixer (Interscience, St. Nom, France) to dislodge the rhizosphere. The rhizosphere solution was transferred into 15‐mL tubes, centrifuged (5000 g, 10 min) to obtain the rhizosphere pellet. After dislodging the rhizosphere, root samples were further washed with 0.85% sodium chloride solution, pulverized with liquid nitrogen, and ground with a mortar and pestle. The rhizosphere pellet and the crushed root samples were stored at −70°C for DNA extraction. The DNA was extracted using the FastDNA Spin Kit for Soil (MO BIO Laboratories, USA), following the manufacturer's instructions. The DNA was extracted from 0.5 g of the processed root and 0.2–0.8 g rhizosphere pellet samples. The DNA concentration was estimated using the NanoDrop 2000 spectrophotometer (Thermo Fischer Scientific, Germany), and the DNA was stored at −20°C. The same samples were used for GC–MS (Method S1) and HPLC–MS analyses (Method S2).

### 2.3 Determining Microbial Abundance (qPCR) in the Root and Rhizosphere

We estimated the microbial abundance in the root/rhizosphere samples of *M. whitei* using quantitative real‐time PCR (qPCR). The primer pair Unibac‐II‐515f/Unibac‐II‐806r (10 μM) was used to quantify bacterial 16S rRNA genes, as previously described (Caporaso et al. 2011); while ITS1f/ITS2r (10 μM) was used for the estimation of fungal abundance (White et al. 1990). Reactions were performed in a total volume of 10 μL, containing 5.0 μL of KAPA SYBR Green (Bio‐Rad, Hercules, CA, U.S.A.), 0.5 μL of each PCR primer pair, 3 μL of PCR‐grade water, and 1 μL template DNA. Samples were diluted 1:10 using PCR‐grade water. The PCR‐grade water was used as the negative control. All reactions were performed in duplicates for each sample on a Rotor‐Gene 6000 series thermocycler (Corbett Research, Australia). The cycler conditions included: initial denaturation (95°C, 5 min) followed by 35 cycles of denaturation (95°C, 10 s), annealing (54°C, 15 s), extension (72°C, 10 s), and melt down from 72°C to 96°C. As standards, we used *Bacillus* sp. and *Penicillium* sp. for bacterial and fungal community, respectively.

### 2.4 Amplicon Library Preparation

The two‐step PCR procedure involving the amplification of marker genes for bacterial (16S rRNA gene) and fungal (ITS region) communities, as well as the subsequent attachment of sample‐specific barcodes to generate amplicon libraries for bacterial and fungal microbial communities. In the first step, the hypervariable V4 region of the 16S rDNA was amplified using the PCR primer pair: 515f‐pad (5′–CTTGGTCATTTAGAGGAAGTAA–3′) and 806r‐pad (5′–GCTGCGTTCTTCATCGATGC–3′) (Caporaso et al. 2011), as well as the fungal ITS1 and ITS2 regions with primers: ITS1f‐pad (5′–CTTGGTCATTTAGAGGAAGTAA–3″) and ITS2r‐pad (5′–GCTGCGTTCTTCATCGATGC–3′) (White et al. 1990). In the second step, the amplified gene product from the first PCR was indexed with 12‐nucleotide‐long, sample‐specific barcodes.

Peptide nucleic acid (PNA) PCR clamps were used to block the amplification of plastid and mitochondrial 16S rRNA genes during the PCR amplification of the bacterial community (Lundberg et al. 2013; Fitzpatrick, Lu‐Irving, et al. 2018). In the first PCR step for amplification of bacterial 16S rDNA, a 10 μL reaction containing 6.5 μL PCR‐grade water, 0.1 μL (10 μM forward/reverse) primers, 0.15 μL mPNA/pPNA (50 μM) primers, 2.0 μL (5xTaq and Go, MP Biomedicals), and 1 μL of template DNA was used. The cycler program included a preheating step (95°C, ∞), initial denaturation (95°C, 5 min), followed by 25 cycles of denaturation (95°C, 45 s), PNA step (78°C, 5 s), annealing (55°C, 45 s), strand extension (72°C, 90 s), final extension (72°C, 5 min), and final hold (10°C, ∞). Similarly, for the fungal community, the first PCR was performed in a 10 μL volume containing 5.6 mL PCR‐grade water, 1.2 μL MgCl_2_ (25 mM), 0.1 μL (ITS1f/ITS2r primers), 2.0 μL (5xTaq and Go, MP Biomedicals), and 1 μL DNA template. The cycler conditions included: preheating (9°C, ∞) and initial denaturation (95°C, 5 min), followed by 35 cycles of denaturation (95°C, 30 s), annealing (55°C, 35 s), extension (72°C, 40 s), final extension (72°C, 10 min), and final hold (10°C, ∞).

After successful amplification in the first PCR, the second PCR was performed to attach sample‐specific barcode sequences. These reactions were performed in triplicates in 30 μL volume, which contained 19.5 μL PCR‐grade water, 1.2 μL of the barcode primers (10 μL forward/reverse barcodes), 6.0 μL (5xTaq and Go, MP Biomedicals), and 2 μL of template DNA. As a negative control, PCR‐grade water was used. The cycler conditions included a preheating step (95°C, ∞), and initial denaturation (95°C, 5 min), followed by 15 cycles of denaturation (95°C, 30 s), annealing (53°C, 30 s), extension (72°C, 30 s), final extension (72°C, 5 min), and final hold (10°C, ∞). After the first and second PCRs, amplicon fragment lengths were validated by agarose‐gel electrophoresis, and the correct band sizes corresponding to the targeted genes were confirmed. All the reactions were performed in a thermocycler (Eppendorf Thermocycler Nexus GX2, Hamburg‐Germany). The PCR products were purified using the QIAquick gel extraction kit (Qiagen). All samples were pooled in equimolar concentrations and subjected to paired‐end Illumina MiSeq 2 × 300 sequencing at Novogene (Cambridge Science Park, United Kingdom).

### 2.5 Bioinformatic Analysis

Paired‐end reads were quality‐checked and demultiplexed using Cutadapt (Martin 1994). Demultiplexed reads were quality filtered, trimmed, denoised, merged, followed by chimera removal using the DADA2 algorithm (Callahan et al. 2016) through the open‐source QIIME 2 (version 2021.11.0) (Bolyen et al. 2019); thus generating amplicon sequence variants (ASVs) and the table of ASV counts. Taxonomic assignment of denoised reads was performed using VSEARCH in QIIME 2 by comparing the reads against reference databases SILVA132 for 16S rRNA (Quast et al. 2013) and UNITE v7 (Abarenkov et al. 2024) for fungal‐associated sequences, using q2‐feature‐classifier (Bokulich et al. 2018). The taxonomic assignment resulted in a taxonomy table. The obtained microbial ASV tables and taxonomic assignment were processed using phyloseq (McMurdie and Holmes 2013) and vegan 2.5.7 (Oksanen et al. 2020) packages. The non‐bacterial ASVs that were associated with chloroplast, mitochondria, and Archaea were removed, resulting in 2,684,660 and 2,660,423 high‐quality reads; that comprised 11,522 bacterial and 2888 fungal ASVs. We used the *rarefy even depth* and *ggrare* functions in the Ranacapa package to rarefy the dataset and to generate the rarefaction curves (Figure S1). The ASV tables were subsampled to 3000 and 5000 reads per sample for the estimation of bacterial and fungal alpha diversity (i.e., richness and Shannon indices); wherein three and five samples were removed for bacterial and fungal communities, respectively. Beta‐diversity analyses were performed on normalized microbiome datasets using cumulative sum scaling (CSS) (Weiss et al. 2017). Both alpha‐diversity and beta‐diversity were calculated using the phyloseq package in R (version 4.0.3). All the raw sequencing data have been submitted to the National Centre for Biotechnology Information (NCBI) BioProject PRJNA1297068 (https://www.ncbi.nlm.nih.gov/bioproject/1297068).

### 2.6 Statistical Analysis, Detection of Indicator ASVs

The statistical analyses were carried out in R (http://www.r‐project.org) using RStudio (version 1.1.423). The Kruskal‐Wallis test (i.e., for non‐parametric datasets) for examining the overall statistical differences in Shannon index, richness (observed ASVs), and microbial abundance across the different compartments (i.e., rhizosphere and root endosphere) and sample locations. Dunn's test with correction for multiple comparisons using the Bonferroni method was used to compare compartment‐specific differences in alpha diversity and microbial abundance across locations. For each location, pairwise comparison between compartments was performed using the Wilcoxon Signed Rank test. The effect of geographic location and compartment on the microbial community structure (i.e., Beta diversity) was tested by permutational analysis of variance (i.e., PERMANOVA, 999 permutations) in the vegan package 2.5.7 (Oksanen et al. 2020), using Bray‐Curtis dissimilarities of CSS normalized microbiome count datasets, followed by principal coordinate analysis (PCoA) for ordination analyses. The plot bars function in phyloseq was used to visualize taxonomic composition in the different compartments and locations. Furthermore, the compartment‐specific common microbiome was performed using an abundance‐ and occupancy‐based approach, where the core seed microbiome spanning prevalence thresholds ranging between 25% and 100% of the samples and the minimum detection thresholds (so‐called relative abundances) ranging between 0.00001% and 1% was determined using the unrarefied dataset. We explored using the SourceTracker script in R (Knights et al. 2011) the proportion of root‐enriched microbiota in different locations by considering the rhizosphere as the microbial source and the root compartment as the microbiota sink.

### 2.7 Differential Abundance Calculations and Metabolite Associations

We selected microbial ASVs with differential abundances between root and rhizosphere using DESeq2 (Love et al. 2014). The differentially enriched endosphere bacterial and fungal ASVs were then ranked using a random forest classifier (RFC) based on their importance for discerning the geographical location of the samples (*n* = 40). The rarefied ASV proportions were transformed using the centred log‐ratio and zeros were imputed using multiplicative replacement (Martın‐Fernandez et al. 2003). The RFC was trained on all data and hyperparameters were optimized using a grid search with five‐fold cross validation. The importance of the ASVs for classifying geographic location was measured using shapely additive explanations (Lundberg et al. 2020). To model associations between compounds and the root microbiome assembly (*n* = 20), we used multitask elastic net regression with leave‐one‐out cross validation. The metabolite (GC/LC–MS) data was standardized per compound (z‐score) and used as predictor variables for fitting the centered log ratio of the differentially abundant ASVs. The model coefficients were clustered hierarchically to visualize (Waskom 2021) associations of the ASVs and metabolites. Potential roles of the relevant bioactive compounds identified during GC–MS and HPLC analysis were surveyed from literature using the PubChem database (Kim et al. 2021).

## 3 Results

### 3.1 *M. whitei* Microbiome Differs Between Geographic Regions

**FIGURE 2:**
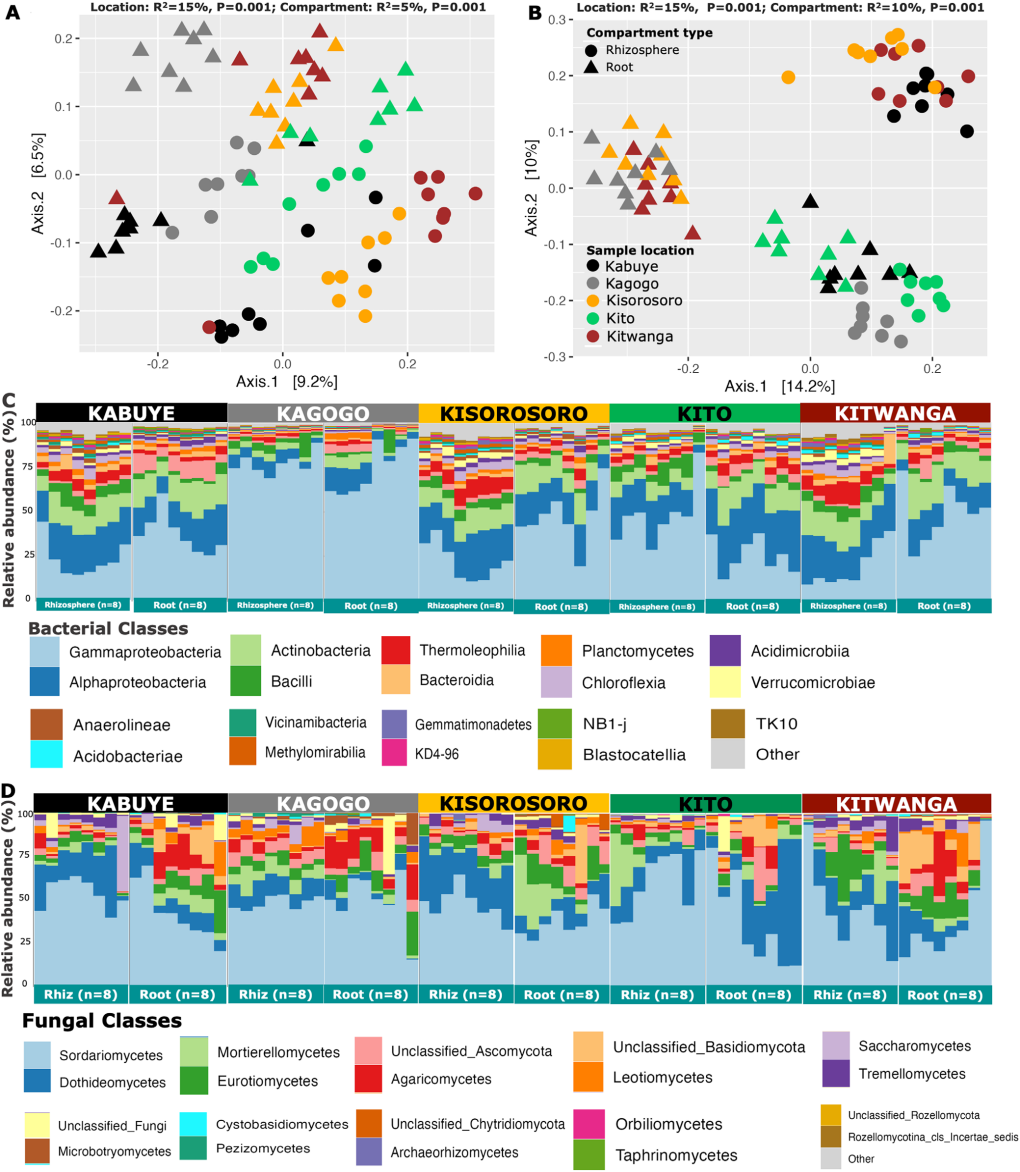
Microbial community composition in the root and rhizosphere across sample locations. Panel (A) and (B) are Principal Coordinates Analysis (PCoA) representations of Bray–Curtis dissimilarity matrices showing the bacterial and fungal community structure, respectively. Significant bacterial variation explained by sample location and compartment is represented by R^2^ = 15% and R^2^ = 5%, while the fungal community is represented by R^2^ = 15% and R^2^ = 10%, respectively. Panels C and D are stacked barplots showing the percentage average taxonomic composition of the bacterial and fungal community at the class level. The plant samples (i.e., root or rhizosphere) are represented by shapes and colours represent sample location.

Data from five geographic locations were analysed using cumulative sum scaled microbiome datasets, followed by PERMANOVA of Bray–Curtis dissimilarity to test for differences in root endosphere and rhizosphere microbiomes (*n* = 80). Geographic location (i.e., sample location) was the major factor shaping the bacterial and fungal community composition (bacterial: R^2^ = 15%, *p* = 0.001 and fungal: R^2^ = 15%, *p* = 0.001). Plant compartment also contributed to community variations (bacterial: R^2^ = 5%, *p* = 0.001; fungal: R^2^ = 10%, *p* = 0.001), but to a lesser extent when tested together with geographical location. Additionally, there was a significant interactive effect of location and plant compartment on the microbial community composition (bacterial: R^2^ = 8%, *p* = 0.001; fungal: R^2^ = 12%, *p* = 0.001). Pairwise significant differences (*p* < 0.05) between geographic locations for the different compartments are shown in Table S1. Principal coordinate analysis (PCoA) was used to visualize microbiome clustering by geographic location and plant compartment for bacterial (Figure 2A) and fungal communities (Figure 2B), where community separation between the root and rhizosphere compartments was shown for bacterial (Figure 2A) and fungal communities (Figure 2B) across the different sample locations. For the bacterial community, three out of the eight rhizosphere samples that were obtained from Kabuye clustered further from the rest of the samples (Figure 2A).

The root endosphere and rhizosphere bacterial communities were dominated by phyla *Proteobacteria*, with average percentage abundances in rhizosphere (60%) and root endosphere (67%), *Actinobacteria* (14%; 16%), *Firmicutes* (4%; 2%), *Bacteroidetes* (5%; 2%), *Myxococcota* (3%; 4%), and *Acidobacteria* (2%; 2%); as well as the dominance of bacterial classes *Gammaproteobacteria* (41%; 52%), *Alphaproteobacteria* (19%; 13%), and *Actinobacteria* (8%; 11%) (Figure 2C). The class *Gammaproteobacteria* was higher in the root compartment compared to rhizosphere, except for the locations including Kagogo and Kito, which revealed a high composition of *Gammaproteobacteria* in the rhizosphere (Figure 2C). The fungal community was dominated by phyla *Ascomycota* (rhizosphere: 88%; root: 75%), followed by *Bacidiomycota* (7.4%; 16%) and *Mortierellomycota* (4.5%; 5%), with the predominance of classes *Sordariomycetes* (62%; 51%), *Dothideomycota* (15%; 12%), *Eurotiomycetes* (4%; 7%), *Agaricomycetes* (2.7%; 7.0%), and *Mortierellomycetes* (4.5%; 4.7%) (Figure 2D). In comparison to the root endosphere, rhizosphere was associated with a high proportion of *Sordariomycetes* for all locations (Figure 2D). Except for Kito, all locations contained a high proportion of *Dothideomycetes* (Figure 2D), while unclassified (*Basidiomycota* and *Ascomycota*) were particularly higher in the root endosphere as compared to rhizosphere (Figure 2D).

### 3.2 The Difference in Bacterial Diversity Was Majorly Influenced by Sample Location, Whereas Fungal Diversity Was Predominantly Shaped by Compartment

**FIGURE 3:**
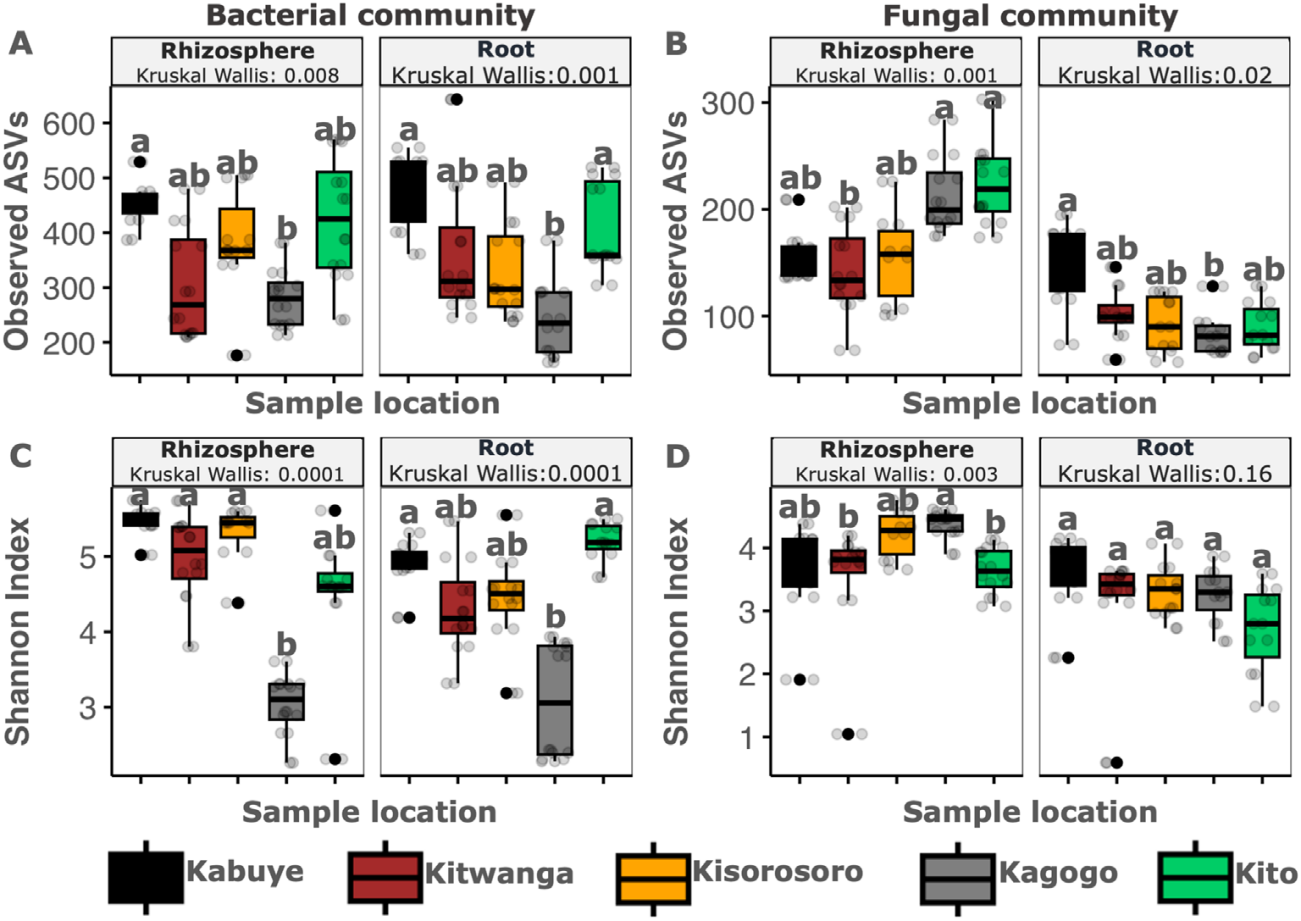
Microbial alpha diversity significantly varied across sample locations in a compartment‐specific manner. Panels (A) and (B) show the bacterial and fungal richness (i.e., observed ASVs); (C) and (D) show the Shannon diversity for bacterial and fungal communities, respectively. The boxplots represent the distribution of samples (*n* = 8 samples) for each compartment with the estimation of the median, 25th, and 75th percentiles. *p*‐values were determined using the Kruskal‐Wallis test. The letters above the boxplots represent levels of significance (*p* ≤ 0.05), while data points outside the boxplot area represent outliers. Legend colours represent the different sample locations.

To compare the microbial alpha diversity across locations and compartments (i.e., root endosphere and rhizosphere), the Kruskal‐Wallis test was performed on alpha diversity indices (i.e., Shannon index and richness). Generally, there were significant differences in bacterial richness (*p* = 4 × 10^−6^) and Shannon index (*p* = 3 × 10^−7^) across sample locations, and no significant differences were observed across compartments. For fungal communities, significant variations in richness (*p* = 1 × 10^−8^) and Shannon index (*p* = 1 × 10^−6^) were observed between compartments, with no significant differences observed between locations. Analysis of the compartment‐specific effect of sample location on microbial diversity revealed significant variation in bacterial community richness and diversity across locations in the rhizosphere and root endosphere (Figure 3A,C). For the fungal community, significant differences between locations were observed in the rhizosphere (Figure 3B,D). Moreover, for each location, pairwise comparison of alpha diversity indices between compartments (using Wilcoxon Signed Rank test) revealed significantly higher (*p* ≤ 0.05) fungal richness and Shannon indices in the rhizosphere as compared to the root endosphere for locations: Kagogo, Kisorosoro, Kito, and Kitwanga, respectively (Figure S2B,D). Significantly higher bacterial Shannon indices were observed in the rhizosphere than in the root endosphere for Kabuye and Kisorosoro (Figure S2C).

The overall average microbial abundance was similar for the rhizosphere (i.e., 6 × 10^9^ to 10 × 10^11^) and endosphere (i.e., 1 × 10^10^ to 6 × 10^10^) (Figure S2). There were significant differences in bacterial abundance between locations, both in the rhizosphere (Kruskal‐Wallis; *p* = 0.00002) and root (Kruskal‐Wallis; *p* = 0.002) compartments, respectively (Figure S3A). For the fungal community, significant differences in fungal abundance were seen only with the root compartment (Figure S3B). Pairwise comparisons using the Wilcoxon Signed Rank test revealed significantly higher bacterial abundance in the rhizosphere as compared to roots for locations like Kagogo and Kito, and a significantly higher bacterial abundance in roots than the rhizosphere for Kabuye (Figure S3C). Meanwhile, the fungal abundance was significantly higher in the root endosphere as compared to rhizosphere for samples obtained only for samples obtained from Kagogo (Figure S3B).

### 3.3 *M. whitei* Root Endosphere Microbiome Is Likely Internalized From the Rhizosphere

The core microbiome (i.e., prevalence 25%–100% and abundance ranging between 0.00001% and 1%) of *M. whitei* across the five locations and compartments was composed of bacterial genus *Noviherbaspirillum* (Figure S4A), as well as fungal genera, like *Fusarium*, *Mortierella*, *Penicillium*, *Hannaella*, and *Vashiniacozyma* (Figure S4B). Overall, the rhizosphere was associated with 349 bacterial and 205 fungal ASVs (Figure S4C,D, respectively), as well as the presence of 425 bacterial and 131 fungal ASVs in the root endosphere (Figure S4E,F). Of these, 48 and 56 bacterial ASVs, as well as 28 and 10 fungal ASVs were shared across locations, both for the rhizosphere and root compartments, respectively. The shared ASVs in the root endosphere and rhizosphere mainly belonged to bacterial genera *Pseudomonas, Mycobacterium, Streptomyces, Bacillus, Nitrospira, Phenylobacterium, Sphingomonas, Sphingobium, Bradyrhizobium, Ralstonia*, and *Pantoea*. Core rhizosphere fungal genera were identified as *Metarhizium*, *Fusarium, Mortierella, Purpureocillium, Cercospora, Stagonosporopsis, Hannaella*, and *Vishniacozyma* (Table S2). The core endosphere fungal microbiota include *Fusarium* and unclassified (*Xylariales*, *Eurotiomycetes*, *Auriculariales*, and *Ascomycota*) (Table S2). Furthermore, we examined the proportion of the endosphere microbiome that was potentially internalized from the rhizosphere into the root, using SourceTracker (Knights et al. 2011). Analyses revealed that 80%–95% of the bacterial and 65%–90% of fungal microbiota were potentially transferred from the rhizosphere into the plant roots (Figure S5). The percentage of root endosphere microbiota from unknown sources was 5% and 25% for bacterial and fungal community, respectively.

### 3.4 The Root Endosphere Microbiota Is Influenced by Plant Location and Enriched from the Rhizosphere

**FIGURE 4:**
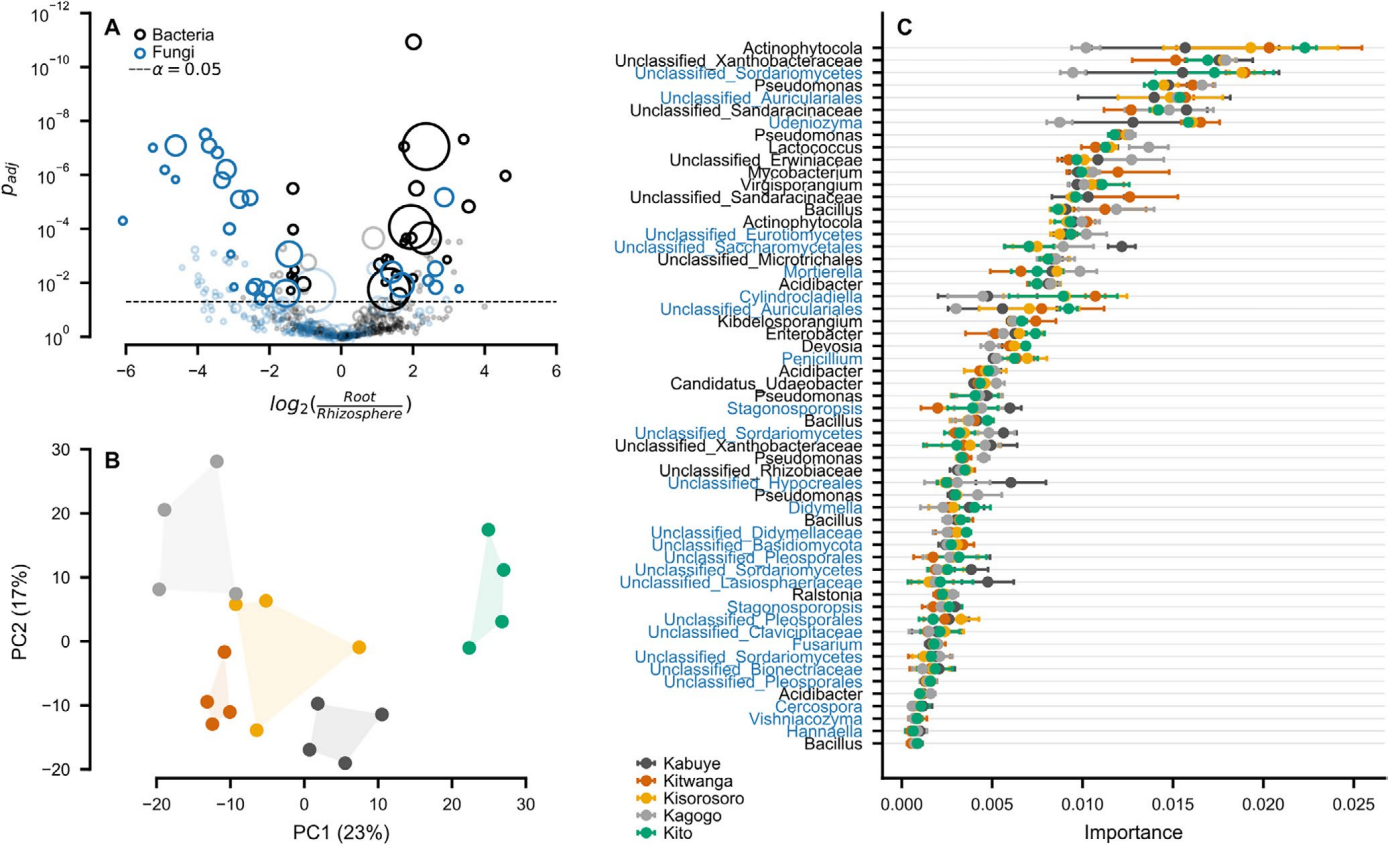
*M. whitei* root endosphere microbiome can delimit geographical locations. Panel A shows the differential microbial abundance in root relative to the rhizosphere, determined using DESeq2 and feature selection based on *p*‐values, log2 (fold‐change) and mean abundances for bacterial (open dark circle) and fungal community (open blue circle), respectively. The size of the circle represents abundance of the differentially enriched root taxa. Panel B shows community clustering of the selected taxa across different locations, where the points represent sample locations. Panel C shows the most important of microbial classes for the classification of geographic locations, using a random forest classifier, including the modelling of association between compounds and the root microbiome by multitask elastic net regression with leave‐one‐out cross validation.

We compared the root endosphere and rhizosphere microbiomes using DESeq2 for differential abundance testing (Love et al. 2014). The ASVs were considered enriched (or depleted) at *α* ≤ 0.05, baseMean > 5, and absolute log_2_ fold‐change > 1 (Figure 4A). Overall, 22 bacterial and 7 fungal ASVs were significantly enriched in the root endosphere and 7 bacterial, and 21 fungal ASVs were more abundant in the rhizosphere. Selecting only the differentially abundant ASVs from the data, resulted in clear clustering of the microbial communities depending on geographic location (Figure 4B). Using random forest, we classified the different geographic locations based on the selected ASVs with a test accuracy of 0.9 ± 0.05 (*n* = 40). The ASVs were ranked in order of importance to the outcome of the model classification (Figure 4C). The most important ASVs for classifying the different geographic locations belonged to bacterial genera *Actinophytocola*, unidentified *Xanthobacteraceae*, *Pseudomonas*, *Lactococcus*, *Bacillus*, *Mycobacterium*, *Virgisporangium*, *Kibdelosporangium*, *Enterobacter*, *Devosia*, and *Acidibacter* (Figure 4A). The most important fungal genera that are potential delimiters of sample location included *Penicillium, Cylindrocladiella, Stagonosporopsis, Didymella, Mortierella*, Udeniozyma, *Fusarium* as well as unclassified (*Sordariomycetes*, *Auriculariales*, *Eurotiomycetes*, *Hypocreales*, and *Pleosporales*) (Figure 4C).

### 3.5 Distinct Members of the Plant Microbiome Were Correlated With Root Metabolites

**FIGURE 5:**
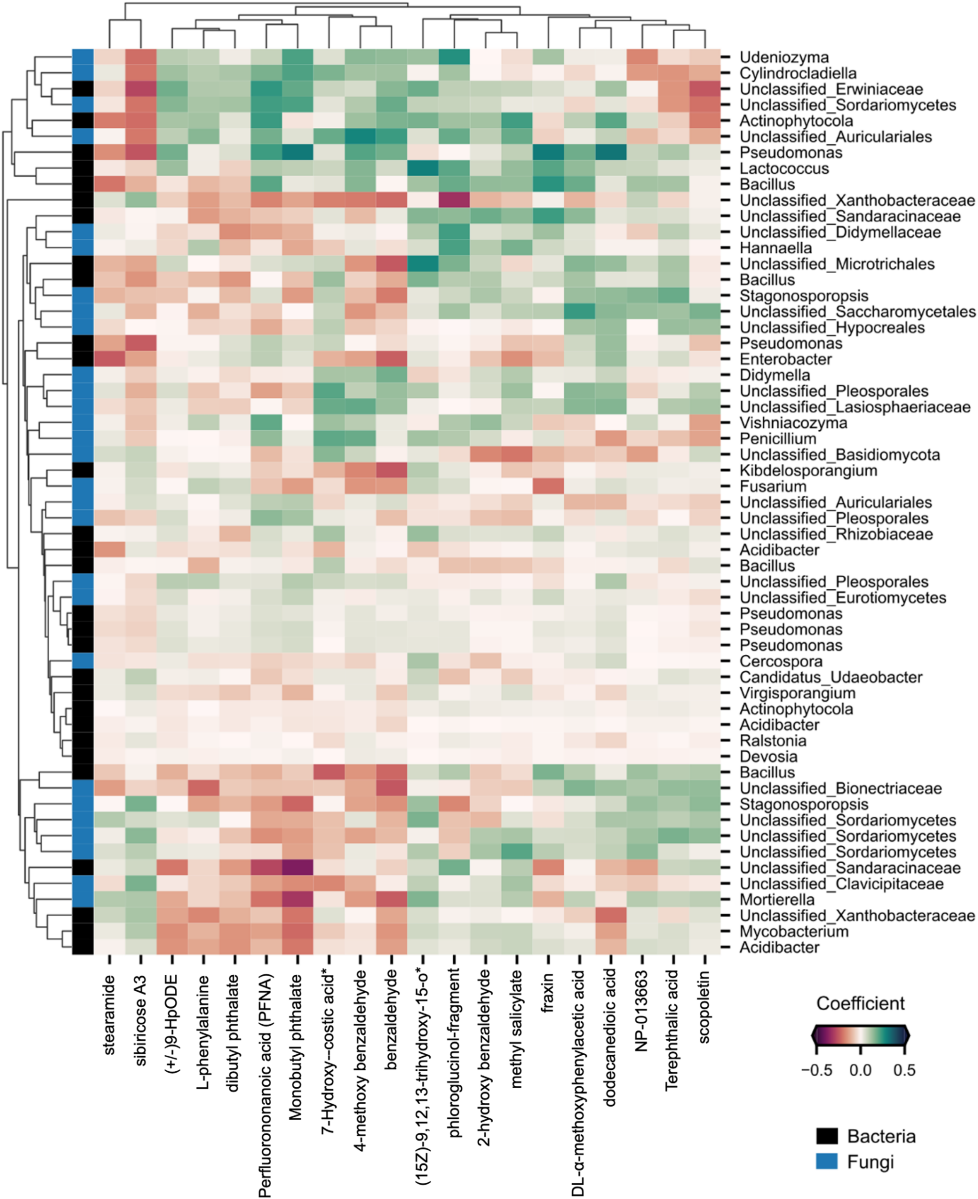
The *M. whitei* root metabolome and the associated microbiome. The clustered heatmap shows the marginal coefficients between root compounds and root‐enriched microbiota. The compound labels with (*) extend as follows: 7‐Hydroxy‐costic acid* [2‐[(2S,4aR,8aS)‐2‐hydroxy‐4a‐methyl‐8‐methylidene‐3,4,5,6,7,8a‐hexahydro‐1H‐naphthalen‐2‐yl]prop‐2‐enoic acid] and (15Z)‐9,12,13‐trihydroxy‐15‐o* [(15Z)‐9,12,13‐trihydroxy‐15‐octadecenoic acid]. The colour scale represents the coefficient between root‐enriched microbiota and metabolites.

The root metabolome profile was composed of 120 compounds which were used for correlation analysis with microbiome datasets. We modelled the associations of differentially abundant ASVs with the metabolite profiles of root samples using multitask elastic net regression with leave‐one‐out cross validation (R^2^ = 0.53, *n* = 40) (Figure 5). The obtained model coefficients were hierarchically clustered to identify the groups of compounds that had the strongest associations with the root microbiome (Figure 4). Compounds such as dodecanoic acid, fraxin, perfluorononanoic acid (PFNA), monobutyl phthalate, and 4‐methoxy‐benzaldehyde showed strong correlation with bacterial genera *Lactococcus, Pseudomonas*, and *Bacillus* (Figure 5D). Fungal genera such as *Mortierella, Stagonosporopsis*, and unclassified (*Sordariomycetes*, *Saccharomycetales*, and *Lasiosphaeriaceae*) were correlated to metabolites like dodecanedioic acid, scopoletin, terephthalic acid, sibiricose A3, and methyl salicylate. Benzaldehyde, 4‐methoxy‐benzaldehyde, 2‐hydroxy‐benzaldehyde, and perfluorononanoic acid (PFNA) were correlated with *Penicillium*. Interestingly, compounds including fraxin, benzaldehyde, and 4‐methoxy‐benzaldehyde showed a negative correlation with *Fusarium* (Figure 5).

Basing on extensive literature survey (Table S3), we observed that *M. whitei* metabolome is comprised of various compounds that have antimicrobial activity. Some of these compounds include 4‐methoxy‐benzaldehyde, D‐germacrene, 2‐pentyl‐furan, hexanal, 2‐hydroxy‐4‐methoxy‐benzaldehyde, 3‐hydroxy‐4‐methoxy‐benzaldehyde, 4‐hydroxybenzoic acid, 2‐hydroxy‐benzaldehyde (salicylaldehyde), and fraxetin. Methyl salicylate, one of the most active plant‐immune active chemicals, also with anti‐inflammatory properties in humans, was observed. In addition, Scopoletin (a plant coumarin) with anti‐tumour, anti‐inflammatory, and neuroprotective properties was present in plant roots.

## 4 Discussion

This study revealed insights into the interplay between the microbiome and metabolome within the *M. whitei* holobiont. The plant microbiome composition was majorly influenced by sample location more than by compartment. The microbial diversity also varied across locations, both for the rhizosphere and endosphere. A high proportion of the root endosphere microbiota was found to likely originate from the rhizosphere. The plant's microbiome was also found to be correlated with specific root metabolites, wherein some compounds were specific to the different locations.

The plant microbiome is assembled from seed and the seedling surrounding bulk soil (Abdelfattah et al. 2023; Garrido‐Sanz and Keel 2025), usually in a compartment‐specific manner (Edwards et al. 2015, 2018; Abdelfattah et al. 2021). This creates a microbial gradient along the soil–rhizosphere–endosphere continuum (Edwards et al. 2015), extending into the (root) endosphere (Edwards et al. 2015; Compant et al. 2021). The current findings demonstrate a variation in microbiome composition between the rhizosphere and root endosphere compartments. In comparison to root samples, the rhizosphere was associated with high microbial diversity, in part, due to root exudation (Bais et al. 2006; Badri and Vivanco 2009). The rhizosphere microbiota are essential in mediating various functions, like plant growth and health and pathogen defence (Mendes et al. 2011). The plant microbiome, shaped by biotic and abiotic factors, varies in time and space, which helps explain the observed differences in microbiome composition between the different geographic locations and compartments. The microbiome differences across geographic locations can be explained by the differences in environmental conditions in a location, including the soil physico‐chemical characteristics, which can also shape the root metabolome (Lundberg et al. 2012; Philippot et al. 2024). This highlights the presence of a local microbial and metabolic fingerprint of *M. whitei* roots. We also deduce that *M. whitei* enriches part of the endosphere microbiota from the rhizosphere, which in turn is shaped by the plant's geographic location. The unknown portion of the root microbiota likely is attributed to propagules that propagate the plant, including other sources that we did not examine, such as dust, insects, birds, humans, rain water, wind, etc. *M. whitei* roots are invariably colonized by a high abundance of microorganisms approximating 10^11^ bacterial and fungal gene copies that are potentially ingested for each gram of consumed root. However, the observed high microbial abundance, in part, can be attributed to gene copies originating from chloroplast and mitochondrial DNA.

The plant microbiome mainly consisted of the bacterial phyla *Proteobacteria, Actinobacteria, Bacteroidetes, Acidobacteria*, and *Firmicutes*, as well as the fungal phyla *Ascomycota* and *Basidiomycota*. The microbial community structure of *M. whitei* shows a similar composition to other plant species, like *Arabidopsis thaliana* and *Oryza sativa* (Bulgarelli et al. 2012; Edwards et al. 2015, 2018). The phylum *Proteobacteria* mainly harboured the classes *Gammaproteobacteria* and *Alphaproteobacteria*, as previously shown with other plants (Jiang et al. 2017). In this study, a high prevalence of *Gammaproteobacteria* was especially obvious in the root endosphere compartment. Meanwhile, the dominant fungal classes were *Sordariomycota* and *Dothideomycota*, with *Sordariomycetes* observed particularly in the root as compared to the rhizosphere compartment. The root and rhizosphere samples from different locations shared various taxa, like bacterial genera *Pseudomonas, Mycobacterium, Streptomyces, Bacillus, Nitrospira, Rokubacteriales, Sphingomonas, Sphingobium, Bradyrhizobium, Ralstonia, Pantoea, Solirubrobacter, Vicinamibacteraceae, Nordella, Norcadia*, and *Kribbella*. Bacterial genera such as *Pseudomonas, Bacillus, Streptomyces, Sphingomonas*, and *Bradyrhizobium* have been associated with beneficial roles as biofertilizers and bioprotectants (Santhanam et al. 2015). For instance, *Pseudomonas* spp. strains have been shown to play a crucial role in the suppression of soil‐borne fungal pathogens (Weller et al. 2002). Furthermore, a seed‐endophytic *Sphingomonas melonis* strain was recently shown to induce disease resistance against a soil‐borne pathogen *Burkholderia plantarii* in rice plants ( *O. sativa* L.) (Matsumoto et al. 2021). The shared rhizosphere microbiome was comprised of fungal genera, like *Metarhizium, Fusarium, Mortierella, Purpureocillium, Cercospora, Stagonosporopsis, Hannaella*, and *Vishniacozyma*. Some of these genera, including *Fusarium*, *Mortierella*, *Penicillium*, *Hannaella*, and *Vashiniacozyma*, also formed the core fungal microbiome. Moreover, genera *Metarhizium* (Stone and Bidochka 2020) and *Purpureocillium* (Taleb et al. 2023) contain beneficial strains that are agriculturally important as crop bioprotectants.

In light of the current study, the complex plant–microbiota–metabolome interplay of *M. whitei* might be essential in shaping the plant metabolome or vice versa. This is supported by the observed correlations between the plant metabolome and microbiota. These interactions are important for plants, like in mediating plant defence against pathogens through antibiosis, biofilm formation, competition, and cell lysis (Köhl et al. 2019), also through producing metabolite repertoires, like volatile compounds. The plant microbiome is also linked to the production of bioactive compounds, including those with medicinal potential (Pang et al. 2021); some of which are critical in mediating plant–microbe and microbe–microbe interactions (Zhou et al. 2022; Barone et al. 2024). Previous research showed the contribution of the plant microbiome in the production of compounds with implications for reducing abiotic as well as biotic stresses (Weller et al. 2002; Haas and Défago 2005). The current study showed a strong correlation between bacterial genera *Hyphomicrobium, Bradyrhizobium*, and *Pseudomonas* with *M. whitei* root metabolites, like fraxin, 4‐methoxy‐benzaldehyde, monobutyl phthalate, and 2‐hydroxy‐4‐methoxy‐benzaldehyde; as well as between fungal genera *Penicillium*, *Cylindrocladiella*, and unclassified (*Dothideomycetes*, *Hypocreales*) with dodecanedioic acid, sibiricose A3, scopolectin, octyl‐hydrogen phthalate, and 4‐methoxy‐benzaldehyde. The versatile functions of the plant microbiome can be attributed to some of its root metabolites through the biosynthetic gene clusters of the plant microbiota (Köberl et al. 2013).

Further, the fungal genera *Fusarium, Mortierrella, Stagonosporopsis*, *Candida*, *Roussoella*, and unclassified (*Didymellaceae*, *Pezizales*, *Ceratocystidaceae*) correlated with the compounds perfluorononanoic acid (PFNA), monobutyl phthalate, and 4‐methoxy‐benzaldehyde. *Fusarium* and *Mortierrella* are ubiquitous soil fungi with ecosystem benefits like organic matter decomposition, but also harbour plant pathogens (Zhou et al. 2019; Wang et al. 2022). *Penicillium* is associated with the production of a diverse range of structurally heterogeneous secondary metabolites, which can be of pharmaceutical or industrial importance (Ashtekar et al. 2021; de Carvalho et al. 2021). The fungal genus *Stagonosporopsis* comprises species, such as *S. pogostemonis*, that were recently isolated from *Pogostemon cablin* , a Chinese medicinal plant with pharmacological relevance (Dong et al. 2021). The family *Ceratocystidaceae* comprises genera of significant agricultural importance as saprophytes and plant pathogens (Van der Nest et al. 2015; Mbenoun et al. 2016). Moreover, pathways for metabolite production were recently identified within this family, and associated with the non‐ribosomal peptide synthetases (Sayari et al. 2019). Furthermore, genus *Cercospora* is comprised of species with a known record for secondary metabolite production (Assante et al. 1977; Goodwin et al. 2001).

Notably, the roots of *M. whitei* contain numerous compounds with antimicrobial properties, based on an extensive literature survey. Moreover, wide consumption of the plant's roots by various African communities is partly attributed to their role as a herbal medicine (Oketch‐Rabah 2012). In the current study, we observed a high presence of 2‐hydroxy‐4‐methoxybenzaldehyde, as confirmed by previous research that focused on profiling the plant's root metabolome (Kubo and Kinst‐Hori 1999; Mukonyi and Ndiege 2001). 2‐hydroxy‐4‐methoxybenzaldehyde was characterized as a principal tyrosinase inhibitor, also ubiquitous in medicinal plants, particularly *M. whitei* (Kubo and Kinst‐Hori 1999). Bunel et al. (2014) revealed the high reactivity of 2‐hydroxy‐4‐methoxybenzaldehyde towards dopamine, gamma‐aminobutyric acid, norepinephrine, and serotonin; it is also categorized as an artifactual alkaloid precursor. TeIn related studies, metabolites, such as coumarinolignans (Patnam et al. 2005), tannins and flavonoids (Bouba et al. 2010), and volatile aromatic compounds, like 4‐hydroxy‐3‐methoxybenzaldehyde and 3‐hydroxy‐4‐methoxybenzaldehyde (Mukonyi and Ndiege 2001) showed a high presence in *M. whitei* roots. The metabolite monobutyl phthalate, also with potential toxicity on the liver antioxidant system in zebrafish, constituted the root metabolome of *M. whitei* (Jiao et al. 2020). Another metabolite that was observed in the current study was the disaccharide sugar *α*, *α*‐trehalose, which is ubiquitous in bacteria, yeast, fungi, invertebrates, as well as in lower and higher plants; it can serve as an energy source during resource‐limiting conditions (Elbein et al. 2003). It also serves as a signalling molecule that directs metabolic pathways and growth, also linked to protein and cellular membranes protection from inactivation and denaturation caused by conditions like desiccation, dehydration, heat, cold, and oxidation (Elbein et al. 2003). In the current study, this compound was found in samples obtained from all locations.

## 5 Conclusions

The plant microbiome is majorly influenced by geographic location and plant compartment. Parts of the plant microbiome were shown to be associated with specific root metabolites, which allow delimiting the different sample origins. The current study is a primer to the sustainable exploitation of plant resources and the microbial community, including metabolites. The current findings serve to expand the research frontier into deciphering the plant‐microbiome metabolome interplay of medicinal plants such as *M. whitei* . These insights are also reflective of the potential role of microbiomes in the biosynthesis of plant bioactive compounds, as reflected in the extensive literature survey. The link between root compounds and specific, mostly unclassifiable microbial taxa provides insights into the potential contribution of the microbiome in the bioprospecting for bioactive metabolites. Additionally, future studies might consider exploring potential risks, like antimicrobial resistance load likely associated with the *M. whitei* root microbiome due to the prevalence of antimicrobial metabolites.

## Author Contributions

**Expedito Olimi:** writing – original draft, investigation, conceptualization, methodology, validation, visualization, writing – review and editing, resources, supervision, project administration. **Regina Wuggenig:** writing – original draft, investigation, visualization. **Carolina Lobato:** visualization, writing – original draft. **Samuel Bickel:** writing – review and editing, visualization, writing – original draft. **Peter Kusstatscher:** writing – review and editing. **Wisnu Adi Wicaksono:** writing – review and editing, visualization. **Angelika Battisti:** investigation, writing – review and editing. **Danny Coyne:** writing – review and editing, resources. **John Adriko:** resources. **Tomislav Cernava:** writing – review and editing, writing – original draft, visualization, project administration, supervision. **Gabriele Berg:** project administration, writing – review and editing, writing – original draft, funding acquisition, conceptualization, supervision, resources.

## Conflicts of Interest

The authors declare no conflicts of interest.

## Supporting information


**Figure S1:** Alpha rarefaction for bacterial (A) and fungal (B) community at minimum sampling depths of 3000 and 5000 reads per sample for bacterial and fungal communities, respectively across the different compartments.
**Figure S2:** The microbial Shannon index and number of observed ASVs in the root endosphere and rhizosphere for the five locations. Panels (A) and (B) show bacterial and fungal richness (observed ASVs), while (C) and (D) shows the bacterial and fungal Shannon diversity indices. The boxplots represent sample distribution of samples (*n* = 8) for each of the compartment with the estimation of median, 25th, and 75th percentiles. The data points outside the box area represents outliers. Statistical tests of microbial alpha diversity differences between compartments (i.e., root and rhizosphere) in each location were conducted using Wilcoxon Signed Rank test. The legend colours correspond to different sample locations. Overall differences in alpha diversity across locations and compartments are shown above each panel.
**Figure S3:** Compartment‐specific microbial abundance of 16S rRNA genes (bacterial) and the ITS region (fungal) in the root endosphere and rhizosphere across sample locations. Panels (A) and (B) show the bacterial and fungal abundance, respectively. The boxplots represent sample distribution of samples (*n* = 8 samples) for each compartment with the estimation of median, 25th, and 75th percentiles. *p*‐values were determined using the paired‐group students' t‐test. The data points outside the boxplot area represent outliers, while legend colours represent the different sample locations.
**Figure S4:** A representation of the core microbiome, the shared and unique ASVs across locations and compartments. Panels A and B show abundance‐occupancy curves of core bacterial and fungal microbiota, based on the prevalence ranging between 25% and 100% of the samples and the minimum detection range of 0.00001%–1%. Panels C‐F are VennPlot representation of the number of unique and shared ASVs in different compartments and locations; wherein panels C and D show unique and shared bacterial and fungal ASVs in the rhizosphere, while E and F show the shared and unique ASVs in the root compartment.
**Figure S5:** Examining the microbiota which was transferred from the rhizosphere (source) and transferred into root endosphere (sink) into the root endosphere (sink) for the different locations. Panel (A) and (B) show the respective proportion of bacterial and fungal ASVs tracked from rhizosphere into the root endosphere for the different locations. The portion labelled as “Unknown” represents the unassigned part of microbiome.
**Table S1:** Pairwise differences in microbiomes for the two compartments in different locations (Adonis PERMANOVA).
**Table S2:** Shared bacterial and fungal taxa in the five locations for the two plant compartments (rhizosphere and root endosphere). Taxa are represented as families and genera (included in square brackets).

## Data Availability

The datasets generated during the current study are available on BioProject ID PRJNA1297068 (http://www.ncbi.nlm.nih.gov/bioproject/1297068).
