# Supplementary material for: Insights Into the Mondia Whitei Microbiome Across Geographic Regions in Eastern Africa
Source: Environ Microbiol Rep. 2025 Sep 27;17(5):e70200. doi: 10.1111/1758-2229.70200 (PMC12475509; doi:10.1111/1758-2229.70200)
Supplement: Supplementary file 1 — Figure S1: Alpha rarefaction for bacterial (A) and fungal (B) community at minimum sampling depths of 3000 and 5000 reads per sample for bacterial and fungal communities, respectively across the different compartments. Figure S2: The microbial Shannon index and number of observed ASVs in the root endosphere and rhizosphere for the five locations. Panels (A) and (B) show bacterial and fungal richness (observed ASVs), while (C) and (D) shows the bacterial and fungal Shannon diversity indices. The boxplots represent sample distribution of samples (n = 8) for each of the compartment with the estimation of median, 25th, and 75th percentiles. The data points outside the box area represents outliers. Statistical tests of microbial alpha diversity differences between compartments (i.e., root and rhizosphere) in each location were conducted using Wilcoxon Signed Rank test. The legend colours correspond to different sample locations. Overall differences in alpha diversity across locations and compartments are shown above each panel. Figure S3: Compartment‐specific microbial abundance of 16S rRNA genes (bacterial) and the ITS region (fungal) in the root endosphere and rhizosphere across sample locations. Panels (A) and (B) show the bacterial and fungal abundance, respectively. The boxplots represent sample distribution of samples (n = 8 samples) for each compartment with the estimation of median, 25th, and 75th percentiles. p‐values were determined using the paired‐group students' t‐test. The data points outside the boxplot area represent outliers, while legend colours represent the different sample locations. Figure S4: A representation of the core microbiome, the shared and unique ASVs across locations and compartments. Panels A and B show abundance‐occupancy curves of core bacterial and fungal microbiota, based on the prevalence ranging between 25% and 100% of the samples and the minimum detection range of 0.00001%–1%. Panels C‐F are VennPlot representation of the number of [file EMI4-17-e70200-s001.docx]

Insights into the *Mondia whitei* microbiome across geographic regions in Eastern Africa

Expedito Olimi^1,2*^, Regina Wuggenig^1^, Carolina Lobato^1^, Samuel Bickel^1^, Peter Kusstatscher^1^, Wisnu Adi Wicaksono^1^, Angelika Battisti^1^, Danny Coyne^3^, John Adriko^4,5^, Tomislav Cernava^1,2*^ and Gabriele Berg^1,6,7*^

^1^ Institute of Environmental Biotechnology, Graz University of Technology, Graz, Austria

^2^School of Biological Sciences, Faculty of Environmental and Life Sciences, University of Southampton, Southampton, United Kingdom

^3^International Institute of Tropical Agriculture (IITA), Nairobi, Kenya

^4^National Agricultural Research Organization (NARO), Kampala, Uganda

^5^National Forestry Resources Research Institute (NAFORI), Kampala, Uganda

^6^Institute for Biochemistry and Biology, University of Potsdam, Potsdam, Germany

^7^Leibniz Institute for Agricultural Engineering and Bioeconomy (ATB), Potsdam, Germany

* Corresponding author: Expedito Olimi

Email: [expedito.olimi@tugraz.at](mailto:expedito.olimi@tugraz.at)

**Supplementary Method 1: Headspace gas chromatography mass spectrometry (GC-MS) analysis**

To analyse the volatile profile of M. whitei roots, four root samples from each sampling site were processed by grinding with mortar and pestle in liquid nitrogen. Here, 200 mg of the crushed root samples were transferred into headspace vials, then closed tightly with crimp seals. The samples were left at room temperature for 30 min before measurement. An empty vial was used as a blank reference. Solid phase microextraction (SPME) coupled with GC-MS was used to determine the volatile compounds contained in M. whitei roots. For separation and detection of compounds, we used the chromatograph GC7890A with a quadrupole MS5975C (Agilent Technologies, Waldbronn, Germany) equipped with an autosampler. The SPME was conducted using a 50/30 μm divinylbenzene/carboxen/polydimethlsiloxane StableFlex fiber with a length of 2 cm. The volatile compounds in the headspace were enriched for 30 min at 35°C. The separation column was a HP-5MS column (5%- phenyl)-methylpolysiloxane, with dimensions: 30 m x 250 μm x 0.25μm (length x inner diameter x coating, 250 °C; Agilent Technologies, Waldbronn, Germany), followed by electron ionization and detection within a mass range of 35-450 Atomic mass Unit (AU). Ionization of the sample molecules took place at 70 eV and the helium flow rate was 1.2 ml/min. The GC column was kept at 40°C for 2 min before temperature was increased to 110 °C at 5 °C/min, again increased to 280 °C by 10° C/min, and finally kept at 280 °C for 3 minutes. Chromatograms were resolved for the detection of the volatile compounds. Identification of the compounds was performed with NIST MS search 2.2 included in the software-package of the NIST 2014 database. The compound suggestion with the best relative spectrum match (RMatch) from the NIST14 database was manually checked. Together with the RI-Match, the identification suggestion was accepted or deleted. The peak area of the identified compounds was considered as the semi-quantitative measure of compound concentration in the samples.

**Supplementary Method 2: High-performance liquid chromatography mass spectrometry (HPLC-MS) analysis**

Parts of the same samples that were used for GC-MS were also analysed using HPLC-MS. Briefly, 2 g of crushed root sample was used for extraction with 2 ml of methanol. The samples were treated for 15 min in an ice cooled ultrasonic bath. After this step, the solvent was transferred into a sterile 15 ml tube. The extraction process with methanol was repeated on the residual sample and the elute was added into the 15 ml tubes. The methanol evaporated using a stream of nitrogen followed by adding 1 ml of methanol to the dried residue in the 15 ml tube, which was then thoroughly mixed by vortexing for 1 minute. The samples were stored at -20 °C until the HPLC-MS analysis. Prior to starting the analysis, the samples were thawed at room temperature, centrifuged at 16,000g for 20 mins, and the supernatant was transferred into sterile 1.5 ml Eppendorf tubes. The samples were analysed using a HPLC hybrid quadrupole Orbitrap mass spectrometer – HPLC Ultimate 3000 (Q Exactive; Thermo Scientific, Bremen, Germany). The column Waters Atlantis dC18 (Phenomenex, Aschaffenburg, Germany) with dimensions (3 μM, 2.1 mm x 100 mm) was used to separate the components. The solvents which were used were: formic acid (0.1 %, v/v) in acetonitrile (A), and aqueous formic acid (0.1 %, v/v). Gradient elution conditions were 10 % A to 80 % A in 35 min, with starting column conditions (25°C, 5 min). The eluent flow was maintained at 0.3 ml/min with the column maintained at 25°C. Sample analysis was performed with negative ion electrospray ionization detection and positive ion detection. The electrospray ionization conditions were 350 °C capillary temperature at 3.2 kV spray voltage. The scans for metabolites were in the range of 100.0 to 1500.0 m/z with an automatic gain control target set to 10^6^ with a maximum accumulation time of 200 ms, and a resolution of 70,000 FWHM (Full Width at Half Maximum). Full MS-SIM (Mass Spectrometry-based Single Ion Monitoring) and targeted MS2 cycles were alternately used (i.e. full MS/dd-MS2 mode as the strategy for obtaining mass spectrometry data). The data were analysed using Compound Discoverer 3.2 (Thermo Scientific). Briefly, the program performed retention time alignment, compound detection and grouping, predicted elemental compositions for all compounds, gap filling, and background chemical correction using blank samples (Methanol). The compounds were identified using the mzCloud (ddMS2) and ChemSpider (formula or exact mass) databases.


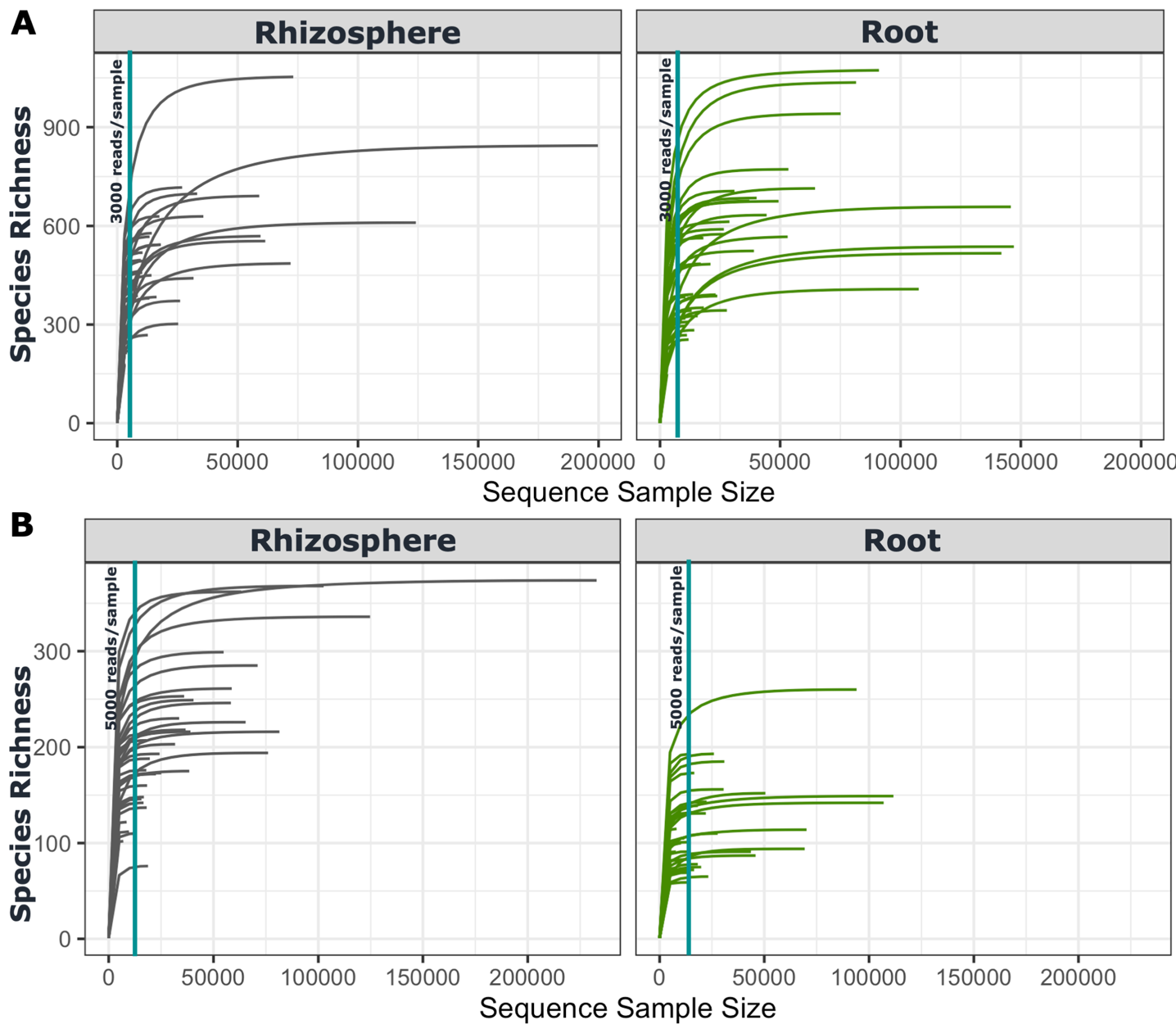


**Supplementary Figure 1.** Alpha rarefaction for bacterial (A) and fungal (B) community at minimum sampling depths of 3000 and 5000 reads per sample for bacterial and fungal communities, respectively across the different compartments.


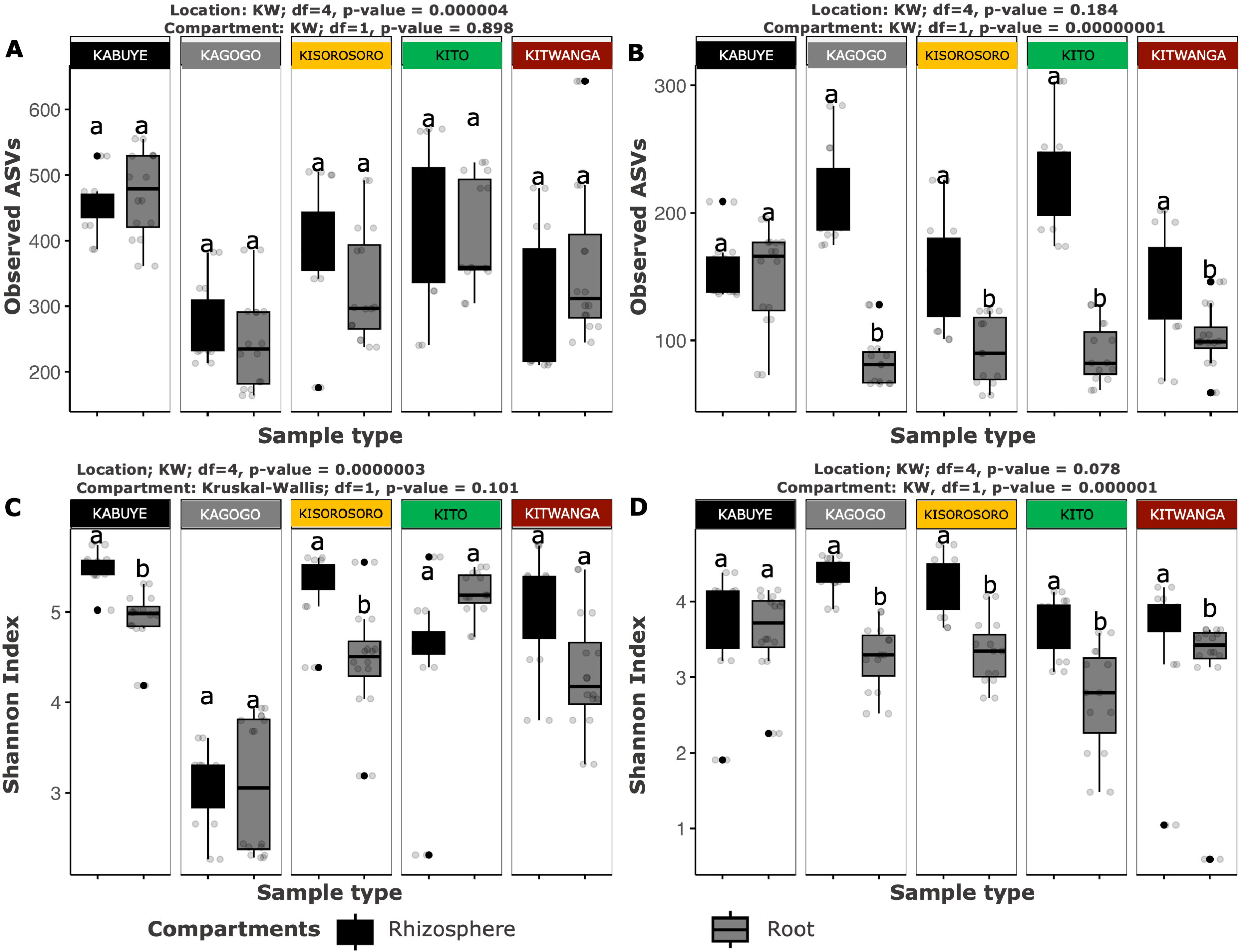


**Supplementary Figure 2.** The microbial Shannon index and number of observed ASVs in the root endosphere and rhizosphere for the five locations. Panels (A) and (B) show bacterial and fungal richness (observed ASVs), while (C) and (D) shows the bacterial and fungal Shannon diversity indices. The boxplots represent sample distribution of samples (n=8) for each of the compartment with the estimation of median, 25^th^, and 75^th^ percentiles. The data points outside the box area represents outliers. Statistical tests of microbial alpha diversity differences between compartments (i.e., root and rhizosphere) in each location were conducted using Wilcoxon Signed Rank test. The legend colours correspond to different sample locations. Overall differences in alpha diversity across locations and compartments are shown above each panel.


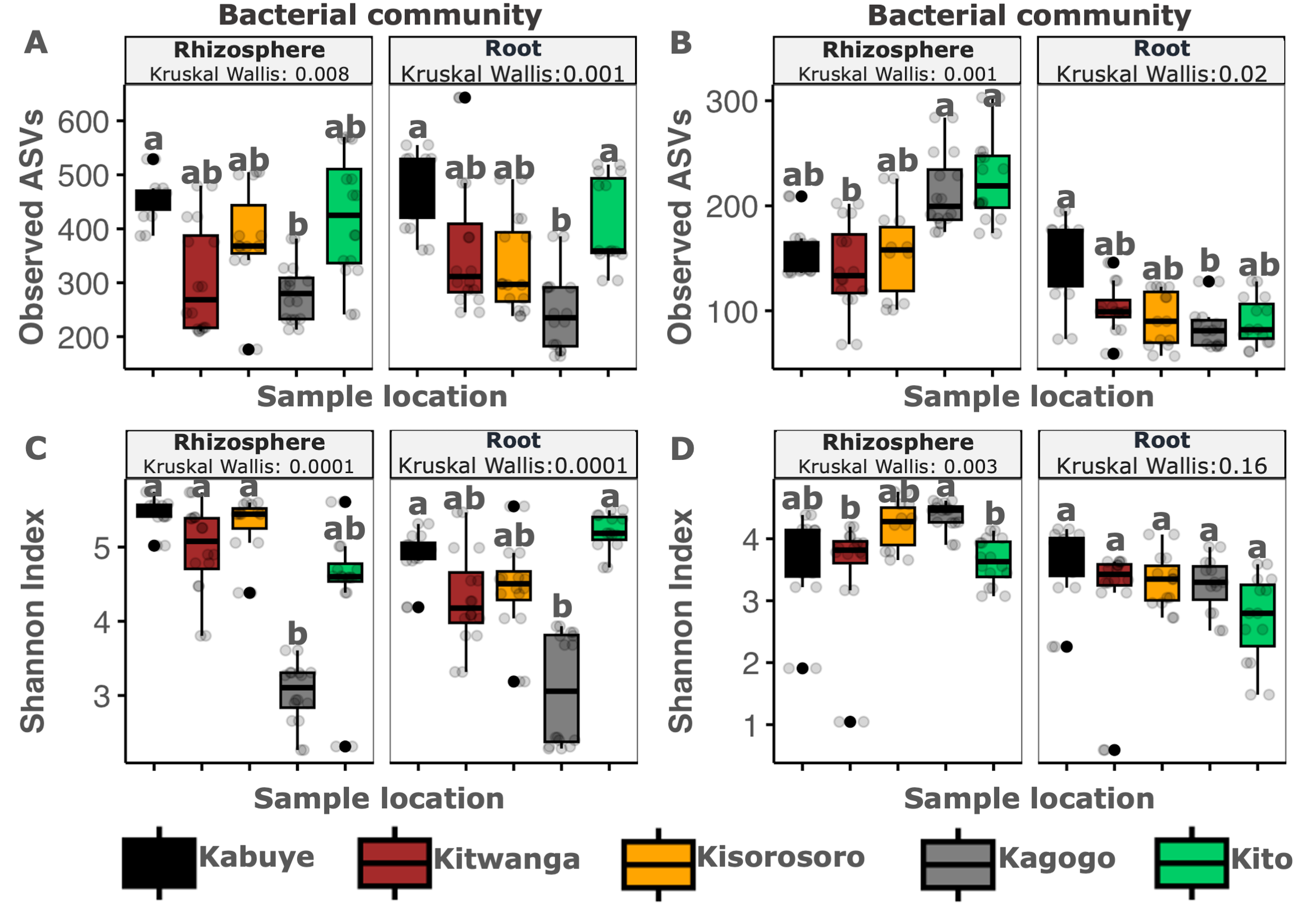


**Supplementary Figure 3.** Compartment-specific microbial abundance of 16S rRNA genes (bacterial) and the ITS region (fungal) in the root endosphere and rhizosphere across sample locations. Panels (A) and (B) show the bacterial and fungal abundance, respectively. The boxplots represent sample distribution of samples (n=8 samples) for each compartment with the estimation of median, 25^th^, and 75^th^ percentiles. p-values were determined using the paired-group students’ t-test. The data points outside the boxplot area represent outliers, while legend colours represent the different sample locations.


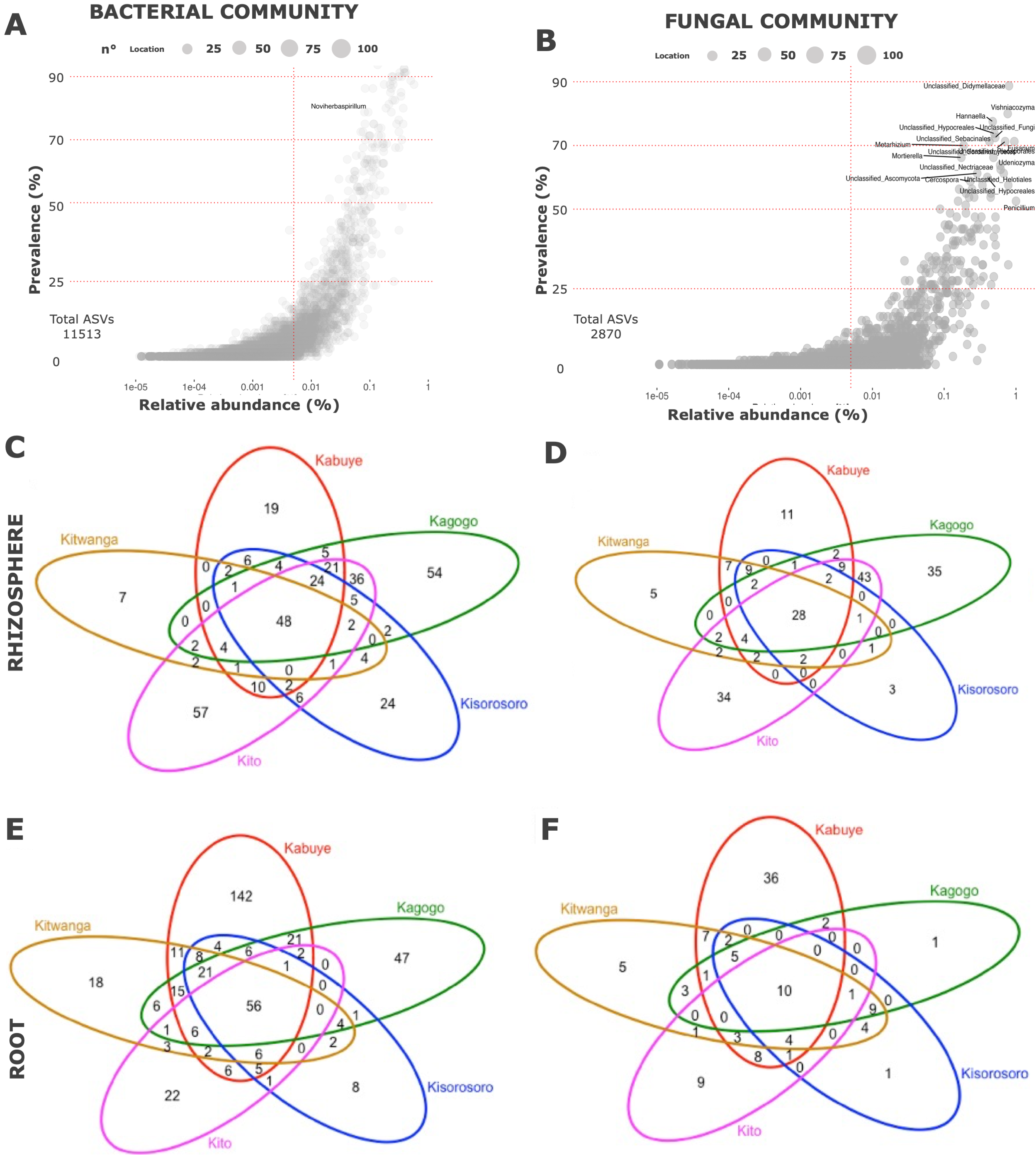


**Supplementary Figure 4.** A representation of the core microbiome, the shared and unique ASVs across locations and compartments. Panels A and B show abundance-occupancy curves of core bacterial and fungal microbiota, based on the prevalence ranging between 25% to 100% of the samples and the minimum detection range of 0.00001% to 1%.Panels C-F are VennPlot representation of the number of unique and shared ASVs in different compartments and locations; wherein panels C and D show unique and shared bacterial and fungal ASVs in the rhizosphere, while E and F show the shared and unique ASVs in the root compartment.


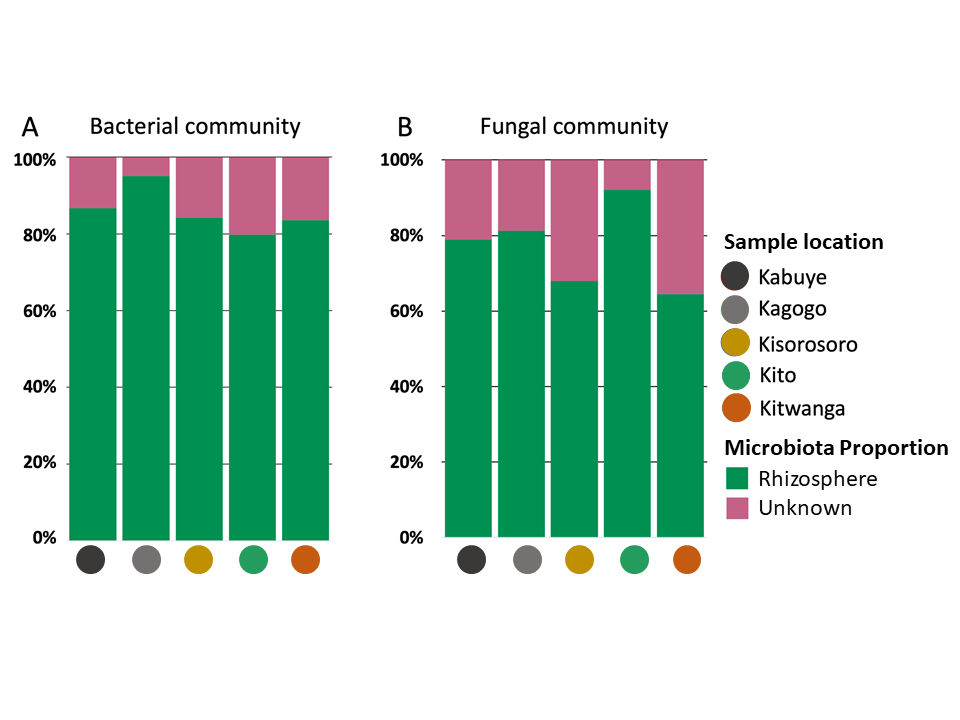


**Supplementary Figure 5.** Examining the microbiota which was transferred from the rhizosphere (source) and transferred into root endosphere (sink) into the root endosphere (sink) for the different locations. Panel (A) and (B) show the respective proportion of bacterial and fungal ASVs tracked from rhizosphere into the root endosphere for the different locations. The portion labelled as “Unknown” represents the unassigned part of microbiome.

**Table S1.** Pairwise differences in microbiomes for the two compartments in different locations (Adonis PERMANOVA)

|  | Bacterial community | | | | | | | | | | | | Fungal community | | | | | | | | | | | |
| --- | --- | --- | --- | --- | --- | --- | --- | --- | --- | --- | --- | --- | --- | --- | --- | --- | --- | --- | --- | --- | --- | --- | --- | --- |
|  | Root endosphere | | | | | | Rhizosphere | | | | | | Root endosphere | | | | | | Rhizosphere | | | | | |
| PERMANOVA (General) | df=4, R^2^=25%, P=0.001 | | | | | | df=4, R^2^=22%, P=0.001 | | | | | | df=4, R^2^=29%, P=0.001 | | | | | | df=4, R^2^=31%, P=0.001 | | | | | |
|  | Df | Sum of Sqs | R2 | F | Pr(>F) | Sig | Df | Sum of Sqs | R2 | F | Pr(>F) | Sig | Df | Sum of Sqs | R2 | F | Pr(>F) | Sig | Df | SumOfSqs | R2 | F | Pr(>F) | Sig |
| Kabuye vs Kitwanga | 1 | 0.528 | 0.172 | 2.916 | 0.002 | ** | 1 | 0.454 | 0.117 | 1.857 | 0.010 | ** | 1 | 0.776 | 0.243 | 4.497 | 0.001 | *** | 1 | 0.270 | 0.092 | 1.433 | 0.004 | ** |
| Kabuye vs Kisorosoro | 1 | 0.579 | 0.189 | 3.271 | 0.001 | *** | 1 | 0.381 | 0.110 | 1.735 | 0.003 | ** | 1 | 0.839 | 0.244 | 4.519 | 0.001 | *** | 1 | 0.384 | 0.115 | 1.818 | 0.001 | *** |
| Kabuye vs Kagogo | 1 | 0.635 | 0.235 | 4.310 | 0.002 | ** | 1 | 0.493 | 0.162 | 2.699 | 0.001 | *** | 1 | 0.953 | 0.252 | 4.717 | 0.001 | *** | 1 | 0.895 | 0.2936 | 5.819 | 0.001 | *** |
| Kabuye vs Kito | 1 | 0.789 | 0.218 | 3.903 | 0.001 | *** | 1 | 0.563 | 0.173 | 2.920 | 0.001 | *** | 1 | 0.716 | 0.216 | 3.855 | 0.001 | *** | 1 | 0.776 | 0.2599 | 4.917 | 0.001 | *** |
| Kitwanga vs Kisorosoro | 1 | 0.304 | 0.095 | 1.474 | 0.002 | ** | 1 | 0.380 | 0.099 | 1.541 | 0.003 | ** | 1 | 0.232 | 0.082 | 1.254 | 0.033 | * | 1 | 0.325 | 0.0944 | 1.458 | 0.008 | ** |
| Kitwanga vs Kagogo | 1 | 0.463 | 0.158 | 2.621 | 0.002 | ** | 1 | 0.713 | 0.196 | 3.408 | 0.001 | *** | 1 | 0.359 | 0.113 | 1.782 | 0.001 | *** | 1 | 1.048 | 0.3109 | 6.314 | 0.001 | *** |
| Kitwanga vs Kito | 1 | 0.480 | 0.129 | 2.074 | 0.001 | *** | 1 | 0.619 | 0.168 | 2.819 | 0.001 | *** | 1 | 0.881 | 0.253 | 4.750 | 0.002 | ** | 1 | 0.807 | 0.2533 | 4.748 | 0.001 | *** |
| Kisorosoro vs Kagogo | 1 | 0.543 | 0.183 | 3.146 | 0.001 | *** | 1 | 0.620 | 0.193 | 3.356 | 0.002 | ** | 1 | 0.317 | 0.096 | 1.479 | 0.002 | ** | 1 | 1.127 | 0.299 | 5.972 | 0.001 | *** |
| Kisorosoro vs Kito | 1 | 0.537 | 0.144 | 2.363 | 0.001 | *** | 1 | 0.562 | 0.171 | 2.884 | 0.001 | *** | 1 | 0.872 | 0.239 | 4.395 | 0.001 | *** | 1 | 0.981 | 0.2665 | 5.087 | 0.001 | *** |
| Kagogo vs Kito | 1 | 0.879 | 0.241 | 4.443 | 0.001 | *** | 1 | 0.381 | 0.147 | 2.415 | 0.001 | *** | 1 | 1.152 | 0.277 | 5.366 | 0.001 | *** | 1 | 0.476 | 0.201 | 3.521 | 0.001 | *** |

**Table S2.** Shared bacterial and fungal taxa in the five locations for the two plant compartments (rhizosphere and root endosphere). Taxa are represented as families and genera (included in square brackets).

| Kabuye, Kagogo, Kisorosoro, Kito & Kitwanga | Bacterial community | | Fungal community | |
| --- | --- | --- | --- | --- |
|  | Rhizosphere | Root endosphere | Rhizosphere | Root endosphere |
|  | Unclassified Gaiellales [Unclassified Gaiellales] | Pseudomonadaceae [Pseudomonas] | Didymellaceae [Unclassified Didymellaceae] | Unclassified Basidiomycota [Unclassified Basidiomycota] |
|  | Pseudonocardiaceae [Actinophytocola] | Pseudonocardiaceae [Actinophytocola] | Didymellaceae [Stagonosporopsis] | Unclassified Xylariales [Unclassified Xylariales] |
|  | Pirellulaceae [Unclassified Pirellulaceae] | Pirellulaceae [Unclassified Pirellulaceae] | Unclassified Basidiomycota [Unclassified Basidiomycota] | Unclassified Ascomycota [Unclassified Ascomycota] |
|  | Pseudomonadaceae [Pseudomonas] | Xanthobacteraceae [Unclassified Xanthobacteraceae] | Bulleribasidiaceae [Vishniacozyma] | Unclassified Eurotiomycetes [Unclassified Eurotiomycetes] |
|  | Devosiaceae [Devosia] | Devosiaceae [Devosia] | Unclassified Pleosporales [Unclassified Pleosporales] | Nectriaceae [Fusarium] |
|  | Xanthobacteraceae [Unclassified Xanthobacteraceae] | Mycobacteriaceae [Mycobacterium] | Bulleribasidiaceae [Hannaella] | Unclassified Hypocreales [Unclassified Hypocreales] |
|  | Mycobacteriaceae [Mycobacterium] | Sphingomonadaceae [Sphingomonas] | Unclassified Xylariales [Unclassified Xylariales] | Unclassified Auriculariales [Unclassified Auriculariales] |
|  | Bacillaceae [Bacillus] | Rhodomicrobiaceae [Rhodomicrobium] | Unclassified Sebacinales [Unclassified Sebacinales] |  |
|  | Sphingomonadaceae [Sphingomonas] | Micromonosporaceae [Virgisporangium] | Unclassified Auriculariales [Unclassified Auriculariales] |  |
|  | Streptomycetaceae [Streptomyces] | Oxalobacteraceae [Noviherbaspirillum] | Unclassified Ascomycota [Unclassified Ascomycota] |  |
|  | Nitrospiraceae [Nitrospira] | Dongiaceae [Dongia] | Unclassified Fungi [Unclassified Fungi] |  |
|  | Caulobacteraceae [Phenylobacterium] | Sandaracinaceae [Unclassified Sandaracinaceae] | Unclassified Sordariomycetes [Unclassified Sordariomycetes] |  |
|  | Rokubacteriales [Rokubacteriales] | Unclassified Microtrichales [Unclassified Microtrichales] | Unclassified Hypocreales [Unclassified Hypocreales] |  |
|  | Chthoniobacteraceae [Candidatus Udaeobacter] | SC-I-84 [SC-I-84] | Unclassified Eurotiomycetes [Unclassified Eurotiomycetes] |  |
|  | Vicinamibacteraceae [Vicinamibacteraceae] | Rhizobiaceae [Allorhizobium-Neorhizobium-Pararhizobium-Rhizobium] | Bionectriaceae [Unclassified Bionectriaceae] |  |
|  | IMCC26256 [IMCC26256] | Caulobacteraceae [Phenylobacterium] | Mycosphaerellaceae [Cercospora] |  |
|  | Sphingomonadaceae [Sphingobium] | Unclassified Gammaproteobacteria Incertae Sedis [Acidibacter] | Ophiocordycipitaceae [Purpureocillium] |  |
|  | Solirubrobacteraceae [Solirubrobacter] | Bacillaceae [Bacillus] | Mortierellaceae [Mortierella] |  |
|  | Rhizobiaceae [Allorhizobium-Neorhizobium-Pararhizobium-Rhizobium] | Chthoniobacteraceae [Candidatus Udaeobacter] | Nectriaceae [Fusarium] |  |
|  | Unclassified Alphaproteobacteria [Unclassified Alphaproteobacteria] | Rhizobiaceae [Unclassified Rhizobiaceae] | Clavicipitaceae [Metarhizium] |  |
|  | CCD24 [CCD24] | Xanthobacteraceae [Bradyrhizobium] | Unclassified Saccharomycetales [Unclassified Saccharomycetales] |  |
|  | Pseudonocardiaceae [Kibdelosporangium] | Enterobacteriaceae [Enterobacter] | Nectriaceae [Unclassified Nectriaceae] |  |
|  | Methyloligellaceae [Unclassified Methyloligellaceae] | IMCC26256 [IMCC26256] |  |  |
|  | Nocardiaceae [Nocardia] | Sphingomonadaceae [Sphingobium] |  |  |
|  | KD4-96 [KD4-96] | Solirubrobacteraceae [Solirubrobacter] |  |  |
|  | Erwiniaceae [Pantoea] | Rhizobiaceae [Mesorhizobium] |  |  |
|  | Nocardioidaceae [Kribbella] | CCD24 [CCD24] |  |  |
|  | Rhizobiales Incertae Sedis [Nordella] | Pseudonocardiaceae [Kibdelosporangium] |  |  |
|  | Unclassified Gammaproteobacteria Incertae Sedis [Acidibacter] | Reyranellaceae [Reyranella] |  |  |
|  | Xanthobacteraceae [Bradyrhizobium] | Methyloligellaceae [Unclassified Methyloligellaceae] |  |  |
|  | PLTA13 [PLTA13] | Nocardiaceae [Nocardia] |  |  |
|  | Steroidobacteraceae [Unclassified Steroidobacteraceae] | KD4-96 [KD4-96] |  |  |
|  | Dongiaceae [Dongia] | Rhizobiales Incertae Sedis [Nordella] |  |  |
|  | Burkholderiaceae [Ralstonia] | Steroidobacteraceae [Unclassified Steroidobacteraceae] |  |  |
|  |  | Burkholderiaceae [Ralstonia] |  |  |

**Table S3.** Functions of compounds identified by GC/LC-MS analysis, including details of the regions where samples were obtained; with Kabuye represented by (A), Kagogo (B), Kisorosoro (C), Kito (D), and Kitwanga (E). The shades of grey show the location whose root samples showed presence of the metabolite.

| Compound | Region | | | | | Potential role | Functional category |
| --- | --- | --- | --- | --- | --- | --- | --- |
|  | A | B | C | D | E |  |  |
| 1-octen-3-one |  |  |  |  |  | Found in Agaricus bisporus (Grove, 1981) and Scytinostroma portentosum (Rühl et al., 2018). It is a characteristic mushroom flavour (Rühl et al., 2018; Hausch et al., 2020). An oxylipin, product of the oxidation and cleavage of linoleic acid (Combet et al., 2006; Hausch et al., 2020). It is also found in Perilla frutescens leaves (Seo and Baek, 2009), Persea americana (Hausch et al., 2020) and Teucrium manghuaense (Yin et al., 2009) with potential DPPH(-), hydroxyl radical scavenging and anti-tumour activity. Is a biomarker for B. cinerea in strawberry (Vandendriessche et al., 2012) that acts as a phytotoxin by inhibiting germination in A. thaliana (Hung et al., 2014; S. Lee et al., 2014). Shows strong antibacterial activity (Xiong et al., 2017) and has a role in resistance of subclover cotyledon to the red-legged earth mite (Jiang et al., 1996). | -Antimicrobial  -Antitumour |
| 1-penten-3-one |  |  |  |  |  | Present in Solanum sp. (Ursem et al., 2008; Li, Fu, et al., 2020), rapeseed oil (Jeleń et al., 2007), Medicago sativa flowers (Tava and Pecetti, 1997), red clover pods (Buttery et al., 1984) and Kiwi (Zhao et al., 2021). |  |
| 1-propanol |  |  |  |  |  | Present in Coffea arabica flowers (Stashenko et al., 2013). Can be produced by Shimwellia blattae from 1,2-propanediol (Urano et al., 2015). It can boost antibiotic production in different bacteria (Potvin and Péringer, 1993; Li et al., 2014) as well as production of odd-chain fatty acids(Zhang et al., 2019). | -Microbe-microbe interactions  -Anti-microbial |
| 1,8-nonadiyne |  |  |  |  |  | A volatile from Nodulisporium sp. (Carballeira et al., 2006). Precursor of potent antifungal agents (Carballeira et al., 2006). | -Antifungal activity |
| β-maaliene) |  |  |  |  |  | Can be found in the roots (Goel et al., 2007; Huong et al., 2015) and rhizomes (Tanaka and Komatsu, 2007; Peng et al., 2011) of various plant systems. It is also found in Croton eluteria (Hagedorn and Brown, 1991), Achillea cretica (Küçükbay et al., 2012), Ephedra nebrodensis (Maggi et al., 2011), Nardostachys chinensis (J. Wang, Zhao, et al., 2010) and Annona sylvatica (Formagio et al., 2013). Has potential anti-inflammatory and antimicrobial activities (Barros et al., 2013), and an allergen inhibitor role (Lin et al., 2022). | Anti-inflammatory and antimicrobial |
| 3,5,5-trimethyl-  2-cyclopenten-1-one |  |  |  |  |  | Found associated with rice (Oryza sp.) (Cho et al., 2014) |  |
| 2-decenal, (E)- |  |  |  |  |  | Found in different fruit juices (Anjou et al., 1967; Servili, 2000; Pino et al., 2005) , but also in Cynomorium songaricum stems (Zhou et al., 2009) and celery roots (Sipailiene et al., 2005). It is a chemical defence secretion of Hotea gambiae (Gough et al., 1985) and an alarm pheromone in Bathycoelia Distincta (Pal et al., 2022). It has potential nematicidal properties (Caboni et al., 2012; Ntalli et al., 2016). In wheat it can be used as a biocontrol of storage pests (Ntalli et al., 2016). | -Nematicidal  -Pheromones |
| 2-ethyl-1-dodecanol |  |  |  |  |  | Present in Oryza sativa (Goufo et al., 2010) and Vigna radiata (Attar et al., 2017). |  |
| 2-heptenal |  |  |  |  |  | Major lipidic volatile in Aspleniaceae (Froissard et al., 2015). Found in rapeseed oil (Jeleń et al., 2007) and grape seed oil (Kiralan et al., 2018). |  |
| 2-ethyl-2-hexen-1-ol |  |  |  |  |  | Found in melon (Chen et al., 2016). It is the major compound in the Camponotus quadrisectus mandibular glands and provides antibiotic activity (Voegtle et al., 2008). | -Antibiotic activity |
| 2-ethyl-2-hexenal |  |  |  |  |  | It is the major compound in the Camponotus quadrisectus mandibular glands and provides antibiotic activity (Voegtle et al., 2008). |  |
| 2-hexyl-1-octanol |  |  |  |  |  | Extracts from Barleria lupulina (Sarmad et al., 2012) and Succisa pratensis leaves (Witkowska-Banaszczak and Długaszewska, 2017) exhibited antimicrobial and antioxidant activities. | -Antimicrobial  -Antioxidant |
| 2-nonen-1-ol, (E) |  |  |  |  |  | It is a versatile compound found in sunflower (Turgumbayeva et al., 2018), soybean seeds (Ghahari et al., 2017), tomato (Cortina et al., 2016), watermelon (Lim, 2012), mung bean (Attar et al., 2017) and marine macroalgae. In Gallus gallus eggs it was a signature metabolite for loss of freshness, possibly by bacterial degradation (Cumeras et al., 2016). |  |
| 2-nonenal, (E)- |  |  |  |  |  | It is present in Zingiber Officinale (Sharma et al., 2016), Rhodiola rosea (Rohloff, 2002) rhizomes and carrot root with insecticidal effects (Guerin and Ryan, 1980; Chamberlain et al., 1991). But also in celery (Kurobayashi et al., 2006), mango (Pino et al., 2005) and strawberry flowers (Blažytė-Čereškienė et al., 2019). It can attract both mutualistic insects (Blažytė-Čereškienė et al., 2019) and predators (Karmakar et al., 2020), and inhibit seed germination in soybean (Gardner et al., 1990). It is a fungicide against R. solani, S. rolfsii (Vaughn and Gardner, 1993), B. cinerea and A. alternata (Hamilton-Kemp et al., 1992). It is able to enhance the antagonistic effect of Pseudomonas and Bacillus species against B. cinerea (Abanda-Nkpwatt et al., 2006). It is a sex pheromone in Anomala albopilosa (Leal et al., 1996). | -Sex pheromone  -Microbe-microbe interactions |
| 2-octenal, (E) |  |  |  |  |  | It is a product of 2,4-Decadienal autooxidation (Matthews et al., 1971), and a volatile in many plant species (Pino et al., 2005; Battinelli et al., 2006; Miyazawa and Kawata, 2006; Stashenko et al., 2013; Cortina et al., 2016). It is known for its antifungal activities (Pauli, 2001; Battinelli et al., 2006; Ulrich et al., 2015; Liarzi et al., 2020), but it also displays acaricidal activity (Song and Lee, 2018; Park et al., 2021). It is used as a signalling molecule and a defence mechanism in various insects (Gunawardena and Bandumathie, 1993; Harraca et al., 2010; Noge et al., 2012). | -Antifungal activity |
| 2-pentenal, (E)- |  |  |  |  |  | Present in Medicago sativa (Tava and Pecetti, 1997), and Coffea arabica (Stashenko et al., 2013) flowers and other fruits (Kallio and Linko, 1973; Pino et al., 2005; Xu and Barringer, 2010; Wang et al., 2019). Highly active against Mycobacterium bovis. | -Antimicrobial |
| 2,4-dimethyl-1-heptene |  |  |  |  |  | Present in early stages of Jasminum sambac flowers (Ghissing et al., 2022), in Citrus grandis fruits (Chung et al., 2012). in the roots of Gentiana lutea (Mustafa et al., 2016), and in Enteromorpha linza (Sukatar et al., 2006). |  |
| 2,4-nonadienal |  |  |  |  |  | Detected in Agaricus bisporus (Grove, 1981), tomato (Tang et al., 2022), Citrus reticulata (Buettner et al., 2003), Coffea arabica flowers (Stashenko et al., 2013) and in Fushimi sweet pepper, showing bio antimutagenicity properties (Nakamura et al., 1999). In cereals it acts as a repellent of Sitophilus granaries (Germinara et al., 2008, 2015). | -Antimutagenic activity |
| 2,6-dimethyldecane |  |  |  |  |  | Produced by Chaetomium globosum, with potential antioxidant effects and antagonistic properties against pathogenic fungi (Kumari et al., 2022). | -Antioxidant and antifungal activity |
| 2,6-nonadienal, (E,E)- |  |  |  |  |  | A major volatile in cucumber for fresh aroma (Kemp et al., 1974; Buescher and Buescher, 2001). It has insect attractive properties (Karmakar et al., 2020; Yuan et al., 2022) and bactericidal activity (Cho et al., 2004; Sotiroudis et al., 2010). It is also a volatile in marine macroalgae (Güven et al., 2013). | -Bactericidal activity |
| 3-decyn-2-ol |  |  |  |  |  | It is a compound of cotton (Zeringue and McCormick, 1989) and kiwi (Zhao et al., 2006) leaves. It is also found in the pericarp of Zanthoxylum bungeanum (L. Wang et al., 2010). A key compound for the antagonistic activity of Chlorella vulgaris against Fusarium oxysporum (Perveen et al., 2022). | -Microbe-microbe interactions |
| 3-ethyl-4-methylpentan-1-ol |  |  |  |  |  | Associated with the fresh aroma of Torenia fournieri, and increases when cultivated in bio-compost containing greater abundance of microbial genera related to enhanced uptake of soil nutrients and environmental defence (Morais et al., 2022). It is also present in Ligusticum chuanxiong rhizome and Cynanchum stauntonii roots with possible antiviral effects (Zai-Chang et al., 2005). | -Antiviral activity |
| 4,4-dimethyl-2-  cyclopenten-1-one |  |  |  |  |  | Occasionally found in Perilla frutescens (Tian et al., 2014). It displays high tumour-specific cytotoxicity (Nakayachi et al., 2004). | -Anti-tumour |
| 6-methyl-5-Hepten-2-one |  |  |  |  |  | Present in flowers extracts of different plant species (Sashidhara et al., 2006; Majetic et al., 2007; Jabalpurwala et al., 2009; Stashenko et al., 2013). It is also detected in other compartments of many plants (Linko et al., 1978; Padmaja et al., 2010; Kim and Park, 2012). Is involved in the rice yellowing process (Liu et al., 2021). It has antifungal capabilities against Aspergillus sp. (Kim and Park, 2012) and can act as a signalling molecule emitted in potato upon attack (Laznik and Trdan, 2016). | -Antifungal |
| 6-methyl-3,  5-heptadiene-2-one |  |  |  |  |  | Has shown to be effective in modulating innate immune responses in humans, possibly by an inhibition of neutrophil migration and ROS production (Schepetkin et al., 2015). It is found in Curcuma manga (Ahmad and Misra, 1994; Wahab et al., 2011; Schepetkin et al., 2015). | -Immunomodulatory potential |
| 7-hexadecenal, (Z) |  |  |  |  |  | Found in Cinnamomum cassia (Deng et al., 2014). |  |
| benzaldehyde |  |  |  |  |  | Benzaldehyde is an aromatic aldehyde used in cosmetics as a denaturant, a flavouring agent, and as a fragrance (Andersen, 2006), obtained by [oxidation](https://www.sciencedirect.com/topics/biochemistry-genetics-and-molecular-biology/alpha-oxidation) of toluene (Das and Banik, 2021). It has carcinostatic properties (Takeuchi et al., 1978; Pettersen et al., 1983; MacEwen, 1986). It is a metabolite found in Prunus avium (Wen et al., 2014), and also produced by Pseudomonas putida; however it can be also toxic towards it (Simmonds and Robinson, 1998). It also has antiviral activities (Tolstorozhev et al., 2012). | -Antitumour and antiviral activity |
| 2-hydroxy-4-methoxy-  benzaldehyde |  |  |  |  |  | Is the main volatile component in M. whitei with vanilla aroma (Aremu et al., 2011) and potent tyrosinase inhibitory properties (Kubo and Kinst-Hori, 1999). Also found in Hemidesmus indicus (Sircar et al., 2007; Srikanta et al., 2011) , Periploca sepium and Dendrocalamus hamiltonii roots with insecticidal activity (George et al., 1999; Chu et al., 2012) as well as antimicrobial activities against fungi and bacteria (J. Wang, Liu, et al., 2010), including methicillin-resistant Staphylococcus aureus (Andati et al., 2021) and H. pylori (Srikanta et al., 2011). It also has moderate antioxidant activity (J. Wang, Liu, et al., 2010) and ovicidal activity against Anopheles Gambiae (Andati et al., 2021). | -Antimicrobial and insecticidal activity |
| 2,4-dihydroxy-  6-methyl-benzaldehyde  (o-Orsellinaldehyde) |  |  |  |  |  | Found in Parmotrema sp. (Shiromi et al., 2021) and Grifola fondosa with selective cytotoxic effect against Hep3B cells (Lin and Liu, 2006) and anti-inflammatory and immunomodulatory effects (Tomas-Hernandez et al., 2021). | Anti-inflammatory and immunomodulatory effects. |
| , 3-hydroxy-4-  methoxy-Benzaldehyde  (Isovanillin) |  |  |  |  |  | Isovanillin has been reported in many plant systems (Chang et al., 2000; Cioffi et al., 2002; P.-C. Wang et al., 2013; Zhang et al., 2013; Sadraei et al., 2014; Akter et al., 2016; Lin et al., 2016), including M. whitei (Koorbanally et al., 2000), with potential antioxidant (Cioffi et al., 2002; Akter et al., 2016), antidiarrheal and antispasmodic (Sadraei et al., 2014) activities. | -Anti-oxidant |
| 4-hydroxy-benzaldehyde |  |  |  |  |  | It is a vanillin precursor (Podstolski et al., 2002) found in many plant species (Chang and Lee, 1998; Podstolski et al., 2002; Hsieh et al., 2005; Schindler et al., 2005; Sircar and Mitra, 2008; Chamarthi et al., 2012; Aziz et al., 2013; Manaharan et al., 2013) with antidiabetic potential (Manaharan et al., 2013). It is a lignocellulose-derived by-product, inhibitor of microbial growth during fermentation processes (Zhang and Ezeji, 2014; Kurosawa et al., 2015; Yi et al., 2015). Is a product of Pycnoporus cinnabarinus p-coumaric acid conversion (Lomascolo et al., 2001) and affects antibiotic susceptibility in Acinetobacter baumannii (Shin et al., 2018). It is also a defensive secretion in Acilius sp. (Newhart and Mumma, 1979). | -Microbe-microbe interactions |
| 4-methoxy-benzaldehyde  (p-Anisaldehyde) |  |  |  |  |  | Phenolic compound found in Illicium verum (A. Y. Lee et al., 2014), Vanilla pompona (Ehlers and Pfister, 1997), Piper philippinum (Chen et al., 2007), Tabebuia avellanedae (Wagner et al., 1989), Tagetes lucida (Bicchi et al., 1997), Pimpinella saxifraga (Ksouda et al., 2019), Foeniculum vulgare (Sharopov et al., 2017). It is also a major compound in Pleurotus sp., with a contribution towards the production of hydrogen peroxide (Gutiérrez et al., 1994; Okamoto et al., 2002). Additionally, plant-derived aldehydes possess broad-spectrum antimicrobial activity without promoting resistance (Okamoto et al., 2002; Fitzgerald et al., 2005; Ksouda et al., 2019; Adewunmi et al., 2020). It shows DPPH radical scavenging potential (191, 192). It has a role as a male pheromone in Chrysotropia ciliata (Thöming et al., 2020) and as insect repellent (Showler and Harlien, 2018). | -Antimicrobial and Insect repellent |
| β-myrcene |  |  |  |  |  | An aromatic volatile used in the food and detergent industry (Hwang et al., 2017), with antioxidant (91–93)(Messaoud et al., 2012; Xanthis et al., 2021) and anti-metastatic (Lee et al., 2015) properties. It is broadly found across plants species (Jabalpurwala et al., 2009; Singh et al., 2009; Martini et al., 2010; Hassanpouraghdam, 2011; Messaoud et al., 2012; Padalia et al., 2012; Stashenko et al., 2013; Bedini et al., 2015; Lee et al., 2015; Chen et al., 2021; Xanthis et al., 2021), but also insects for pheromone production (Byers, 1982). It has insect repellent activity (Bedini et al., 2015) and can enhance Encarsia formosa efficacy as a biological control against Bemisia tabaci (Chen et al., 2021). It has been shown to be responsible for phytotoxicity in some plants (Singh et al., 2009; Hsiung et al., 2013) via oxidative damage, and fungi (Zamponi et al., 2006). Microbial synthesis of myrcene has not yet been reported, however it is the starting material for high-value compounds, such as geraniol, linalool and menthol (Kim et al., 2015) and can be transformed using bacteria like Rhodococcus erythropolis (91–93)(Thompson et al., 2010) and P. aeruginosa (Esmaeili and Hashemi, 2011). | -Insect repellent |
| 3-methyl-butanal |  |  |  |  |  | Found in Coffea sp. (Semmelroch and Grosch, 1996), Solanum lypercosum (Cortina et al., 2016) and Punica granatum seeds (Güler and Gül, 2017). |  |
| 4-methyl-,  cis-cyclohexanemethanol |  |  |  |  |  | Cyclohexanemethanol, 4-methyl-, cis- and metabolites thereof have toxic properties (Lan et al., 2015). Used for cleaning and processing coal, it can induce growth arrest in yeast [(Ayers et al., 2020)](https://www.zotero.org/google-docs/?tq17fY). | -Antimicrobial activity |
| decane |  |  |  |  |  | It is a plant metabolite found in Carica papaya (Flath and Forrey, 1977), Cistus creticus (Paolini et al., 2009), Malus silvestris flowers (Buchbauer et al., 1993), Allium porrum leaves (Noleau et al., 1991), Hypericum sp. (Saroglou et al., 2007) and Eucalyptus grandis (Zhiqun et al., 2017), where it provided repellence towards E. fetida. | -Insect repellent |
| 2-pentyl-furan |  |  |  |  |  | It is a metabolite found in thermal processed food and juices (Frank et al., 2020) but it is also a plant metabolite reported in many species (Tomczykowa et al., 2011; Xu et al., 2011; Cho et al., 2012) that can be produced upon fungal (Forlani et al., 2011) or insect infection (Lawo et al., 2011). It has a role as an Aspergillus metabolite (Chambers et al., 2011) but it can also have antifungal properties (Tomczykowa et al., 2011) and growth promoting effects (Zou et al., 2010) in plants. | -Antifungal properties |
| D germacrene |  |  |  |  |  | It is a potential biodefense against malaria and dengue vectors (Oliveira et al., 2022) and an anti-cancer agent (Silva et al., 2013). It is widespread in plants and show potential antimicrobial activity (Tabanca et al., 2001; Sonboli et al., 2005; Amor et al., 2008). It is attractive for Lepidoptera pests (Peterson et al., 1994; Mozuraitis, 2002) but it can also be a potent insecticide for others (Zhu and Tian, 2013; Benelli et al., 2019). It is a biomarker for heat (Oster et al., 2015) and drought (Khakdan et al., 2021) stress in plants. | -Antimicrobial activity |
| 2,4-dimethyl-heptane |  |  |  |  |  | Is a soil volatile organic compound that can be emitted in response to soil warming and nitrogen deposition (Romero-Olivares et al., 2022). Can be a fungal metabolite for Acinetobacter, Klebsiella, and P. mirabilis (Rees et al., 2018). In the Eucalyptus grandis rhizosphere, produced a significant avoidance effect on E. fetida (Zhiqun et al., 2017). | -Antimicrobial activity |
| 2,4,6-trimethyl-heptane |  |  |  |  |  | Reported in Angelica gigas (Seo et al., 2007). |  |
| Hexanal |  |  |  |  |  | It is a plant metabolite present in essential oils (Karagoz et al., 2017), fruits (Wen et al., 2014; Zhao et al., 2021) and macrophytes (Qiming et al., 2006). Has antifungal (Gardini et al., 1997; Fan et al., 2006; Li, Zhang, Lv, et al., 2021; Li, Zhang, Zhai, et al., 2021), and antibacterial (Kubo et al., 1999; Patrignani et al., 2008) activities by acting on microbial cell membranes, and can be used as a food additive to extend fruit shelf life and retain their original colour (Lanciotti et al., 2004). | -Antifungal and antibacterial activity. |
| 2-ethyl-hexanal |  |  |  |  |  | It is a fungal volatile organic compound that plays important ecophysiological roles in mediating inter-kingdom signalling (Hung et al., 2014). In A. thaliana it is able to hinder seedling formation (Hung et al., 2014). Has food preserving properties against microbial growth (Burgut, 2022). | -Plant-microbe interactions and microbe-microbe associations. |
| isopropyl alcohol  (2-propanol) |  |  |  |  |  | It is widely used as a solvent and chemical intermediate (Papa, 2011) and as biofuel (Lee et al., 1995), but also has potential antibacterial activity (Bashir et al., 2022). Can be a plant metabolite (Kameoka et al., 1994; Jabalpurwala et al., 2009) but mainly a bacterial metabolite in Clostridium sp. (Chen and Hiu, 1986; Ng et al., 2013; Charubin and Papoutsakis, 2019). It can be metabolised by different bacterial genera including Bacillus (Bustard et al., 2002), Sphingobacterium (Mohammad et al., 2006) and Methanoculleus (Tonouchi, 2004), and fungi (Ammazzalorso et al., 2008). | -Antimicrobial activity |
| methyl salicylate |  |  |  |  |  | It is a plant metabolite (Bisio et al., 1998; Miles, 2007; Yun and Chen, 2011; Dasgupta and Wahed, 2021; Frick et al., 2023) used as a signal molecule associated with systemic acquired resistance and hypersensitive reaction response after infection to induce programmed cell death (Yun and Chen, 2011). It can also be used to enhance secondary metabolite production in plants (Ramabulana et al., 2020), as a postharvest treatment maintaining bioactive compounds and nutritional quality (Habibi et al., 2020) or for insect oviposition deterrence (Groux et al., 2014). On the other hand it can be a factor promoting fungal sporulation (Hountondji et al., 2006) and attracting insects (De Boer and Dicke, 2004; Yuan et al., 2022), thus generating mixed effects on plant resistance to insect herbivores (Rowen et al., 2017). In humans, the action of methyl salicylate is multimodal with analgesic and anti-inflammatory properties (Miles, 2007; Dasgupta and Wahed, 2021) . |  |
| n-tridecan-1-ol |  |  |  |  |  | Present in various medicinal (Giordani et al., 2008; Tabanca et al., 2014; Chatterjee et al., 2018) and tropical plants (Silva et al., 1984; Pavithra et al., 2009; Mitra et al., 2017). Could be useful for the natural mosquito control agents (Tabanca et al., 2014) and possible bacterial biocontrol (Pavithra et al., 2009; Chatterjee et al., 2018) but it can also lure insect pests (Mitra et al., 2017). | -Antimicrobial agent |
| o-xylene |  |  |  |  |  | This is a common plant metabolite (Flath and Forrey, 1977; MacLeod et al., 1982; Kameoka et al., 1991; Pino et al., 2005). |  |
| methoxy-phenyl-oxime |  |  |  |  |  | Can be found in Muscodor sp. with apparent antimicrobial activity against diverse microbes (Siri-udom et al., 2016). | -Antimicrobial agent |
| p-xylene |  |  |  |  |  | Is a plant metabolite (Flath and Forrey, 1977; MacLeod et al., 1982; Lwande and Bentley, 1987; Pino et al., 2003). Can be utilised by Pseudomonas sp., (Collins and Daugulis, 1999; Miri et al., 2022) and other bacteria (Bramucci et al., 2002) to produce terephthalic acid. |  |
| pentacos-1-ene |  |  |  |  |  | It is mostly found in insects, where it may play a role in sex and species recognition (Mudd et al., 1982; Antony et al., 1985; McAuslane et al., 1990; Maile et al., 2000; Okosun et al., 2015), but can also be found in plants (Omata et al., 1991; Ramaroson-Raonizafinimanana et al., 1997; Wesolowska et al., 2015). | -Pheromone |
| pentanal |  |  |  |  |  | Reported a wide array of plants (Anjou et al., 1967; Goetz-Schmidt and Schreier, 1986; MacLeod et al., 1988; Pino et al., 2005), in macrophytes (Qiming et al., 2006) and in fungi (Goetz-Schmidt and Schreier, 1986). |  |
| 2-methoxy-3-pyrazine,  (1-methylethyl) |  |  |  |  |  | Is a Harmonia axyridis beetles’ defence metabolite (Cai et al., 2007), important for flavour in some wine varieties (Pickering et al., 2015). It is also found in coffee (Cantergiani et al., 2001), ginger (Kurobayashi et al., 1991) and ginseng (Iwabuchi et al., 1984). | -Flavour |
| 2-acetyl-resorcinol |  |  |  |  |  | Found in many plant systems (Orabi et al., 1991; Yang et al., 2011; Pradhan et al., 2022), with potential antimicrobial activities (Orabi et al., 1991; Li, Huang, et al., 2020; Xue et al., 2020) and inhibition and scavenging of free radicals (Li, Huang, et al., 2020; Pradhan et al., 2022). | -Antimicrobial agent |
| styrene |  |  |  |  |  | Produced in large quantities for polymerization (Chen et al., 2006) with mutagenic properties (Miller et al., 1994). It was first isolated from Styrax species but also found on Rhodiola rosacea (Rohloff, 2002), and Foeniculum vulgare seeds (Diao et al., 2014). |  |
| topotecan |  |  |  |  |  | Obtained by the extraction of stem and fruit of the plant Camptotheca acuminata to be extensively used to treat various cancers and also to treat certain viral diseases (Mamkulathil Devasia et al., 2021). It can also be obtained from Ophiorrhiza mungos callus. | -Antitumour agent |
| trans-2-undecen-1-ol |  |  |  |  |  | Previously reported in other plant species (Lija-Escaline et al., 2015; Priyadarshi et al., 2016; Afshari et al., 2021; Xiong et al., 2022). Its concentration is dependent upon nitrogen (Xiong et al., 2022) and silicon (Afshari et al., 2021) application. It has potential mosquito larvicidal properties (Lija-Escaline et al., 2015). |  |

| Compound | Region | | | | | Potential role |  |
| --- | --- | --- | --- | --- | --- | --- | --- |
| 1-linoleoyl glycerol |  |  |  |  |  | Found in roots and rhizomes of various plant systems (Reinecke and Zhao, 1988; Okuyama et al., 2001; Zhao et al., 2013; Jo et al., 2018), in fungi (Pradhan et al., 2022) and in microbials (Thanomsub et al., 2004; Z. Chen et al., 2018; Mahmoud et al., 2018; Vu et al., 2018), with possible analgesic properties (Okuyama et al., 2001; Z. Chen et al., 2018), antimicrobial activities (Vu et al., 2018), and high surfactant potency (Thanomsub et al., 2004). | -Antimicrobial activity |
| 16-hydroxyhexadecanoic acid (ω-hydroxypalmitic acid) |  |  |  |  |  | It is a key monomer of cutin in the plant cuticle (Peschel et al., 2007, 2007; Fernández-Álvarez et al., 2012; Shao et al., 2019), involved in the wound suberization process in plants (Lotfy et al., 1995; Wei et al., 2020). Can serve as subtract (Matsunaga et al., 2000) or be produced (Sung et al., 2015) by bacteria. |  |
| 2-hydroxycinnamic acid (o-coumaric acid) |  |  |  |  |  | Major compound of Cinnamomum cassia (Ngoc et al., 2012, 2014), also present in Glaucium flavum (Boulaaba et al., 2019), Psidium guajava (Simão et al., 2017), Eupatorium adenophorum (Zheng et al., 2012), Raphanus sativus L (Beevi et al., 2010), as well as other plants (Azar et al., 1987; Veneziani et al., 1999; Zeng and Mallik, 2006; Muceneeki et al., 2009). It can also be a microbial product (Lija-Escaline et al., 2015) or metabolized by fungi (Diao et al., 2014). Has great medical potential as anticoagulative, antitumor (Awe et al., 2009), anti-inflammatory, anti-atherosclerotic, immunostimulatory and antidiabetic (Neetu et al., 2020; Keman and Soyer, 2019). It also has antiviral, and antibacterial properties, including antibiotic-resistant bacteria (Keman and Soyer, 2019; Neetu et al., 2020), as well as antifungal properties (Daayf et al., 2000; Faria et al., 2011; Simão et al., 2017). Adding to this list, it has potent antioxidant activity (Beevi et al., 2010; Kostikova and Shaldaeva, 2017; Taofiq et al., 2017; Neetu et al., 2020). O-coumaric acid is also a phytotoxin involved in allelopathic phenomena (Ngoc et al., 2014, 2014) and is able to inhibit seedling development regardless of concentration (Li et al., 1993). | -Anticoagulative  -Antitumor  -Anti-inflammatory  -Anti-atherosclerotic  -Immunostimulatory  -Antidiabetic  -Antiviral  -Antibacterial |
| 2-oxoglutaric acid (α-ketoglutaric acid) |  |  |  |  |  | Is an important dicarboxylic acid and intermediate in the tricarboxylic acid cycle and amino acid metabolism (Otto et al., 2011; Huergo and Dixon, 2015), with particular industrial interest due to its broad application scope (Otto et al., 2011). It is produced in virtually all organisms (Otto et al., 2011; Legendre et al., 2022) as it participates in a variety of biological processes including anti-oxidative defence, energy production, signalling modules, and genetic modification (Legendre et al., 2020, 2022). |  |
| 6-methyl citrate |  |  |  |  |  | Found in Rosa bracteate (Talapatra et al., 2010) and Rhus parviflora (Xu et al., 2003)fruits. |  |
| 4-hydroxybenzoic acid |  |  |  |  |  | Has a great potential in biological applications due to its putative antiviral activity against SARS-CoV-2 and its antimicrobial activity towards multidrug-resistant pathogens (Youssef et al., 2021; Singab et al., 2022). It can be used by bacteria as a carbon source (Huang et al., 2008) and its concentration levels can be increased in the presence of methyl-jasmonate (Sircar et al., 2011). It has demonstrated allelopathic effects on seedling growth (Hussain et al., 2018; Leyte-Lugo et al., 2020; Reveglia et al., 2021), probably linked to its influence on the rhizosphere microbial composition (Jin et al., 2020). | -Antimicrobial agent  -Antiviral agent |
| 5-methoxysalicylic acid |  |  |  |  |  | Ubiquitous in plants (Kong et al., 2001) and bacteria (Alanis-Sánchez et al., 2019). |  |
| 9-oxo-10(E),12(E)-octadecadienoic acid |  |  |  |  |  | It is a fatty acid found in fungi (Gusakova and Khomova, 1984; Cantrell et al., 2008) and plants (Binder et al., 1964; Gusakova and Khomova, 1984; Kimura and Yokota, 2004; Kim et al., 2012) with antifungal (Legendre et al., 2022) and antibacterial (Kimura and Yokota, 2004) properties and a possible beneficial role in obesity-induced dyslipidemia and hepatic steatosis (Kim et al., 2012). |  |
| α,α-trehalose |  |  |  |  |  | Trehalose is a stress protectant in many organisms, including green algae and primitive plants (Lunn et al., 2014), with potential signal metabolite functions in microbe and insect interactions. It is also implicated in responses to cold and salinity, and in regulation of stomatal conductance and water-use efficiency (Lunn et al., 2014). Trehalose accumulates in stressed cells conferring protection from oxidative damage (Benaroudj et al., 2001; Herdeiro et al., 2006). It also has a potential role in insect pest control (Chen et al., 2010). | -Plant and animal metabolism |
| asparagine |  |  |  |  |  | Asparagine is an important nitrogen transport and storage compound in plants due to its high nitrogen/carbon ratio and stability (Hippe, 1988; García-Calderón et al., 2017). Plants have, for reasons of nutrition, a low level of Asparagine and use it via transamination for biosynthesis of glutamic acid (Činčerová, 1969). Can also be used by microorganisms as a nitrogen (Khaoua et al., 1991) or carbon source (Sini et al., 1995) influencing the rhizosphere microbial composition (Sini et al., 1995). | -Plant metabolism, nitrogen transport and storage. |
| 2-hydroxy-benzaldehyde (salicylaldehyde) |  |  |  |  |  | It is found in many plant systems (Yamaguchi and Shibamoto, 1979; Pino et al., 2003; Baser et al., 2006) but it can also be produced by bacteria (Tomás‐Gallardo et al., 2014). Can be used in metal ions absorbent formulations (Hussain et al., 2020) and has insect repellent, mycotoxigenic, antimicrobial (Gross et al., 2002; Kim et al., 2011) and nematicidal (Caboni et al., 2013) activities. | -Antimicrobial activity |
| D-(+)-galactose |  |  |  |  |  | It is similar to glucose in its structure and it has been identified as a trace component of some seeds and pulses (Williams, 2003). |  |
| D-(+)-malic acid  (D-malate) |  |  |  |  |  | D-malic acid is a metabolite of the Krebs cycle, useful in leaching biogenic elements from the substratum, like phosphorus and biometals (Grzesiak et al., 2021). It is found in plants (Sun et al., 2011; Nouioui et al., 2017; Lamine et al., 2023) and cyanobacteria (G. Wang et al., 2013; Wang et al., 2018; Grzesiak et al., 2021). It can be produced by bacteria (Miyama and Nakayama, 1993; Nakayama and Ushijima, 1993) or assimilated by them for growth (Hopper et al., 1970; Sun et al., 2011; G. Wang et al., 2013; Wang et al., 2018) including soil microbes (Sun et al., 2011; G. Wang et al., 2013; Zhang et al., 2014; Nouioui et al., 2017; Wang et al., 2018), as well as (Chodak et al., 2016; Hao et al., 2017; Liu et al., 2017; Guo et al., 2018, 2019). Anaerobic metabolism can occur (Martínez-Luque et al., 2001; Lukas et al., 2010) but most probably lead to D-malate accumulation in plants under stress (Liu et al., 2017; Guo et al., 2018, 2019). D-malate can also be attractive to fungi (Tunchai et al., 2017), secreted (Šimonovičová et al., 2020), or used by them (Côrte-Real et al., 1989; Ushio et al., 1992). Has potential hepatoprotective via inhibition of β -glucuronidase (Karak et al., 2017). | -Metabolic roles |
| D-(+)-tryptophan |  |  |  |  |  | Is a chiral building block for synthesis of pharmaceuticals, food additives, and agrochemicals (Kino et al., 2007; Zhang et al., 2016) and potential to enhance antibacterial activity (Kan et al., 2018; Elafify et al., 2019; Ghosh et al., 2019; Pourhajibagher et al., 2023). It can be an auxin precursor (Rekoslavskaya, 1986; Cooney and Nonhebel, 1991). | -Antibacterial activity |
| dodecanedioic acid |  |  |  |  |  | Dodecanedioic acid is highly useful in the chemical industry as a precursor of nylon-6,12, used in many technical applications (Funk et al., 2017). It can be naturally produced by Candida sp. (Lee et al., 1995; Funk et al., 2017). |  |
| fraxetin (7,8-dihydroxy-6-methoxycoumarin) |  |  |  |  |  | A hydroxycoumarin, widespread in plants (Konovalova et al., 1976; Narantuyaa et al., 1986; Garcia and Guerreiro, 1988; Pistelli et al., 1996; Yanishlieva and Marinova, 1996) and also produced by fungi (Sun et al., 2023) with potent antioxidant (Yanishlieva and Marinova, 1996; Fernández-Puntero et al., 2001; Fylaktakidou et al., 2004; Lin et al., 2008; Thuong et al., 2010; Shilova and Korotkova, 2017; Sun et al., 2023) and anti-inflammatory (Fylaktakidou et al., 2004; Choi et al., 2017) properties, as well as anti-cancer, neuroprotective (An et al., 2020) and anti-enteritis (Miao et al., 2021) effects, and notable antimicrobial properties (Céspedes et al., 2006; Yang et al., 2017). Coumarins have been shown to play an important role in assembly of the plant microbiomes and iron homeostasis (Rajniak et al., 2018; Siwinska et al., 2018; Sun et al., 2023). Fraxetin has a critical role in plant survival on substrates with high pH and limited iron availability (Vélez-Bermúdez and Schmidt, 2023). | -Antioxidant  -Anti-inflammatory  -Anti-tumour  -Neuroprotective  -Anti-enteritis |
| fraxin (7,8-dihydroxy-6-methoxycoumarin-8-beta-D-glucoside) |  |  |  |  |  | First described in Fraxinus sp. (Vasilevskaya et al., 1994; Iossifova et al., 1997; Kim et al., 2002; Lin et al., 2008; Ma and Zhao, 2008), but can be also obtained from other plant sources (Dubois et al., 1990; Morikawa et al., 2003; Whang et al., 2005; Lin et al., 2008; Li et al., 2011). It has remarkable free radical scavenging and cell protective effects against oxidative stress (Whang et al., 2005; Lin et al., 2008). | -Antioxidant |
| gentisic acid (2,5-dihydroxybenzoic acid) |  |  |  |  |  | It is a metabolic derivative of salicylic acid (Qi et al., 2012) with a putative role in malaria treatment (Ndjonka et al., 2012). It can be a plant (Ayaz et al., 2000; Proestos et al., 2005; Krzaczek et al., 2014; Nowak and Kawka, 2014) and a microbial (Grund et al., 1992; Fuenmayor et al., 1998; Chen et al., 2002) metabolite, but it can also be utilised by fungi (Zhou et al., 2001). It has free radical scavenging activity (Bellés et al., 2006). It is a biomarker for non-necrotizing reactions of the host plant in plant-pathogen systems (Bellés et al., 2006). | -Antioxidant  -Antimalarial |
| gluconic acid |  |  |  |  |  | Gluconic acid is plant metabolite (Turner et al., 1980; Vidotti et al., 2006) and a dissolving mineral compound that can be produced and utilised by zinc- and phosphate-solubilizer soil microbes to dissolve and mobilise unavailable phosphorus and zinc, which influences plant growth and flowering (Kaur et al., 2006, 2021; Kanse et al., 2015; Stephen et al., 2015; Parween et al., 2017; Mohd Din et al., 2020; Masood et al., 2022). It is produced by fungi, with antifungal activities (Kanse et al., 2015; Kaur et al., 2021), but also a biomarker in plant-pathogen systems (Peluffo et al., 2010; Hong et al., 2012) and abiotic stress conditions (Geilfus et al., 2015; Drapal et al., 2017). | -Plant-microbe and microbe-microbe interactions |
| L-phenylalanine |  |  |  |  |  | L-Phenylalanine is an essential amino acid, commercially important because of the consumer demand for low-calorie artificial sweetener aspartame (Ding et al., 2014). It can be produced by plants (Hounsome et al., 2008; Cotinguiba et al., 2022) and bacteria (Ding et al., 2014). Its high production in plants is associated with photosynthesis (Sankawa, 1999). |  |
| L-tyrosine |  |  |  |  |  | Tyrosine phosphorylation events modify bacterial and archaeal proteomes, bestowing cells with rapid and reversible responses to specific environmental stimuli or niches (Surwase and Jadhav, 2011) including general stress responses (León, 2022; Francis et al., 2023). Furthermore, L-Tyrosine is a precursor of L-DOPA with high potency used against Parkinson’s disease (Surwase and Jadhav, 2011). |  |
| oleamide |  |  |  |  |  | Oleamide is a fatty acid derivative known for its potential role in the mechanisms that mediate the drive to sleep, has profound effects on thermoregulation and acts as an analgesic (Mueller and Driscoll, 2009) and a potent hypolipidemic agent (Cheng et al., 2010). Oleamide may also play a role during apoptosis (Mueller and Driscoll, 2009), has putative neuroprotective (Reyes-Soto et al., 2022) and anti-tumour (Sun et al., 1995) properties. Besides, oleamide shows algicide properties (Shao et al., 2016). It can be found in plants (Arturo-Perdomo et al., 2021). | -Antioxidant  -Anti-tumour  -Neuroprotective |
| phloroglucinol |  |  |  |  |  | It is a phenolic compound able to attenuate tumour growth and angiogenesis (C. Chen et al., 2018). Reported in plants (Nicollier et al., 1983; Hammond and Mahlberg, 1994; Rivero-Cruz, 2008; Karthik et al., 2016) and algae (Koch and Gregson, 1984; Hammond and Mahlberg, 1994; Sailler and Glombitza, 1999; Wisespongpand and Kuniyoshi, 2003; Zou et al., 2008; Aremu et al., 2015). In bacteria, it can only be synthesized by some species of Pseudomonads (Yang and Cao, 2012). Presents antioxidant DPPH-scavenging, H2O2-scavenging and metal chelating properties (Karthik et al., 2016). It is able to improve germination and plant growth, including during micropropagation (Hammatt, 1994; Sarkar and S. Naik, 2000; Ainsley et al., 2001; Aremu et al., 2015; Pérez et al., 2016) and in M. whitei (Baskaran et al., 2022); and can be metabolized by fungi (Walker and Taylor, 1983; Patel et al., 1990). | -Anti-tumour  -Plant health |
| scopoletin |  |  |  |  |  | It is a coumarin present in some plant species (Briggs et al., 1976; Bubenchikova, 1990; Runkel et al., 1997; Debenedetti et al., 1998; Silva et al., 2002; Lerat et al., 2009; Kacem et al., 2012; Zakaria et al., 2012; Lee et al., 2013; Mogana et al., 2013; Ahmadi et al., 2019; Liu et al., 2019, 2023). Has potential anti-tumour, anti-inflammatory (Ahmadi et al., 2019) and neuroprotective properties (Mogana et al., 2013; Kashyap et al., 2020), but also potent peroxyl radical-scavenging capacity, reducing capacity, and cellular antioxidant capacity (Lee et al., 2013). In plants it has possible implications in plant-mycorrhizal fungi communication, by mitigating incompatibilities (Cosme et al., 2021) and can have antifungal (Silva et al., 2002) and antitoxin properties (Lerat et al., 2009). | -Antioxidant  -Anti-tumour  -Neuroprotective  -Plant-microbe interactions |
| stearamide |  |  |  |  |  | It is a primary fatty acid amide (Dembitsky et al., 2000), found in algae (Dembitsky et al., 2000; Bertin et al., 2012), fungi (Grove and Pople, 1981) and plants (Li et al., 2022), with cytotoxic and ichthyotoxic activity (Bertin et al., 2012). |  |
| sulcatol (6-methylhept-5-en-2-ol) |  |  |  |  |  | A green-herbaceous aromatic compound (Aprea et al., 2017) that is present in plants (Wilson et al., 1985; Wu et al., 1990; Jørgensen et al., 2000; Miyazawa and Kawata, 2006; Aprea et al., 2017) and can be produced by fungi (Demyttenaere et al., 2004; Qiu et al., 2014). |  |
| terephthalic acid |  |  |  |  |  | Terephthalic acid is widely used in the chemical industry to produce artificial fibre and plastic (Yastrebova et al., 2022). The complex structure, poor biodegradability and high toxicity pose great challenges for the environment and human health (Kudisi et al., 2022) and can degraded (Yastrebova et al., 2022) by bacteria. |  |
| trans-3-indoleacrylic acid |  |  |  |  |  | It is a plant growth hormone (Hao et al., 2019; Connell et al., 2022) that is also found in algae (Davyt et al., 1998), and has anti-inflammatory and anti-oxidative effects (Connell et al., 2022). |  |
| vanillin |  |  |  |  |  | Vanillin is an aromatic compound which is frequently used in food and cosmetics (Taira et al., 2018). Found in plants (Nascimento, 1999; Lin et al., 2000; Toyoda et al., 2006; Kuo et al., 2008). It can also be produced (Shettigar et al., 2018; Taira et al., 2018; García-Hidalgo et al., 2020) or metabolized by microorganisms (Zhou et al., 2022). It has putative anti-platelet aggregation properties (Lin et al., 2000). | -Flavour |

SI References

Abanda-Nkpwatt, D., Krimm, U., Schreiber, L., and Schwab, W. (2006) Dual Antagonism of Aldehydes and Epiphytic Bacteria from Strawberry Leaf Surfaces against the Pathogenic Fungus Botrytis cinerea in vitro. Biocontrol 51: 279–291.

Adewunmi, Y., Namjilsuren, S., Walker, W.D., Amato, D.N., Amato, D.V., Mavrodi, O.V., et al. (2020) Antimicrobial Activity of, and Cellular Pathways Targeted by, p -Anisaldehyde and Epigallocatechin Gallate in the Opportunistic Human Pathogen Pseudomonas aeruginosa. Appl Environ Microbiol 86: e02482-19.

Afshari, M., Pazoki, A., and Sadeghipour, O. (2021) Foliar‐applied Silicon and its Nanoparticles Stimulate Physio‐chemical Changes to Improve Growth, Yield and Active Constituents of Coriander (Coriandrum Sativum L.) Essential Oil Under Different Irrigation Regimes. Silicon 13: 4177–4188.

Ahmad, A. and Misra, L.N. (1994) Terpenoids from Artemisia annua and constituents of its essential oil. Phytochemistry 37: 183–186.

Ahmadi, N., Mohamed, S., Sulaiman Rahman, H., and Rosli, R. (2019) Epicatechin and scopoletin‐rich Morinda citrifolia leaf ameliorated leukemia via anti‐inflammatory, anti‐angiogenesis, and apoptosis pathways in vitro and in vivo. J Food Biochem 43:.

Ainsley, P.J., Collins, G.G., and Sedgley, M. (2001) In vitro rooting of almond (Prunus dulcis Mill.). In Vitro CellDevBiol-Plant 37: 778–785.

Akter, K., Barnes, E.C., Loa-Kum-Cheung, W.L., Yin, P., Kichu, M., Brophy, J.J., et al. (2016) Antimicrobial and antioxidant activity and chemical characterisation of Erythrina stricta Roxb. (<i>Fabaceaev). Journal of Ethnopharmacology 185: 171–181.

Alanis-Sánchez, B.M., Pérez-Tapia, S.M., Vázquez-Leyva, S., Mejía-Calvo, I., Macías-Palacios, Z., Vallejo-Castillo, L., et al. (2019) Utilization of naproxen by Amycolatopsis sp. Poz 14 and detection of the enzymes involved in the degradation metabolic pathway. World J Microbiol Biotechnol 35: 186.

Ammazzalorso, A., Amoroso, R., Bettoni, G., De Filippis, B., Fantacuzzi, M., Giampietro, L., et al. (2008) Candida rugosa lipase-catalysed kinetic resolution of 2-substituted-aryloxyacetic esters with dimethylsulfoxide and isopropanol as additives. Chirality 20: 115–118.

Amor, I., Neffati, A., Ben Sgaier, M., Bhouri, W., Boubaker, J., Skandrani, I., et al. (2008) Antimicrobial Activity of Essential Oils Isolated from Phlomis crinita Cav. ssp. mauritanica Munby. J Am Oil Chem Soc 85: 845–849.

An, S.H., Choi, G.-S., and Ahn, J.-H. (2020) Biosynthesis of fraxetin from three different substrates using engineered Escherichia coli. Appl Biol Chem 63: 55.

Andati, R.E., Omolo, M.O., and Ndiege, I.O. (2021) Ovicidal Activity of 2-Hydroxy-4-Methoxybenzaldehyde, Derivatives and Structural Analogues on Anopheles gambiae eggs, Ecology.

Andersen, R.A. (2006) Final Report on the Safety Assessment of Benzaldehyde. Int J Toxicol 25: 11–27.

Anjou, K., von Sydow, E., Nielsen, P.H., Brunvoll, J., and Hagen, G. (1967) The Aroma of Cranberries. II. Vaccinium macrocarpon Ait.. Acta Chem Scand 21: 2076–2082.

Antony, C., Davis, T.L., Carlson, D.A., Pechine, J.-M., and Jallon, J.-M. (1985) Compared behavioral responses of male Drosophila melanogaster (Canton S) to natural and synthetic aphrodisiacs. J Chem Ecol 11: 1617–1629.

Aprea, E., Charles, M., Endrizzi, I., Laura Corollaro, M., Betta, E., Biasioli, F., and Gasperi, F. (2017) Sweet taste in apple: the role of sorbitol, individual sugars, organic acids and volatile compounds. Sci Rep 7: 44950.

Aremu, A.O., Cheesman, L., Finnie, J.F., and Van Staden, J. (2011) M.whitei (Apocynaceae): A review of its biological activities, conservation strategies and economic potential. South African Journal of Botany 77: 960–971.

Aremu, A.O., Masondo, N.A., Rengasamy, K.R.R., Amoo, S.O., Gruz, J., Bíba, O., et al. (2015) Physiological role of phenolic biostimulants isolated from brown seaweed Ecklonia maxima on plant growth and development. Planta 241: 1313–1324.

Arturo-Perdomo, D., Mora, J.P.J., Ibáñez, E., Cifuentes, A., Hurtado-Benavides, A., and Montero, L. (2021) Extraction and Characterization of the Polar Lipid Fraction of Blackberry and Passion Fruit Seeds Oils Using Supercritical Fluid Extraction. Food Anal Methods 14: 2026–2037.

Attar, U., Hinge, V., Zanan, R., Adhav, R., and Nadaf, A. (2017) Identification of aroma volatiles and understanding 2-acetyl-1-pyrroline biosynthetic mechanism in aromatic mung bean ( Vigna radiata (L.) Wilczek). Physiol Mol Biol Plants 23: 443–451.

Awe, S., Mikolasch, A., and Schauer, F. (2009) Formation of coumarines during the degradation of alkyl substituted aromatic oil components by the yeast Trichosporon asahii. Appl Microbiol Biotechnol 84: 965–976.

Ayaz, F.A., Kucukislamoglu, M., and Reunanen, M. (2000) Sugar, Non-volatile and Phenolic Acids Composition of Strawberry Tree (Arbutus unedo L. var.ellipsoidea ) Fruits. Journal of Food Composition and Analysis 13: 171–177.

Azar, M., Verette, E., and Brun, S. (1987) Identification of Some Phenolic Compounds in Bilberry Juice Vaccinium myrtillus. J Food Science 52: 1255–1257.

Aziz, A.N., Ibrahim, H., Rosmy Syamsir, D., Mohtar, M., Vejayan, J., and Awang, K. (2013) Antimicrobial compounds from Alpinia conchigera. Journal of Ethnopharmacology 145: 798–802.

Barros, J., Becerra, J., González, C., and Martínez, M. (2013) Antibacterial metabolites synthesized by psychrotrophic bacteria isolated from cold-freshwater environments. Folia Microbiol 58: 127–133.

Baser, K.H.C., Demirci, B., Iscan, G., Hashimoto, T., Demirci, F., Noma, Y., and Asakawa, Y. (2006) The Essential Oil Constituents and Antimicrobial Activity of Anthemis aciphylla Boiss. var. discoidea Boiss. Chem Pharm Bull 54: 222–225.

Bashir, M.H., Hollingsworth, A., Thompson, J.D., Shortridge, D., Lake, S.P., Deeken, C.R., et al. (2022) Antimicrobial performance of two preoperative skin preparation solutions containing iodine and isopropyl alcohol. American Journal of Infection Control 50: 792–798.

Baskaran, P., Naidu, S., and Van Staden, J. (2022) Conservation and Determination of Nutritional Composition of M.whitei In Vitro. In Plant Genetic Resources, Inventory, Collection and Conservation. Ramamoorthy, S., Buot, I., and Chandrasekaran, R. (eds). Singapore: Springer Nature Singapore, pp. 505–518.

Battinelli, L., Daniele, C., Cristiani, M., Bisignano, G., Saija, A., and Mazzanti, G. (2006) In vitro antifungal and anti-elastase activity of some aliphatic aldehydes from Olea europaea L. fruit. Phytomedicine 13: 558–563.

Bedini, S., Flamini, G., Girardi, J., Cosci, F., and Conti, B. (2015) Not just for beer: evaluation of spent hops (Humulus lupulus L.) as a source of eco-friendly repellents for insect pests of stored foods. J Pest Sci 88: 583–592.

Beevi, S.S., Narasu, M.L., and Gowda, B.B. (2010) Polyphenolics Profile, Antioxidant and Radical Scavenging Activity of Leaves and Stem of Raphanus sativus L. Plant Foods Hum Nutr 65: 8–17.

Bellés, J.M., Garro, R., Pallás, V., Fayos, J., Rodrigo, I., and Conejero, V. (2006) Accumulation of gentisic acid as associated with systemic infections but not with the hypersensitive response in plant-pathogen interactions. Planta 223: 500–511.

Benaroudj, N., Lee, D.H., and Goldberg, A.L. (2001) Trehalose Accumulation during Cellular Stress Protects Cells and Cellular Proteins from Damage by Oxygen Radicals. Journal of Biological Chemistry 276: 24261–24267.

Benelli, G., Pavela, R., Cianfaglione, K., Nagy, D.U., Canale, A., and Maggi, F. (2019) Evaluation of two invasive plant invaders in Europe ( Solidago canadensis and Solidago gigantea ) as possible sources of botanical insecticides. J Pest Sci 92: 805–821.

Bertin, M.J., Zimba, P.V., Beauchesne, K.R., Huncik, K.M., and Moeller, P.D.R. (2012) Identification of toxic fatty acid amides isolated from the harmful alga Prymnesium parvum carter. Harmful Algae 20: 111–116.

Bicchi, C., Fresia, M., Rubiolo, P., Monti, D., Franz, C., and Goehler, I. (1997) Constituents of </i>Tagetes lucida</i> Cav. ssp. lucida Essential Oil. Flavour Fragr J 12: 47–52.

Binder, R.G., Applewhite, T.H., Diamond, M.J., and Goldblatt, L.A. (1964) Chromatographic analysis of seed oils. II. fatty acid composition of dimorphotheca oil. J Am Oil Chem Soc 41: 108–111.

Bisio, A., Ciarallo, G., Romussi, G., Fontana, N., Mascolo, N., Capasso, R., and Biscardi, D. (1998) Chemical composition of essential oils from some Salvia species. Phytother Res 12: S117–S120.

Blažytė-Čereškienė, L., Apšegaitė, V., and Būda, V. (2019) The choice between flowers of closely related plant species by generalist pollinator: identification of relevant VOCs. Arthropod-Plant Interactions 13: 735–743.

Boulaaba, M., Kalai, F.Z., Dakhlaoui, S., Ezzine, Y., Selmi, S., Bourgou, S., et al. (2019) Antioxidant, antiproliferative and anti-inflammatory effects of Glaucium flavum fractions enriched in phenolic compounds. Med Chem Res 28: 1995–2001.

Bramucci, M., McCutchen, C., Singh, M., Thomas, S., Larsen, B., Buckholz, J., and Nagarajan, V. (2002) Pure bacterial isolates that convert p-xylene to terephthalic acid. Applied Microbiology and Biotechnology 58: 255–259.

Briggs, L.H., Beachen, J.F., Cambie, R.C., Dudman, N.P.B., Steggles, A.W., and Rutledge, P.S. (1976) Chemistry of the Coprosma genus. Part XIV. Constituents of five New Zealand species. J Chem Soc, Perkin Trans 1 1789.

Bubenchikova, V.N. (1990) Coumarins of plants of the genus Centaurea. Chem Nat Compd 26: 709–709.

Buchbauer, Gerhard., Jirovetz, Leopold., Wasicky, Michael., and Nikiforov, Alexej. (1993) Headspace and essential oil analysis of apple flowers. J Agric Food Chem 41: 116–118.

Buescher, R.H. and Buescher, R.W. (2001) Production and Stability of (E, Z)-2, 6-Nonadienal, the Major Flavor Volatile of Cucumbers. J Food Science 66: 357–361.

Buettner, A., Mestres, M., Fischer, A., Guasch, J., and Schieberle, P. (2003) Evaluation of the most odour-active compounds in the peel oil of clementines (Citrus reticulata blanco cv. clementine). Eur Food Res Technol 216: 11–14.

Burgut, A. (2022) Characterization and microencapsulation of Lactobacillus plantarum FI 8595 cell free metabolites with enhanced antimicrobial property by powdered propolis. Food Measure 16: 4355–4363.

Bustard, M.T., Whiting, S., Cowan, D.A., and Wright, P.C. (2002) Biodegradation of high-concentration isopropanol by a solvent-tolerant thermophile, Bacillus pallidus. Extremophiles 6: 319–323.

Buttery, R.G., Kamm, J.A., and Ling, L.C. (1984) Volatile components of red clover leaves, flowers, and seed pods: Possible insect attractants. J Agric Food Chem 32: 254–256.

Byers, J.A. (1982) Male-specific conversion of the host plant compound, myrcene, to the pheromone, (+)-ipsdienol, in the bark beetle, Dendroctonus brevicomis. J Chem Ecol 8: 363–371.

Caboni, P., Aissani, N., Cabras, T., Falqui, A., Marotta, R., Liori, B., et al. (2013) Potent Nematicidal Activity of Phthalaldehyde, Salicylaldehyde, and Cinnamic Aldehyde against Meloidogyne incognita. J Agric Food Chem 61: 1794–1803.

Caboni, P., Ntalli, N.G., Aissani, N., Cavoski, I., and Angioni, A. (2012) Nematicidal Activity of ( E , E )-2,4-Decadienal and ( E )-2-Decenal from Ailanthus altissima against Meloidogyne javanica. J Agric Food Chem 60: 1146–1151.

Cai, L., Koziel, J.A., and O’Neal, M.E. (2007) Determination of characteristic odorants from Harmonia axyridis beetles using in vivo solid-phase microextraction and multidimensional gas chromatography–mass spectrometry–olfactometry. Journal of Chromatography A 1147: 66–78.

Cantergiani, E., Brevard, H., Krebs, Y., Feria-Morales, A., Amadò, R., and Yeretzian, C. (2001) Characterisation of the aroma of green Mexican coffee and identification of mouldy/earthy defect. European Food Research and Technology 212: 648–657.

Cantrell, C.L., Case, B.P., Mena, E.E., Kniffin, T.M., Duke, S.O., and Wedge, D.E. (2008) Isolation and Identification of Antifungal Fatty Acids from the Basidiomycete Gomphus floccosus. J Agric Food Chem 56: 5062–5068.

Carballeira, N.M., Sanabria, D., Cruz, C., Parang, K., Wan, B., and Franzblau, S. (2006) 2,6-hexadecadiynoic acid and 2,6-nonadecadiynoic acid: Novel synthesized acetylenic fatty acids as potent antifungal agents. Lipids 41: 507–511.

Céspedes, C.L., Avila, J.G., Martínez, A., Serrato, B., Calderón-Mugica, J.C., and Salgado-Garciglia, R. (2006) Antifungal and Antibacterial Activities of Mexican Tarragon ( Tagetes lucida ). J Agric Food Chem 54: 3521–3527.

Chamarthi, S.K., Vijay, P.M., Sharma, H.C., and Narasu, L.M. (2012) Constitutive and Inducible Resistance to Atherigona soccata (Diptera: Muscidae) in Sorghum bicolor. jnl econ entom 105: 1069–1076.

Chamberlain, D.A., Wilson, G., and Ryan, M.F. (1991) trans-2-Nonenal insect repellent, insecticide, and flavor compound in carrot roots, cell suspensions, and hairy root cultures. J Chem Ecol 17: 615–624.

Chambers, S.T., Bhandari, S., Scott-Thomas, A., and Syhre, M. (2011) Novel diagnostics: progress toward a breath test for invasive Aspergillus fumigatus. Med Mycol 49: S54–S61.

Chang, F.-R., Chen, C.-Y., Wu, P.-H., Kuo, R.-Y., Chang, Y.-C., and Wu, Y.-C. (2000) New Alkaloids from Annona purpurea. J Nat Prod 63: 746–748.

Chang, W. and Lee, S. (1998) Norneolignan and phenols from Curculigo capitulata. Phytochemistry 49: 2133–2136.

Charubin, K. and Papoutsakis, E.T. (2019) Direct cell-to-cell exchange of matter in a synthetic Clostridium syntrophy enables CO2 fixation, superior metabolite yields, and an expanded metabolic space. Metabolic Engineering 52: 9–19.

Chatterjee, S., Karmakar, A., Azmi, S.A., and Barik, A. (2018) Antibacterial Activity of Long-Chain Primary Alcohols from Solena amplexicaulis Leaves. Proc Zool Soc 71: 313–319.

Chen, C., Ye, Y., Wang, R., Zhang, Y., Wu, C., Debnath, S.C., et al. (2018) Streptomyces nigra sp. nov. Is a Novel Actinobacterium Isolated From Mangrove Soil and Exerts a Potent Antitumor Activity in Vitro. Front Microbiol 9: 1587.

Chen, C., Zhao, C., Wu, Z., Liu, G., Yu, X., and Zhang, P. (2021) Whitefly‐induced tomato volatiles mediate host habitat location of the parasitic wasp Encarsia formosa , and enhance its efficacy as a bio‐control agent. Pest Manag Sci 77: 749–757.

Chen, C.-H., Shaw, C.-Y., Chen, C.-C., and Tsai, Y.-C. (2002) 2,3,4-Trimethyl-5,7-dihydroxy-2,3-dihydrobenzofuran, a Novel Antioxidant, from Penicillium citrinum F5. J Nat Prod 65: 740–741.

Chen, H., Cao, S., Jin, Y., Tang, Y., and Qi, H. (2016) The Relationship between CmADHs and the Diversity of Volatile Organic Compounds of Three Aroma Types of Melon ( Cucumis melo ). Front Physiol 7:.

Chen, J., Zhang, D., Yao, Q., Zhang, J., Dong, X., Tian, H., et al. (2010) Feeding-based RNA interference of a trehalose phosphate synthase gene in the brown planthopper, Nilaparvata lugens: Feeding-based RNAi in Nilaparvata lugens. Insect Molecular Biology 19: 777–786.

Chen, J.-S. and Hiu, S.F. (1986) Acetone-butanol-isopropanol production by Clostridium beijerinckii. Biotechnol Lett 8: 371–376.

Chen, Q., Milburn, R., and Karellas, N. (2006) Real time monitoring of hazardous airborne chemicals: A styrene investigation. Journal of Hazardous Materials 132: 261–268.

Chen, Y.-C., Liao, C.-H., and Chen, I.-S. (2007) Lignans, an amide and anti-platelet activities from Piper philippinum. Phytochemistry 68: 2101–2111.

Chen, Z., Zheng, Z., Wang, F.-L., Niu, Y.-P., Miao, J.-L., and Li, H. (2018) Intracellular Metabolic Changes of Rhodococcus sp. LH During the Biodegradation of Diesel Oil. Mar Biotechnol 20: 803–812.

Cheng, M.-C., Ker, Y.-B., Yu, T.-H., Lin, L.-Y., Peng, R.Y., and Peng, C.-H. (2010) Chemical Synthesis of 9( Z )-Octadecenamide and Its Hypolipidemic Effect: A Bioactive Agent Found in the Essential Oil of Mountain Celery Seeds. J Agric Food Chem 58: 1502–1508.

Cho, I.H., Lee, H.J., and Kim, Y.-S. (2012) Differences in the Volatile Compositions of Ginseng Species (Panax sp.). J Agric Food Chem 60: 7616–7622.

Cho, M.J., Buescher, R.W., Johnson, M., and Janes, M. (2004) Inactivation of Pathogenic Bacteria by Cucumber Volatiles (E,Z)-2,6-Nonadienal and (E)-2-Nonenal. Journal of Food Protection 67: 1014–1016.

Cho, S., Nuijten, E., Shewfelt, R.L., and Kays, S.J. (2014) Aroma chemistry of African Oryza glaberrima and Oryza sativa rice and their interspecific hybrids: Aroma chemistry of African rice. J Sci Food Agric 94: 727–735.

Chodak, M., Klimek, B., and Niklińska, M. (2016) Composition and activity of soil microbial communities in different types of temperate forests. Biol Fertil Soils 52: 1093–1104.

Choi, H.G., Je, I.-G., Kim, G.J., Nam, J.-W., Shim, S.H., Kim, S.-H., and Choi, H. (2017) Chemical Constituents of the Root of Angelica tenuissima and their Anti-allergic Inflammatory Activity. Nat Prod Commun 12: 779–780.

Chu, S.S., Jiang, G.H., Liu, W.L., and Liu, Z.L. (2012) Insecticidal activity of the root bark essential oil of Periploca sepium Bunge and its main component. Natural Product Research 26: 926–932.

Chung, H., Chung, W.-Y., Yoo, E.-S., Cho, S.K., Oh, S.-K., and Kim, Y.-S. (2012) Characterization of volatile aroma-active compounds in Dangyooja (Citrus grandis Osbeck). J Korean Soc Appl Biol Chem 55: 133–136.

Činčerová, A. (1969) Effect of trophic conditions on asparagine transamination in wheat plants. Biol Plant 11: 139–148.

Cioffi, G., D’Auria, M., Braca, A., Mendez, J., Castillo, A., Morelli, I., et al. (2002) Antioxidant and Free-Radical Scavenging Activity of Constituents of the Leaves of Tachigalia paniculata. J Nat Prod 65: 1526–1529.

Collins, L.D. and Daugulis, A.J. (1999) Benzene/toluene/p -xylene degradation. Part II. Effect of substrate interactions and feeding strategies in toluene/benzene and toluene/ p -xylene fermentations in a partitioning bioreactor. Applied Microbiology and Biotechnology 52: 360–365.

Combet, E., Eastwood, D.C., Burton, K.S., Combet, E., Henderson, J., Henderson, J., and Combet, E. (2006) Eight-carbon volatiles in mushrooms and fungi: properties, analysis, and biosynthesis. Mycoscience 47: 317–326.

Connell, E., Le Gall, G., Pontifex, M.G., Sami, S., Cryan, J.F., Clarke, G., et al. (2022) Microbial-derived metabolites as a risk factor of age-related cognitive decline and dementia. Mol Neurodegeneration 17: 43.

Cooney, TerrenceP. and Nonhebel, HeatherM. (1991) Biosynthesis of indole-3-acetic acid in tomato shoots: Measurement, mass-spectral identification and incorporation of ?2H from ?2H2O into indole-3-acetic acid, d- and l-tryptophan, indole-3-pyruvate and tryptamine. Planta 184:.

Côrte-Real, M., Leão, C., and van Uden, N. (1989) Transport of l(-)malic acid and other dicarboxylic acids in the yeast Candida sphaerica. Appl Microbiol Biotechnol 31–31: 551–555.

Cortina, P.R., Asis, R., Peralta, I.E., Asprelli, P.D., and Santiago, A.N. (2016) Determination of Volatile Organic Compounds in Andean Tomato Landraces by Headspace Solid Phase Microextraction-Gas Chromatography-Mass Spectrometry. Journal of the Brazilian Chemical Society.

Cosme, M., Fernández, I., Declerck, S., van der Heijden, M.G.A., and Pieterse, C.M.J. (2021) A coumarin exudation pathway mitigates arbuscular mycorrhizal incompatibility in Arabidopsis thaliana. Plant Mol Biol 106: 319–334.

Cotinguiba, F., Debonsi, H.M., Silva, R.V., Pioli, R.M., Pinto, R.A., Felippe, L.G., et al. (2022) Amino acids L-phenylalanine and L-lysine involvement in trans and cis piperamides biosynthesis in two Piper species. Braz J Biol 82: e268505.

Cumeras, R., Aksenov, A.A., Pasamontes, A., Fung, A.G., Cianchetta, A.N., Doan, H., et al. (2016) Identification of fungal metabolites from inside Gallus gallus domesticus eggshells by non-invasively detecting volatile organic compounds (VOCs). Anal Bioanal Chem 408: 6649–6658.

Daayf, F., Ongena, M., Boulanger, R., El Hadrami, I., and Bélanger, R.R. (2000) Induction of Phenolic Compounds in Two Cultivars of Cucumber by Treatment of Healthy and Powdery Mildew-infected Plants with Extracts of Reynoutria sachalinensis. Journal of Chemical Ecology 26: 1579–1593.

Das, A. and Banik, B.K. (2021) Microwave-assisted oxidation and reduction reactions. In Microwaves in Chemistry Applications. Elsevier, pp. 199–244.

Dasgupta, A. and Wahed, A. (2021) Common poisonings including heavy metal poisoning. In Clinical Chemistry, Immunology and Laboratory Quality Control. Elsevier, pp. 405–419.

Davyt, D., Entz, W., Fernandez, R., Mariezcurrena, R., Mombrú, A.W., Saldaña, J., et al. (1998) A New Indole Derivative from the Red Alga Chondria atropurpurea . Isolation, Structure Determination, and Anthelmintic Activity. J Nat Prod 61: 1560–1563.

De Boer, J.G. and Dicke, M. (2004) The Role of Methyl Salicylate in Prey Searching Behavior of the Predatory Mite Phytoseiulus persimilis. J Chem Ecol 30: 255–271.

Debenedetti, S.L., Nadinic, E.L., Coussio, J.D., De Kimpe, N., and Boeykens, M. (1998) Two 6,7-dioxygenated coumarins from Pterocaulon virgatum. Phytochemistry 48: 707–710.

Dembitsky, V.M., Shkrob, I., and Rozentsvet, O.A. (2000) Fatty acid amides from freshwater green alga Rhizoclonium hieroglyphicum. Phytochemistry 54: 965–967.

Demyttenaere, J.C.R., Vanoverschelde, J., and De Kimpe, N. (2004) Biotransformation of (R)-(+)- and (S)-(−)-citronellol by Aspergillus sp. and Penicillium sp., and the use of solid-phase microextraction for screening. Journal of Chromatography A 1027: 137–146.

Deng, X., Liao, Q., Xu, X., Yao, M., Zhou, Y., Lin, M., et al. (2014) Analysis of Essential Oils from Cassia Bark and Cassia Twig Samples by GC-MS Combined with Multivariate Data Analysis. Food Anal Methods 7: 1840–1847.

Diao, W.-R., Hu, Q.-P., Zhang, H., and Xu, J.-G. (2014) Chemical composition, antibacterial activity and mechanism of action of essential oil from seeds of fennel (Foeniculum vulgare Mill.). Food Control 35: 109–116.

Ding, R., Liu, L., Chen, X., Cui, Z., Zhang, A., Ren, D., and Zhang, L. (2014) Introduction of two mutations into AroG increases phenylalanine production in Escherichia coli. Biotechnol Lett 36: 2103–2108.

Drapal, M., Farfan-Vignolo, E.R., Gutierrez, O.R., Bonierbale, M., Mihovilovich, E., and Fraser, P.D. (2017) Identification of metabolites associated with water stress responses in Solanum tuberosum L. clones. Phytochemistry 135: 24–33.

Dubois, M.-A., Wierer, M., and Wagner, H. (1990) Palustroside, a coumarin glucoside ester fromLedum palustre. Phytochemistry 29: 3369–3371.

Ehlers, D. and Pfister, M. (1997) Compounds of Vanillons (Vanilla pompona Schiede). Journal of Essential Oil Research 9: 427–431.

Elafify, M., Darwish, W.S., Al-Ashmawy, M., Elsherbini, M., Koseki, S., Kawamura, S., and Abdelkhalek, A. (2019) Prevalence of Salmonella spp. in Egyptian dairy products: molecular, antimicrobial profiles and a reduction trial using d-tryptophan. J Consum Prot Food Saf 14: 399–407.

Esmaeili, A. and Hashemi, E. (2011) Biotransformation of myrcene by Pseudomonas aeruginosa. Chemistry Central Journal 5: 26.

Fan, L., Song, J., Beaudry, R.M., and Hildebrand, P.D. (2006) Effect of Hexanal Vapor on Spore Viability of Penicillium expansum, Lesion Development on Whole Apples and Fruit Volatile Biosynthesis. Journal of Food Science 71: M105–M109.

Faria, N.C.G., Kim, J.H., Gonçalves, L.A.P., Martins, M. de L., Chan, K.L., and Campbell, B.C. (2011) Enhanced activity of antifungal drugs using natural phenolics against yeast strains of Candida and Cryptococcus: Natural antifungal chemosensitizers. Letters in Applied Microbiology 52: 506–513.

Fernández-Álvarez, A., Marín-Menguiano, M., Lanver, D., Jiménez-Martín, A., Elías-Villalobos, A., Pérez-Pulido, A.J., et al. (2012) Identification of O-mannosylated virulence factors in Ustilago maydis. PLoS Pathog 8: e1002563.

Fernández-Puntero, B., Barroso, I., Iglesias, I., Benedí, J., and Villar, A. (2001) Antioxidant Activity of Fraxetin: In Vivo and ex Vivo Parameters in Normal Situation versus Induced Stress. Biological & Pharmaceutical Bulletin 24: 777–784.

Fitzgerald, D.J., Stratford, M., Gasson, M.J., and Narbad, A. (2005) Structure−Function Analysis of the Vanillin Molecule and Its Antifungal Properties. J Agric Food Chem 53: 1769–1775.

Flath, R.A. and Forrey, R.R. (1977) Volatile components of papaya (Carica papaya L., Solo variety). J Agric Food Chem 25: 103–109.

Forlani, G., Occhipinti, A., Bossi, S., Bertea, C.M., Varese, C., and Maffei, M.E. (2011) Magnaporthe oryzae cell wall hydrolysate induces ROS and fungistatic VOCs in rice cell cultures. Journal of Plant Physiology 168: 2041–2047.

Formagio, A.S.N., Vieira, M. do C., dos Santos, L.A.C., Cardoso, C.A.L., Foglio, M.A., de Carvalho, J.E., et al. (2013) Composition and Evaluation of the Anti-Inflammatory and Anticancer Activities of the Essential Oil from Annona sylvatica A. St.-Hil. Journal of Medicinal Food 16: 20–25.

Francis, N., Behera, M.R., Natarajan, K., and Laishram, R.S. (2023) Tyrosine phosphorylation controlled poly(A) polymerase I activity regulates general stress response in bacteria. Life Sci Alliance 6: e202101148.

Frank, N., Dubois, M., and Huertas Pérez, J.F. (2020) Detection of Furan and five Alkylfurans, including 2-Pentylfuran, in various Food Matrices. Journal of Chromatography A 1622: 461119.

Frick, E.M., Sapkota, M., Pereira, L., Wang, Y., Hermanns, A., Giovannoni, J.J., et al. (2023) A family of methyl esterases converts methyl salicylate to salicylic acid in ripening tomato fruit. Plant Physiol 191: 110–124.

Froissard, D., Rapior, S., Bessière, J.-M., Buatois, B., Fruchier, A., Sol, V., and Fons, F. (2015) Asplenioideae Species as a Reservoir of Volatile Organic Compounds with Potential Therapeutic Properties. Natural Product Communications 10: 1934578X1501000.

Fuenmayor, S.L., Wild, M., Boyes, A.L., and Williams, P.A. (1998) A gene cluster encoding steps in conversion of naphthalene to gentisate in Pseudomonas sp. strain U2. J Bacteriol 180: 2522–2530.

Funk, I., Rimmel, N., Schorsch, C., Sieber, V., and Schmid, J. (2017) Production of dodecanedioic acid via biotransformation of low cost plant-oil derivatives using Candida tropicalis. Journal of Industrial Microbiology and Biotechnology 44: 1491–1502.

Fylaktakidou, K., Hadjipavlou-Litina, D., Litinas, K., and Nicolaides, D. (2004) Natural and Synthetic Coumarin Derivatives with Anti-Inflammatory / Antioxidant Activities. CPD 10: 3813–3833.

Garcia, E.E. and Guerreiro, E. (1988) Sesquiterpene lactones from Gochnatia palosanto and coumarins from G. argentina. Phytochemistry 27: 288–290.

García-Calderón, M., Pérez-Delgado, C.M., Credali, A., Vega, J.M., Betti, M., and Márquez, A.J. (2017) Genes for asparagine metabolism in Lotus japonicus: differential expression and interconnection with photorespiration. BMC Genomics 18: 781.

García-Hidalgo, J., Brink, D.P., Ravi, K., Paul, C.J., Lidén, G., and Gorwa-Grauslund, M.F. (2020) Vanillin Production in Pseudomonas : Whole-Genome Sequencing of Pseudomonas sp. Strain 9.1 and Reannotation of Pseudomonas putida CalA as a Vanillin Reductase. Appl Environ Microbiol 86: e02442-19.

Gardini, F., Lanciotti, R., Caccioni, D.R.L., and Guerzoni, M.E. (1997) Antifungal Activity of Hexanal As Dependent on Its Vapor Pressure. J Agric Food Chem 45: 4297–4302.

Gardner, H.W., Dornbos, D.L., and Desjardins, A.E. (1990) Hexanal, trans-2-hexenal, and trans-2-nonenal inhibit soybean, Glycine max, seed germination. J Agric Food Chem 38: 1316–1320.

Geilfus, C.-M., Niehaus, K., Gödde, V., Hasler, M., Zörb, C., Gorzolka, K., et al. (2015) Fast responses of metabolites in Vicia faba L. to moderate NaCl stress. Plant Physiology and Biochemistry 92: 19–29.

George, J., Ravishankar, G.A., Pereira, J., and Divakar, S. (1999) Bioinsecticide from swallowroot (Decalepis hamiltonii) Wight & Arn protects food grains against insect infestation. Current Science 44: 501–502.

Germinara, G.S., De Cristofaro, A., and Rotundo, G. (2008) Behavioral Responses of Adult Sitophilus granarius to Individual Cereal Volatiles. J Chem Ecol 34: 523–529.

Germinara, G.S., De Cristofaro, A., and Rotundo, G. (2015) Repellents effectively disrupt the olfactory orientation of Sitophilus granarius to wheat kernels. J Pest Sci 88: 675–684.

Ghahari, S., Alinezhad, H., Nematzadeh, G.A., Tajbakhsh, M., and Baharfar, R. (2017) Chemical Composition, Antioxidant and Biological Activities of the Essential Oil and Extract of the Seeds of Glycine max (Soybean) from North Iran. Curr Microbiol 74: 522–531.

Ghissing, U., Jayanthan, K., Bera, P., Bimolata, W., and Mitra, A. (2022) Targeted profiling and temporal expression of a few key genes revealed an apparent coordination among the metabolites contributing to the volatiles internal pool in Jasminum sambac (L.) Aiton flowers. Braz J Bot 45: 587–597.

Ghosh, S., Qureshi, A., and Purohit, H.J. (2019) D-Tryptophan governs biofilm formation rates and bacterial interaction in P. mendocina and S. aureus. J Biosci 44: 3.

Giordani, R., Hadef, Y., and Kaloustian, J. (2008) Compositions and antifungal activities of essential oils of some Algerian aromatic plants. Fitoterapia 79: 199–203.

Goel, D., Goel, R., Singh, V., Ali, M., Mallavarapu, G.R., and Kumar, S. (2007) Composition of the essential oil from the root of Artemisia annua. J Nat Med 61: 458–461.

Goetz-Schmidt, E.M. and Schreier, P. (1986) Neutral volatiles from blended endive (Cichorium endivia L.). J Agric Food Chem 34: 212–215.

Goufo, P., Duan, M., Wongpornchai, S., and Tang, X. (2010) Some factors affecting the concentration of the aroma compound 2-acetyl-1-pyrroline in two fragrant rice cultivars grown in South China. Front Agric China 4: 1–9.

Gough, A.J.E., Hamilton, J.G.C., Games, D.E., and Staddon, B.W. (1985) Multichemical defense of plant bugHotea gambiae (westwood) (Heteroptera: Scutelleridae): Sesquiterpenoids from abdominal gland in larvae. J Chem Ecol 11: 343–352.

Gross, J., Podsiadlowski, L., and Hilker, M. (2002) Antimicrobial Activity of Exocrine Glandular Secretion of Chrysomela Larvae. Journal of Chemical Ecology 28: 317–331.

Groux, R., Hilfiker, O., Gouhier-Darimont, C., Peñaflor, M.F.G.V., Erb, M., and Reymond, P. (2014) Role of Methyl Salicylate on Oviposition Deterrence in Arabidopsis thaliana. J Chem Ecol 40: 754–759.

Grove, J.F. (1981) Volatile compounds from the mycelium of the mushroom Agaricus bisporus. Phytochemistry 20: 2021–2022.

Grove, J.F. and Pople, M. (1981) Nitrogen-containing minor metabolic products of Beauveria bassiana. Phytochemistry 20: 815–816.

Grund, E., Denecke, B., and Eichenlaub, R. (1992) Naphthalene degradation via salicylate and gentisate by Rhodococcus sp. strain B4. Appl Environ Microbiol 58: 1874–1877.

Grzesiak, J., Woltyńska, A., Zdanowski, M.K., Górniak, D., Świątecki, A., Olech, M.A., and Aleksandrzak-Piekarczyk, T. (2021) Metabolic fingerprinting of the Antarctic cyanolichen Leptogium puberulum–associated bacterial community (Western Shore of Admiralty Bay, King George Island, Maritime Antarctica). Microb Ecol 82: 818–829.

Guerin, P.M. and Ryan, M.F. (1980) Insecticidal effect of trans-2-nonenal, a constituent of carrot root. Experientia 36: 1387–1388.

Güler, Z. and Gül, E. (2017) Volatile organic compounds in the aril juices and seeds from selected five pomegranate (Punica granatum L .) cultivars. International Journal of Food Properties 20: 281–293.

Gunawardena, N.E. and Bandumathie, M.K. (1993) Defensive secretion of rice bug,Leptocorisa oratorius fabricius, (Hemiptera: Coreidae): A unique chemical combination and its toxic, repellent, and alarm properties. J Chem Ecol 19: 851–861.

Guo, H., Hu, Z., Zhang, H., Hou, Z., and Min, W. (2019) Soil Microbial Metabolic Activity and Community Structure in Drip-Irrigated Calcareous Soil as Affected by Irrigation Water Salinity. Water Air Soil Pollut 230: 44.

Guo, X., Zhang, M., Cao, M., Zhang, W., Kang, Z., Xu, P., et al. (2018) d-2-Hydroxyglutarate dehydrogenase plays a dual role in l-serine biosynthesis and d-malate utilization in the bacterium Pseudomonas stutzeri. J Biol Chem 293: 15513–15523.

Gusakova, S.D. and Khomova, T.V. (1984) New oxo acids of the seed oil ofGaleopsis bifida. Chem Nat Compd 20: 266–270.

Gutiérrez, A., Caramelo, L., Prieto, A., Martínez, M.J., and Martínez, A.T. (1994) Anisaldehyde production and aryl-alcohol oxidase and dehydrogenase activities in ligninolytic fungi of the genus Pleurotus. Appl Environ Microbiol 60: 1783–1788.

Güven, K.C., Sezik, E., Kaleağasıoğlu, F., Erdugan, H., Coban, B., and Karakaş, E. (2013) Volatile Oils from Marine Macroalgae. In Natural Products. Ramawat, K.G. and Mérillon, J.-M. (eds). Berlin, Heidelberg: Springer Berlin Heidelberg, pp. 2883–2912.

Habibi, F., Ramezanian, A., Guillén, F., Serrano, M., and Valero, D. (2020) Blood oranges maintain bioactive compounds and nutritional quality by postharvest treatments with γ-aminobutyric acid, methyl jasmonate or methyl salicylate during cold storage. Food Chemistry 306: 125634.

Hagedorn, M.L. and Brown, S.M. (1991) The constituents of cascarilla oil (Croton eluteria bennett). Flavour Fragr J 6: 193–204.

Hamilton-Kemp, T.R., McCracken, C.T., Loughrin, J.H., Andersen, R.A., and Hildebrand, D.F. (1992) Effects of some natural volatile compounds on the pathogenic fungi Alternaria alternata and Botrytis cinerea. J Chem Ecol 18: 1083–1091.

Hammatt, N. (1994) Promotion by phloroglucinol of adventitious root formation in micropropagated shoots of adult wild cherry (Prunus avium L.). Plant Growth Regul 14: 127–132.

Hammond, C.T. and Mahlberg, P.G. (1994) Phloroglucinol glucoside as a natural constituent of Cannabis sativa. Phytochemistry 37: 755–756.

Hao, H., Zhao, X., Wang, Y., Zhang, Y., Xie, Z., Guo, Z., and Wang, R. (2017) Effects of gravel-sand mulching on soil bacterial community and metabolic capability in the semi-arid Loess Plateau, China. World J Microbiol Biotechnol 33: 209.

Hao, K., Ullah, H., Qin, X., Li, H., Li, F., and Guo, P. (2019) Effectiveness of Bacillus pumilus PDSLzg-1, an innovative Hydrocarbon-Degrading Bacterium conferring antifungal and plant growth-promoting function. 3 Biotech 9: 305.

Harraca, V., Ryne, C., and Ignell, R. (2010) Nymphs of the common bed bug (Cimex lectularius) produce anti-aphrodisiac defence against conspecific males. BMC Biol 8: 121.

Hassanpouraghdam, M.B. (2011) α-Pinene- and β-myrcene-rich volatile fruit oil of Cupressus arizonica Greene from northwest Iran. Natural Product Research 25: 634–639.

Hausch, B.J., Arpaia, M.L., Kawagoe, Z., Walse, S., and Obenland, D. (2020) Chemical Characterization of Two California-Grown Avocado Varieties (Persea americana Mill.) over the Harvest Season with an Emphasis on Sensory-Directed Flavor Analysis. J Agric Food Chem 68: 15301–15310.

Herdeiro, R.S., Pereira, M.D., Panek, A.D., and Eleutherio, E.C.A. (2006) Trehalose protects Saccharomyces cerevisiae from lipid peroxidation during oxidative stress. Biochimica et Biophysica Acta (BBA) - General Subjects 1760: 340–346.

Hippe, J. (1988) HPLC-analysis of the concentrations of free asparagine and glutamine in potato tubers grown with varying amounts of nitrogen. Potato Res 31: 535–540.

Hong, Y.-S., Martinez, A., Liger-Belair, G., Jeandet, P., Nuzillard, J.-M., and Cilindre, C. (2012) Metabolomics reveals simultaneous influences of plant defence system and fungal growth in Botrytis cinerea-infected Vitis vinifera cv. Chardonnay berries. Journal of Experimental Botany 63: 5773–5785.

Hopper, D.J., Chapman, P.J., and Dagley, S. (1970) Metabolism of l -Malate and d -Malate by a Species of Pseudomonas. J Bacteriol 104: 1197–1202.

Hounsome, N., Hounsome, B., Tomos, D., and Edwards-Jones, G. (2008) Plant Metabolites and Nutritional Quality of Vegetables. J Food Science 73: R48–R65.

Hountondji, F.C.C., Hanna, R., and Sabelis, M.W. (2006) Does Methyl Salicylate, A Component of Herbivore-induced Plant Odour, Promote Sporulation of the Mite-pathogenic Fungus Neozygites tanajoae? Exp Appl Acarol 39: 63–74.

Hsieh, P.-W., Chang, F.-R., Wu, C.-C., Li, C.-M., Wu, K.-Y., Chen, S.-L., et al. (2005) Longicalycinin A, a New Cytotoxic Cyclic Peptide from Dianthus superbus var. longicalycinus (Maxim.) Will. Chem Pharm Bull 53: 336–338.

Hsiung, Y.-C., Chen, Y.-A., Chen, S.-Y., Chi, W.-C., Lee, R.-H., Chiang, T.-Y., and Huang, H.-J. (2013) Volatilized myrcene inhibits growth and activates defense responses in rice roots. Acta Physiol Plant 35: 2475–2482.

Huang, Y., Zhao, K., Shen, X.-H., Jiang, C.-Y., and Liu, S.-J. (2008) Genetic and biochemical characterization of a 4-hydroxybenzoate hydroxylase from Corynebacterium glutamicum. Appl Microbiol Biotechnol 78: 75–83.

Huergo, L.F. and Dixon, R. (2015) The Emergence of 2-Oxoglutarate as a Master Regulator Metabolite. Microbiol Mol Biol Rev 79: 419–435.

Hung, R., Lee, S., Rodriguez-Saona, C., and Bennett, J.W. (2014) Common gas phase molecules from fungi affect seed germination and plant health in Arabidopsis thaliana. AMB Expr 4: 53.

Huong, L.T., Thang, T.D., and Ogunwande, I.A. (2015) Chemical constituents of essential oils from the leaves, stems, roots and fruits of Alpinia polyantha. Nat Prod Commun 10: 367–368.

Hussain, M., Reigosa, M., and Muscolo, A. (2018) Carbon (δ13C) and Nitrogen (δ15N) Stable Isotope Composition Provide New Insights into Phenotypic Plasticity in Broad Leaf Weed Rumex acetosa under Allelochemical Stress. Molecules 23: 2449.

Hussain, M.S., Musharraf, S.G., Bhanger, M.I., and Malik, M.I. (2020) Salicylaldehyde derivative of nano-chitosan as an efficient adsorbent for lead(II), copper(II), and cadmium(II) ions. International Journal of Biological Macromolecules 147: 643–652.

Hwang, E., Ngo, H.T.T., Park, B., Seo, S.-A., Yang, J.-E., and Yi, T.-H. (2017) Myrcene, an Aromatic Volatile Compound, Ameliorates Human Skin Extrinsic Aging via Regulation of MMPs Production. Am J Chin Med 45: 1113–1124.

Iossifova, T., Kostova, I., and Evstatieva, L.N. (1997) Secoiridoids and hydroxycoumarins in Bulgarian Fraxinus species. Biochemical Systematics and Ecology 25: 271–274.

Iwabuchi, H., Yoshikura, M., Obata, S., and Kamisako, W. (1984) Studies on the Aroma Constituents of Crude Drugs. I. On the Aroma Constituents of Ginseng Radix. YAKUGAKU ZASSHI 104: 951–958.

Jabalpurwala, F.A., Smoot, J.M., and Rouseff, R.L. (2009) A comparison of Citrus blossom volatiles. Phytochemistry 70: 1428–1434.

Jeleń, H.H., Mildner-Szkudlarz, S., Jasińska, I., and Wąsowicz, E. (2007) A Headspace–SPME–MS Method for Monitoring Rapeseed Oil Autoxidation. J Amer Oil Chem Soc 84: 509–517.

Jiang, Y., Ghisalberti, E.L., and Ridsdill-Smith, T.J. (1996) Correlation of 1-octen-3-one with antixenotic resistance in subterranean clover cotyledons to red-legged earth mite, Halotydeus destructor (Acarina: Penthaleidae). J Chem Ecol 22: 369–382.

Jin, X., Wu, F., and Zhou, X. (2020) Different toxic effects of ferulic and p-hydroxybenzoic acids on cucumber seedling growth were related to their different influences on rhizosphere microbial composition. Biol Fertil Soils 56: 125–136.

Jo, K., Kim, H., Choi, H.-S., Lee, S.-S., Bang, M.-H., and Suh, H.J. (2018) Isolation of a sleep-promoting compound from Polygonatum sibiricum rhizome. Food Sci Biotechnol 27: 1833–1842.

Jørgensen, U., Hansen, M., Christensen, L.P., Jensen, K., and Kaack, K. (2000) Olfactory and Quantitative Analysis of Aroma Compounds in Elder Flower ( Sambucus nigra L.) Drink Processed from Five Cultivars. J Agric Food Chem 48: 2376–2383.

Kacem, N., Hay, A.-E., Marston, A., Zellagui, A., Rhouati, S., and Hostettmann, K. (2012) Antioxidant compounds from Algerian Convolvulus tricolor (Convolvulaceae) seed husks. Nat Prod Commun 7: 873–874.

Kallio, H. and Linko, R.R. (1973) Volatile monocarbonyl compounds of arctic bramble (Rubus arcticus L.) at various stages of ripeness. Z Lebensm Unters Forch 153: 23–30.

Kameoka, H., Kubo, K., and Miyazawa, M. (1991) Volatile flavor components of malabar-nightshade (Basella rubra L.). Journal of Food Composition and Analysis 4: 315–321.

Kameoka, H., Murakami, K., and Miyazawa, M. (1994) Composition of the Bark Oil of Magnolia obovata Thunb. Journal of Essential Oil Research 6: 555–560.

Kan, K., Chen, J., Kawamura, S., and Koseki, S. (2018) Characteristics of d-Tryptophan as an Antibacterial Agent: Effect of Sodium Chloride Concentration and Temperature on Escherichia coli Growth Inhibition. Journal of Food Protection 81: 25–30.

Kanse, O.S., Whitelaw-Weckert, M., Kadam, T.A., and Bhosale, H.J. (2015) Phosphate solubilization by stress-tolerant soil fungus Talaromyces funiculosus SLS8 isolated from the Neem rhizosphere. Ann Microbiol 65: 85–93.

Karagoz, S.G., Yilmazer, M., Ozkan, G., Carbonell-Barrachina, Á.A., Kiralan, M., and Ramadan, M.F. (2017) Effect of cultivar and harvest time on C6 and C5 volatile compounds of Turkish olive oils. Eur Food Res Technol 243: 1193–1200.

Karak, S., Nag, G., and De, B. (2017) Metabolic profile and β-glucuronidase inhibitory property of three species of Swertia. Revista Brasileira de Farmacognosia 27: 105–111.

Karmakar, A., Mitra, P., Koner, A., Das, S., and Barik, A. (2020) Fruit Volatiles of Creeping Cucumber (Solena amplexicaulis) Attract a Generalist Insect Herbivore. J Chem Ecol 46: 275–287.

Karthik, R., Manigandan, V., Sheeba, R., Saravanan, R., and Rajesh, P.R. (2016) Structural characterization and comparative biomedical properties of phloroglucinol from Indian brown seaweeds. J Appl Phycol 28: 3561–3573.

Kashyap, P., Ram, H., Shukla, S.D., and Kumar, S. (2020) Scopoletin: Antiamyloidogenic, Anticholinesterase, and Neuroprotective Potential of a Natural Compound Present in Argyreia speciosa Roots by In Vitro and In Silico Study. J Exp Neurosci 15: 263310552093769.

Kaur, R., Macleod, J., Foley, W., and Nayudu, M. (2006) Gluconic acid: An antifungal agent produced by Pseudomonas species in biological control of take-all. Phytochemistry 67: 595–604.

Kaur, T., Devi, R., Kour, D., Yadav, A., Yadav, A.N., Dikilitas, M., et al. (2021) Plant growth promoting soil microbiomes and their potential implications for agricultural and environmental sustainability. Biologia 76: 2687–2709.

Keman, D. and Soyer, F. (2019) Antibiotic-Resistant Staphylococcus aureus Does Not Develop Resistance to Vanillic Acid and 2-Hydroxycinnamic Acid after Continuous Exposure in Vitro. ACS Omega 4: 15393–15400.

Kemp, T.R., Knavel, D.E., and Stoltz, L.P. (1974) Identification of some volatile compounds from cucumber. J Agric Food Chem 22: 717–718.

Khakdan, F., Govahi, M., Mohebi, Z., and Ranjbar, M. (2021) Water deficit stress responses of monoterpenes and sesquiterpenes in different Iranian cultivars of basil. Physiologia Plantarum 173: 896–910.

Khaoua, S., Lebrihi, A., Germain, P., and Lefebvre, G. (1991) Cephamycin C biosynthesis in Streptomyces cattleya: nitrogen source regulation. Appl Microbiol Biotechnol 35:.

Kim, E. and Park, I.-K. (2012) Fumigant Antifungal Activity of Myrtaceae Essential Oils and Constituents from Leptospermum petersonii against Three Aspergillus Species. Molecules 17: 10459–10469.

Kim, E.-M., Eom, J.-H., Um, Y., Kim, Y., and Woo, H.M. (2015) Microbial Synthesis of Myrcene by Metabolically Engineered Escherichia coli. J Agric Food Chem 63: 4606–4612.

Kim, H.J., Yu, Y.G., Park, H., and Lee, Y.S. (2002) HIV gp41 binding phenolic components from Fraxinus sieboldiana var. angustata. Planta Med 68: 1034–1036.

Kim, J.H., Campbell, B.C., Mahoney, N., Chan, K.L., and Molyneux, R.J. (2011) Chemosensitization of Aflatoxigenic Fungi to Antimycin A and Strobilurin Using Salicylaldehyde, a Volatile Natural Compound Targeting Cellular Antioxidation System. Mycopathologia 171: 291–298.

Kim, Y., Hirai, S., Goto, T., Ohyane, C., Takahashi, H., Tsugane, T., et al. (2012) Potent PPARα Activator Derived from Tomato Juice, 13-oxo-9,11-Octadecadienoic Acid, Decreases Plasma and Hepatic Triglyceride in Obese Diabetic Mice. PLoS ONE 7: e31317.

Kimura, H. and Yokota, K. (2004) Characterization of Metabolic Pathway of Linoleic Acid 9-Hydroperoxide in Cytosolic Fraction of Potato Tubers and Identification of Reaction Products. ABAB 118: 115–132.

Kino, K., Sato, M., Yoneyama, M., and Kirimura, K. (2007) Synthesis of dl-tryptophan by modified broad specificity amino acid racemase from Pseudomonas putida IFO 12996. Appl Microbiol Biotechnol 73: 1299–1305.

Kiralan, M., Çalik, G., Kiralan, S., and Ramadan, M.F. (2018) Monitoring stability and volatile oxidation compounds of cold-pressed flax seed, grape seed and black cumin seed oils upon photo-oxidation. Food Measure 12: 616–621.

Koch, M. and Gregson, R.P. (1984) Brominated phlorethols and nonhalogenated phlorotannins from the brown alga Cystophora congesta. Phytochemistry 23: 2633–2637.

Kong, L.D., Abliz, Z., Zhou, C.X., Li, L.J., Cheng, C.H.K., and Tan, R.X. (2001) Glycosides and xanthine oxidase inhibitors from Conyza bonariensis. Phytochemistry 58: 645–651.

Konovalova, O.A., Rybalko, K.S., and Shretev, A.I. (1976) Coumarins of species of the genus Artemisia. Chem Nat Compd 12: 86–86.

Koorbanally, N.A., Mulholland, D.A., and Crouch, N.R. (2000) Isolation of Isovanillin from Aromatic Roots of the Medicinal African Liane, M.whitei. Journal of Herbs, Spices & Medicinal Plants 7: 37–43.

Kostikova, V.A. and Shaldaeva, T.M. (2017) The Antioxidant Activity of the Russian Far East Representatives of the Spiraea L. (Rosaceae Juss.) Genus. Russ J Bioorg Chem 43: 790–794.

Krzaczek, T., Bogucka-Kocka, A., and Śnieżko, R. (2014) The phenolic acids of some species of the Oenothera L. genus. Acta Soc Bot Pol 64: 41–44.

Ksouda, G., Sellimi, S., Merlier, F., Falcimaigne-cordin, A., Thomasset, B., Nasri, M., and Hajji, M. (2019) Composition, antibacterial and antioxidant activities of Pimpinella saxifraga essential oil and application to cheese preservation as coating additive. Food Chemistry 288: 47–56.

Kubo, I. and Kinst-Hori, I. (1999) 2-Hydroxy-4-methoxybenzaldehyde: A Potent Tyrosinase Inhibitor from African Medicinal Plants. Planta med 65: 019–022.

Kubo, J., Lee, J.R., and Kubo, I. (1999) Anti-Helicobacter pylori Agents from the Cashew Apple. J Agric Food Chem 47: 533–537.

Küçükbay, F.Z., Kuyumcu, E., Bilenler, T., and Yıldız, B. (2012) Chemical composition and antimicrobial activity of essential oil of Achillea cretica L. (Asteraceae) from Turkey. Natural Product Research 26: 1668–1675.

Kudisi, D., Lu, X., Zheng, C., Wang, Y., Cai, T., Li, W., et al. (2022) Long-term performance, membrane fouling behaviors and microbial community in a hollow fiber anaerobic membrane bioreactor (HF-AnMBR) treating synthetic terephthalic acid-containing wastewater. Journal of Hazardous Materials 424: 127458.

Kumari, Shailja, Kumari, Swati, Attri, C., Sharma, R., Kulshreshtha, S., Benali, T., et al. (2022) GC-MS Analysis, Antioxidant and Antifungal Studies of Different Extracts of Chaetomium globosum Isolated from Urginea indica. BioMed Research International 2022: 1–12.

Kuo, S.-Y., Hsieh, T.-J., Wang, Y.-D., Lo, W.-L., Hsui, Y.-R., and Chen, C.-Y. (2008) Cytotoxic Constituents from the Leaves of Cinnamomum subavenium. Chem Pharm Bull 56: 97–101.

Kurobayashi, Y., Kouno, E., Fujita, A., Morimitsu, Y., and Kubota, K. (2006) Potent Odorants Characterize the Aroma Quality of Leaves and Stalks in Raw and Boiled Celery. Bioscience, Biotechnology, and Biochemistry 70: 958–965.

Kurobayashi, Y., Sakakibara, H., Yanai, T., Yajima, I., and Hayashi, K. (1991) Volatile Flavor Compounds of Myoga (Zingiber Mioga). Agricultural and Biological Chemistry 55: 1655–1657.

Kurosawa, K., Laser, J., and Sinskey, A.J. (2015) Tolerance and adaptive evolution of triacylglycerol-producing Rhodococcus opacus to lignocellulose-derived inhibitors. Biotechnol Biofuels 8: 76.

Lamine, M., Hamdi, Z., Rahali, F.Z., Mliki, A., Varzakas, T., and Gargouri, M. (2023) Non-targeting metabolite profiling and chemometric approaches for the discrimination and authentication analyses of whole-wheat flours from Tunisian durum wheat landraces (Triticum turgidum ssp. durum). Food Measure 17: 1889–1898.

Lan, J., Hu, M., Gao, C., Alshawabkeh, A., and Gu, A.Z. (2015) Toxicity Assessment of 4-Methyl-1-cyclohexanemethanol and Its Metabolites in Response to a Recent Chemical Spill in West Virginia, USA. Environ Sci Technol 49: 6284–6293.

Lanciotti, R., Gianotti, A., Patrignani, F., Belletti, N., Guerzoni, M.E., and Gardini, F. (2004) Use of natural aroma compounds to improve shelf-life and safety of minimally processed fruits. Trends in Food Science & Technology 15: 201–208.

Lawo, N.C., Weingart, G.J.F., Schuhmacher, R., and Forneck, A. (2011) The volatile metabolome of grapevine roots: first insights into the metabolic response upon phylloxera attack. Plant Physiol Biochem 49: 1059–1063.

Laznik, Ž. and Trdan, S. (2016) Attraction Behaviors of Entomopathogenic Nematodes (Steinernematidae and Heterorhabditidae) to Synthetic Volatiles Emitted by Insect Damaged Potato Tubers. J Chem Ecol 42: 314–322.

Leal, W., Hasegawa, M., Sawada, M., Ono, M., and Tada, S. (1996) Scarab beetle Anomala albopilosa utilizes a more complex sex pheromone system than a similar species A. cuprea. J Chem Ecol 22: 2001–2010.

Lee, A.Y., Kim, H.S., Choi, G., Moon, B.C., Chun, J.M., and Kim, H.K. (2014) Optimization of Ultrasonic-Assisted Extraction of Active Compounds from the Fruit of Star Anise by Using Response Surface Methodology. Food Anal Methods 7: 1661–1670.

Lee, I., Johnson, L.A., and Hammond, E.G. (1995) Use of branched-chain esters to reduce the crystallization temperature of biodiesel. J Am Oil Chem Soc 72: 1155–1160.

Lee, J.-H., Lee, K., Lee, D.H., Shin, S.Y., Yong, Y., and Lee, Y.H. (2015) Anti-invasive effect of β-myrcene, a component of the essential oil from Pinus koraiensis cones, in metastatic MDA-MB-231 human breast cancer cells. J Korean Soc Appl Biol Chem 58: 563–569.

Lee, S., Hung, R., Schink, A., Mauro, J., and Bennett, J.W. (2014) Arabidopsis thaliana for testing the phytotoxicity of volatile organic compounds. Plant Growth Regul 74: 177–186.

Lee, S.-H., Ding, Y., Yan, X.T., Kim, Y.-H., and Jang, H.-D. (2013) Scopoletin and Scopolin Isolated from Artemisia iwayomogi Suppress Differentiation of Osteoclastic Macrophage RAW 264.7 Cells by Scavenging Reactive Oxygen Species. J Nat Prod 76: 615–620.

Legendre, F., MacLean, A., Appanna, V.P., and Appanna, V.D. (2020) Biochemical pathways to α-ketoglutarate, a multi-faceted metabolite. World J Microbiol Biotechnol 36: 123.

Legendre, F., MacLean, A., Tharmalingam, S., and Appanna, V.D. (2022) A Metabolic Network Mediating the Cycling of Succinate, a Product of ROS Detoxification into α-Ketoglutarate, an Antioxidant. Antioxidants (Basel) 11: 560.

León, J. (2022) Protein Tyrosine Nitration in Plant Nitric Oxide Signaling. Front Plant Sci 13: 859374.

Lerat, S., Babana, A.H., El Oirdi, M., El Hadrami, A., Daayf, F., Beaudoin, N., et al. (2009) Streptomyces scabiei and its toxin thaxtomin A induce scopoletin biosynthesis in tobacco and Arabidopsis thaliana. Plant Cell Rep 28: 1895–1903.

Leyte-Lugo, M., Richomme, P., Poupard, P., and Peña-Rodriguez, L.M. (2020) Identification and Quantification of a Phytotoxic Metabolite from Alternaria dauci. Molecules 25: 4003.

Li, H.-H., Inoue, M., Nishimura, H., Mizutani, J., and Tsuzuki, E. (1993) Interactions oftrans-cinnamic acid, its related phenolic allelochemicals, and abscisic acid in seedling growth and seed germination of lettuce. J Chem Ecol 19: 1775–1787.

Li, J., Fu, Y., Bao, X., Li, H., Zuo, J., Zhang, M., and Wang, J. (2020) Comparison and analysis of tomato flavor compounds using different extraction methods. Food Measure 14: 465–475.

Li, J., Huang, S.-Y., Deng, Q., Li, G., Su, G., Liu, J., and David Wang, H.-M. (2020) Extraction and characterization of phenolic compounds with antioxidant and antimicrobial activities from pickled radish. Food and Chemical Toxicology 136: 111050.

Li, M., Chen, S., Li, J., and Ji, Z. (2014) Propanol Addition Improves Natamycin Biosynthesis of Streptomyces natalensis. Appl Biochem Biotechnol 172: 3424–3432.

Li, S.-F., Zhang, S.-B., Lv, Y.-Y., Zhai, H.-C., Li, N., Hu, Y.-S., and Cai, J.-P. (2021) Metabolomic analyses revealed multifaceted effects of hexanal on Aspergillus flavus growth. Appl Microbiol Biotechnol 105: 3745–3757.

Li, S.-F., Zhang, S.-B., Zhai, H.-C., Lv, Y.-Y., Hu, Y.-S., and Cai, J.-P. (2021) Hexanal induces early apoptosis of Aspergillus flavus conidia by disrupting mitochondrial function and expression of key genes. Appl Microbiol Biotechnol 105: 6871–6886.

Li, X., Zhang, Y., Zeng, X., Yang, L., and Deng, Y. (2011) Chemical profiling of bioactive constituents in Sarcandra glabra and its preparations using ultra-high-pressure liquid chromatography coupled with LTQ Orbitrap mass spectrometry: Constituents in Sarcandra glabra by UPLC LTQ Orbitrap. Rapid Commun Mass Spectrom 25: 2439–2447.

Li, Z., Dong, F., Sun, Y., Sun, Z., Song, X., Dong, Y., et al. (2022) Qualitative and Quantitative Analysis of Six Fatty Acid Amides in 11 Edible Vegetable Oils Using Liquid Chromatography–Mass Spectrometry. Front Nutr 9: 857858.

Liarzi, O., Benichis, M., Gamliel, A., and Ezra, D. (2020) trans ‐2‐Octenal, a single compound of a fungal origin, controls Sclerotium rolfsii , both in vitro and in soil. Pest Manag Sci 76: 2068–2071.

Lija-Escaline, J., Senthil-Nathan, S., Thanigaivel, A., Pradeepa, V., Vasantha-Srinivasan, P., Ponsankar, A., et al. (2015) Physiological and biochemical effects of botanical extract from Piper nigrum Linn (Piperaceae) against the dengue vector Aedes aegypti Liston (Diptera: Culicidae). Parasitol Res 114: 4239–4249.

Lim, T.K. (2012) Citrullus lanatus. In Edible Medicinal And Non-Medicinal Plants. Dordrecht: Springer Netherlands, pp. 179–190.

Lin, C.-L., Kang, Y.-F., Li, W.-J., Li, H.-T., Li, C.-T., and Chen, C.-Y. (2016) Secondary Metabolites from the Unripe Fruits of Capsicum annuum var. conoides. Chem Nat Compd 52: 1145–1146.

Lin, J.-T. and Liu, W.-H. (2006) ο -Orsellinaldehyde from the Submerged Culture of the Edible Mushroom Grifola frondosa Exhibits Selective Cytotoxic Effect Against Hep 3B Cells Through Apoptosis. J Agric Food Chem 54: 7564–7569.

Lin, S., Liu, M., Wang, S., Li, S., Yang, Y., and Shi, J. (2008) Coumarins from branch of Fraxinus sieboldiana and their antioxidative activity. Zhongguo Zhong Yao Za Zhi 33: 1708–1710.

Lin, W.-Y., Teng, C.-M., Tsai, I.-L., and Chen, I.-S. (2000) Anti-platelet aggregation constituents from Gynura elliptica. Phytochemistry 53: 833–836.

Lin, Y., Enyoh, C.E., Wang, Q., Lu, S., Zhang, W., Xiao, K., et al. (2022) Novel Approaches for Inhibiting the Indoor Allergen Der f 2 Excreted from House Dust Mites by Todomatsu Oil Produced from Woodland Residues. IJERPH 19: 10881.

Linko, R.R., Kallio, H., Pyysalo, T., and Rainio, K. (1978) Volatile monocarbonyl compounds of carrot roots at various stages of maturity. Z Lebensm Unters Forch 166: 208–211.

Liu, C., Zheng, P., Wang, H., Wei, Y., Wang, C., and Hao, S. (2023) Design and Synthesis of Scopoletin Sulfonate Derivatives as Potential Insecticidal Agents. Molecules 28: 530.

Liu, J., Liu, Y., Jia, M., Kang, X., Wang, S., Sun, H., et al. (2021) Association of enriched metabolites profile with the corresponding volatile characteristics induced by rice yellowing process. Food Chemistry 349: 129173.

Liu, M., Liu, J., Jiang, C., Wu, M., Song, R., Gui, R., et al. (2017) Improved nutrient status affects soil microbial biomass, respiration, and functional diversity in a Lei bamboo plantation under intensive management. J Soils Sediments 17: 917–926.

Liu, T., Li, X.-G., Wang, J.-Y., Liu, D.-L., and Wei, Y.-J. (2019) Time-resolved fluorescence and chemometrics-assisted excitation–emission fluorescence for qualitative and quantitative analysis of scopoletin and scopolin in Erycibe obtusifolia Benth. Spectrochimica Acta Part A: Molecular and Biomolecular Spectroscopy 219: 96–103.

Lomascolo, A., Navarro, D., Delattre, M., Asther, M., Lesage-Meessen, L., and Estrada Alvarado, I. (2001) Evidence of a new biotransformation pathway of p-coumaric acid into p-hydroxybenzaldehyde in Pycnoporus cinnabarinus. Applied Microbiology and Biotechnology 57: 725–730.

Lotfy, S., Javelle, F., and Negrel, J. (1995) Formation of ω-Feruloyloxypalmitic Acid and Tetradecyl Ferulate in Enzymic Extracts of Wound-Healing Potato Tuber Discs (Solanum tuberosum L.). In Plant Lipid Metabolism. Kader, J.-C. and Mazliak, P. (eds). Dordrecht: Springer Netherlands, pp. 456–458.

Lukas, H., Reimann, J., Kim, O.B., Grimpo, J., and Unden, G. (2010) Regulation of aerobic and anaerobic D-malate metabolism of Escherichia coli by the LysR-type regulator DmlR (YeaT). J Bacteriol 192: 2503–2511.

Lunn, J.E., Delorge, I., Figueroa, C.M., Van Dijck, P., and Stitt, M. (2014) Trehalose metabolism in plants. Plant J 79: 544–567.

Lwande, W. and Bentley, M.D. (1987) Volatiles of Sorghum bicolor Seedlings. J Nat Prod 50: 950–952.

Ma, Z. and Zhao, Z. (2008) Studies on chemical constituents from stem barks of Fraxinus paxiana. Zhongguo Zhong Yao Za Zhi 33: 1990–1993.

MacEwen, E.G. (1986) Anti-tumor evaluation of benzaldehyde in the dog and cat. Am J Vet Res 47: 451–452.

MacLeod, A.J., MacLeod, G., and Subramanian, G. (1988) Volatile aroma constituents of celery. Phytochemistry 27: 373–375.

MacLeod, A.J., Pieris, N.M., and de Troconis, N.G. (1982) Aroma volatiles of Cynara scolymus and Helianthus tuberosus. Phytochemistry 21: 1647–1651.

Maggi, F., Nicoletti, M., Petitto, V., Sagratini, G., Papa, F., and Vittori, S. (2011) Solid-Phase Microextraction (SPME) Analysis of Six Italian Populations of Ephedra nebrodensis Tineo ex Guss. subsp. nebrodensis. Chemistry & Biodiversity 8: 95–114.

Mahmoud, M.M., Abdel-Razek, A.S., Frese, M., Soliman, H.S.M., Sewald, N., and Shaaban, M. (2018) 3,4-Dihydro-quinolin-2-one derivatives from extremophilic Streptomyces sp. LGE21. Med Chem Res 27: 1834–1842.

Maile, R., Jungnickel, H., Morgan, E.D., Ito, F., and Billen, J. (2000) Secretion of Venom and Dufour Glands in the Ant Leptogenys diminuta. Journal of Chemical Ecology 26: 2497–2506.

Majetic, C.J., Raguso, R.A., Tonsor, S.J., and Ashman, T.-L. (2007) Flower color–flower scent associations in polymorphic Hesperis matronalis (Brassicaceae). Phytochemistry 68: 865–874.

Mamkulathil Devasia, R., Altaf, M., Fahad Alrefaei, A., and Manoharadas, S. (2021) Enhanced production of camptothecin by immobilized callus of Ophiorrhiza mungos and a bioinformatic insight into its potential antiviral effect against SARS-CoV-2. Journal of King Saud University - Science 33: 101344.

Manaharan, T., Ming, C.H., and Palanisamy, U.D. (2013) Syzygium aqueum leaf extract and its bioactive compounds enhances pre-adipocyte differentiation and 2-NBDG uptake in 3T3-L1 cells. Food Chemistry 136: 354–363.

Martínez-Luque, M., Castillo, F., and Blasco, R. (2001) Assimilation of D -Malate by Rhodobacter capsulatus E1F1. Current Microbiology 43: 154–157.

Martini, A., Botti, F., Galletti, G., Bocchini, P., Bazzocchi, G., Baronio, P., and Burgio, G. (2010) The Influence of Pine Volatile Compounds on the Olfactory Response by Neodiprion sertifer (Geoffroy) Females. J Chem Ecol 36: 1114–1121.

Masood, F., Ahmad, S., and Malik, A. (2022) Role of Rhizobacterial Bacilli in Zinc Solubilization. In Microbial Biofertilizers and Micronutrient Availability. Khan, S.T. and Malik, A. (eds). Cham: Springer International Publishing, pp. 361–377.

Matsunaga, I., Sumimoto, T., Ueda, A., Kusunose, E., and Ichihara, K. (2000) Fatty acid-specific, regiospecific, and stereospecific hydroxylation by cytochrome P450 (CYP152B1) from Sphingomonas paucimobilis: Substrate structure required for α-hydroxylation. Lipids 35: 365–371.

Matthews, R.F., Scanlan, R.A., and Libbey, L.M. (1971) Autoxidation products of 2,4-decadienal. J Am Oil Chem Soc 48: 745–747.

McAuslane, H.J., Vinson, S.B., and Williams, H.J. (1990) Change in mandibular and mesosomal gland contents of maleXylocopa micans (Hymenoptera: Anthophoridae) associated with mating system. J Chem Ecol 16: 1877–1885.

Messaoud, C., Chograni, H., and Boussaid, M. (2012) Chemical composition and antioxidant activities of essential oils and methanol extracts of three wild Lavandula L. species. Natural Product Research 26: 1976–1984.

Miao, Z., Zhang, L., Gu, M., Huang, J., Wang, X., Yan, J., et al. (2021) Preparation of Fraxetin Long Circulating Liposome and Its Anti-enteritis Effect. AAPS PharmSciTech 22: 110.

Miles, S. (2007) Methyl Salicylate. In xPharm: The Comprehensive Pharmacology Reference. Elsevier, pp. 1–6.

Miller, R.R., Newhook, R., and Poole, A. (1994) Styrene Production, Use, and Human Exposure. Critical Reviews in Toxicology 24: S1–S10.

Miri, S., Rasooli, A., Brar, S.K., Rouissi, T., and Martel, R. (2022) Biodegradation of p-xylene—a comparison of three psychrophilic Pseudomonas strains through the lens of gene expression. Environ Sci Pollut Res 29: 21465–21479.

Mitra, S., Karmakar, A., Mukherjee, A., and Barik, A. (2017) The Role of Leaf Volatiles of Ludwigia octovalvis (Jacq.) Raven in the Attraction of Altica cyanea (Weber) (Coleoptera: Chrysomelidae). J Chem Ecol 43: 679–692.

Miyama, M. and Nakayama, K. (1993) D-malic acid production from DL-malic acid by enantiospecific assimilation with Acinetobacter lwofii. Biotechnol Lett 15: 23–28.

Miyazawa, M. and Kawata, J. (2006) Identification of the main aroma compounds in dried seeds of Brassica hirta. J Nat Med 60: 89–92.

Mogana, R., Teng-Jin, K., and Wiart, C. (2013) Anti-Inflammatory, Anticholinesterase, and Antioxidant Potential of Scopoletin Isolated from Canarium patentinervium Miq. (Burseraceae Kunth). Evid Based Complement Alternat Med 2013: 734824.

Mohammad, B.T., Wright, P.C., and Bustard, M.T. (2006) Bioconversion of isopropanol by a solvent tolerant Sphingobacterium mizutae strain. J Ind Microbiol Biotechnol 33: 975–983.

Mohd Din, A.R.J., Rosli, M.A., Mohamad Azam, Z., Othman, N.Z., and Sarmidi, M.R. (2020) Paenibacillus polymyxa Role Involved in Phosphate Solubilization and Growth Promotion of Zea mays Under Abiotic Stress Condition. Proc Natl Acad Sci, India, Sect B Biol Sci 90: 63–71.

Morais, J., Cabral, L., Costa, W., Uhlmann, L., Lima, M., Noronha, M., et al. (2022) Chemical and volatile composition, and microbial communities in edible purple flowers (Torenia fournieri F. Lind.) cultivated in different organic systems. Food Research International 162: 111973.

Morikawa, T., Tao, J., Ueda, K., Matsuda, H., and Yoshikawa, M. (2003) Medicinal Foodstuffs. XXXI. Structures of New Aromatic Constituents and Inhibitors of Degranulation in RBL-2H3 Cells from a Japanese Folk Medicine, the Stem Bark of Acer nikoense. Chem Pharm Bull 51: 62–67.

Mozuraitis, R. (2002) (-)-Germacrene D Increases Attraction and Oviposition by the Tobacco Budworm Moth Heliothis virescens. Chemical Senses 27: 505–509.

Muceneeki, R.S., Amorim, C.M., Cesca, T.G., Biavatti, M.W., and Bresolin, T.M.B. (2009) A Simple and Validated LC Method for the Simultaneous Determination of Three Compounds in Mikania laevigata Extracts. Chroma 69: 219–223.

Mudd, A., Fisher, R.C., and Smith, M.C. (1982) Volatile hydrocarbons in the dufour’s gland of the parasite Nemeritis canescens (Grav.) (Hymenoptera; ichneumonidae). J Chem Ecol 8: 1035–1042.

Mueller, G.P. and Driscoll, W.J. (2009) Chapter 3 Biosynthesis of Oleamide. In Vitamins & Hormones. Elsevier, pp. 55–78.

Mustafa, A.M., Caprioli, G., Maggi, F., Vittori, S., and Sagratini, G. (2016) Comparative Analysis of the Volatile Profiles from Wild, Cultivated, and Commercial Roots of Gentiana lutea L. by Headspace Solid Phase Microextraction (HS–SPME) Coupled to Gas Chromatography Mass Spectrometry (GC–MS). Food Anal Methods 9: 311–321.

Nakamura, Y., Suganuma, E., Matsuo, T., Okamoto, S., Sato, K., and Ohtsuki, K. (1999) 2,4-Nonadienal and Benzaldehyde Bioantimutagens in Fushimi Sweet Pepper (Fushimi-Togarashi). J Agric Food Chem 47: 544–549.

Nakayachi, T., Yasumoto, E., Nakano, K., Morshed, S.R.M., Hashimoto, K., Kikuchi, H., et al. (2004) Structure-activity relationships of alpha, beta-unsaturated ketones as assessed by their cytotoxicity against oral tumor cells. Anticancer Res 24: 737–742.

Nakayama, K. and Ushijima, M. (1993) D-malic acid production from maleic acid using microorganism: Screening of microorganism. Biotechnol Lett 15: 271–276.

Narantuyaa, S., Batsurén, D., Batirov, É.Kh., and Malikov, V.M. (1986) Chemical study of plants of the Mongolian flora coumarins of Salsola laricifolia. Chem Nat Compd 22: 228–229.

Nascimento, I. (1999) 2,3-Dihydrobenzofuran neolignans from Aristolochia pubescens. Phytochemistry 52: 345–350.

Ndjonka, D., Bergmann, B., Agyare, C., Zimbres, F.M., Lüersen, K., Hensel, A., et al. (2012) In vitro activity of extracts and isolated polyphenols from West African medicinal plants against Plasmodium falciparum. Parasitol Res 111: 827–834.

Neetu, N., Sharma, M., Mahto, J.K., and Kumar, P. (2020) Biophysical and In-Silico Studies of Phytochemicals Targeting Chorismate Synthase from Drug-Resistant Moraxella Catarrhalis. Protein J 39: 449–460.

Newhart, A.T. and Mumma, R.O. (1979) Defensive secretions of three species of Acilius (Coleoptera: Dytiscidae) and their seasonal variations as determined by high-pressure liquid chromatography. J Chem Ecol 5: 643–652.

Ng, Z.R., Takahashi, K., and Liu, Z. (2013) Isolation, characterization and evaluation of hyper 2-propanol producing bacteria from Singapore environment. World J Microbiol Biotechnol 29: 1059–1065.

Ngoc, T.M., Khoi, N.M., Ha, D.T., Nhiem, N.X., Tai, B.H., Don, D.V., et al. (2012) Xanthine oxidase inhibitory activity of constituents of Cinnamomum cassia twigs. Bioorganic & Medicinal Chemistry Letters 22: 4625–4628.

Ngoc, T.M., Nhiem, N.X., Khoi, N.M., Son, D.C., Hung, T.V., and Van Kiem, P. (2014) A new coumarin and cytotoxic activities of constituents from Cinnamomum cassia. Nat Prod Commun 9: 487–488.

Nicollier, G.F., Pope, D.F., and Thompson, A.C. (1983) Biological activity of dhurrin and other compounds from Johnson grass (Sorghum halepense). J Agric Food Chem 31: 744–748.

Noge, K., Prudic, K.L., and Becerra, J.X. (2012) Defensive Roles of (E)-2-Alkenals and Related Compounds in Heteroptera. J Chem Ecol 38: 1050–1056.

Noleau, I., Richard, H., and Peyroux, A.-S. (1991) Volatile Compounds in Leek and Asafoetida. Journal of Essential Oil Research 3: 241–256.

Nouioui, I., Ghodhbane-Gtari, F., del Carmen Montero-Calasanz, M., Rohde, M., Tisa, L.S., Gtari, M., and Klenk, H.-P. (2017) Frankia inefficax sp. nov., an actinobacterial endophyte inducing ineffective, non nitrogen-fixing, root nodules on its actinorhizal host plants. Antonie van Leeuwenhoek 110: 313–320.

Nowak, R. and Kawka, S. (2014) Phenolic acids in leaves of Secamone afzelii (Rhoem.) Schult. (Asclepiadaceae). Acta Soc Bot Pol 67: 243–245.

Ntalli, N., Oplos, C., Michailidis, M., Thanasenaris, A., Kontea, D., Caboni, P., et al. (2016) Strong synergistic activity and egg hatch inhibition by (E,E)-2,4-decadienal and (E)-2-decenal in Meloidogyne species. J Pest Sci 89: 565–579.

Okamoto, K., Narayama, S., Katsuo, A., Shigematsu, I., and Yanase, H. (2002) Biosynthesis of p-anisaldehyde by the white-rot basidiomycete Pleurotus ostreatus. Journal of Bioscience and Bioengineering 93: 207–210.

Okosun, O.O., Yusuf, A.A., Crewe, R.M., and Pirk, C.W.W. (2015) Effects of age and Reproductive Status on Tergal Gland Secretions in Queenless Honey bee Workers, Apis mellifera scutellata and A. m. capensis. J Chem Ecol 41: 896–903.

Okuyama, E., Hasegawa, T., Matsushita, T., Fujimoto, H., Ishibashi, M., and Yamazaki, M. (2001) Analgesic Components of Saposhnikovia Root (Saposhnikovia divaricata). Chem Pharm Bull 49: 154–160.

Oliveira, A., Simões, R., Lima, C., Silva, F., Nunomura, S., Roque, R., et al. (2022) Essential oil of Piper purusanum C.DC (Piperaceae) and its main sesquiterpenes: biodefensives against malaria and dengue vectors, without lethal effect on non-target aquatic fauna. Environ Sci Pollut Res 29: 47242–47253.

Omata, A., Yomogida, K., Nakamura, S., Ohta, T., Izawa, Y., and Watanabe, S. (1991) The Scent of Lotus Flowers. Journal of Essential Oil Research 3: 221–227.

Orabi, K.Y., Mossa, J.S., and El-Feraly, F.S. (1991) Isolation and Characterization of Two Antimicrobial Agents from Mace (Myristica fragrans). J Nat Prod 54: 856–859.

Oster, M., Beck, J.J., Furrow, R.E., Yeung, K., and Field, C.B. (2015) In-field yellow starthistle (Centaurea solstitialis) volatile composition under elevated temperature and CO2 and implications for future control. Chemoecology 25: 313–323.

Otto, C., Yovkova, V., and Barth, G. (2011) Overproduction and secretion of α-ketoglutaric acid by microorganisms. Appl Microbiol Biotechnol 92: 689–695.

Padalia, R.C., Verma, R.S., Chauhan, A., and Chanotiya, C.S. (2012) Chemical composition of leaf and root essential oils of Boenninghausenia albiflora Reichb. from northern India. Natural Product Research 26: 2040–2044.

Padmaja, P.G., Woodcock, C.M., and Bruce, T.J.A. (2010) Electrophysiological and Behavioral Responses of Sorghum Shoot Fly, Atherigona soccata, to Sorghum Volatiles. J Chem Ecol 36: 1346–1353.

Pal, E., Allison, J., Guignard, Q., Hurley, B.P., Slippers, B., and Fourie, G. (2022) Characterisation of the Alarm Pheromone of Bathycoelia Distincta (Pentatomidae). J Chem Ecol 48: 791–801.

Paolini, J., Falchi, A., Quilichini, Y., Desjobert, J.-M., Cian, M.-C.D., Varesi, L., and Costa, J. (2009) Morphological, chemical and genetic differentiation of two subspecies of Cistus creticus L. (C. creticus subsp. eriocephalus and C. creticus subsp. corsicus). Phytochemistry 70: 1146–1160.

Papa, A.J. (2011) Propanols. In Ullmann’s Encyclopedia of Industrial Chemistry. Wiley-VCH Verlag GmbH & Co. KGaA (ed). Weinheim, Germany: Wiley-VCH Verlag GmbH & Co. KGaA, p. a22_173.pub2.

Park, J.-H., Lee, H.-S., and Chung, N. (2021) Acaricidal and repellent activities of Litsea cubeba (Lour.) oil and 3,7-dimethyl-2,6-octadienal against Haemaphysalis longicornis (Acari: Ixodidae). Appl Biol Chem 64: 88.

Parween, T., Bhandari, P., Jan, S., Mahmooduzzafar, Fatma, T., and Raza, S.K. (2017) Role of Bioinoculants as Plant Growth-Promoting Microbes for Sustainable Agriculture. In Agriculturally Important Microbes for Sustainable Agriculture. Meena, V.S., Mishra, P.K., Bisht, J.K., and Pattanayak, A. (eds). Singapore: Springer Singapore, pp. 183–206.

Patel, T.R., Hameed, N., and Martin, A.M. (1990) Initial steps of phloroglucinol metabolism in Penicillium simplicissimum. Arch Microbiol 153: 438–443.

Patrignani, F., Iucci, L., Belletti, N., Gardini, F., Guerzoni, M.E., and Lanciotti, R. (2008) Effects of sub-lethal concentrations of hexanal and 2-(E)-hexenal on membrane fatty acid composition and volatile compounds of Listeria monocytogenes, Staphylococcus aureus, Salmonella enteritidis and Escherichia coli. International Journal of Food Microbiology 123: 1–8.

Pauli, A. (2001) Antimicrobial properties of essential oil constituents. International Journal of Aromatherapy 11: 126–133.

Pavithra, P.S., Sreevidya, N., and Verma, R.S. (2009) Antibacterial activity and chemical composition of essential oil of Pamburus missionis. Journal of Ethnopharmacology 124: 151–153.

Peluffo, L., Lia, V., Troglia, C., Maringolo, C., Norma, P., Escande, A., et al. (2010) Metabolic profiles of sunflower genotypes with contrasting response to Sclerotinia sclerotiorum infection. Phytochemistry 71: 70–80.

Peng, W., Han, T., Xin, W.-B., Zhang, X.-G., Zhang, Q.-Y., Jia, M., and Qin, L.-P. (2011) Comparative research of chemical constituents and bioactivities between petroleum ether extracts of the aerial part and the rhizome of Atractylodes macrocephala. Med Chem Res 20: 146–151.

Pérez, L.P., Montesinos, Y.P., Olmedo, J.G., Rodriguez, R.B., Sánchez, R.R., Montenegro, O.N., et al. (2016) Effect of phloroglucinol on rooting and in vitro acclimatization of papaya (Carica papaya L. var. Maradol Roja). In Vitro CellDevBiol-Plant 52: 196–203.

Perveen, K., Bukhari, N.A., Al Masoudi, L.M., Alqahtani, A.N., Alruways, M.W., and Alkhattaf, F.S. (2022) Antifungal potential, chemical composition of Chlorella vulgaris and SEM analysis of morphological changes in Fusarium oxysporum. Saudi Journal of Biological Sciences 29: 2501–2505.

Peschel, S., Franke, R., Schreiber, L., and Knoche, M. (2007) Composition of the cuticle of developing sweet cherry fruit. Phytochemistry 68: 1017–1025.

Peterson, J.K., Horvat, R.J., and Elsey, K.D. (1994) Squash leaf glandular trichome volatiles: Identification and influence on behavior of female pickleworm moth [Diaphania nitidalis (Stoll.)] (Lepidoptera: Pyralidae). J Chem Ecol 20: 2099–2109.

Pettersen, E.O., Nome, O., Rønning, Ø.W., and Oftebro, R. (1983) Effects of benzaldehyde on survival and cell-cycle kinetics of human cells cultivated in vitro. European Journal of Cancer and Clinical Oncology 19: 507–514.

Pickering, G.J., Spink, M., Kotseridis, Y., Brindle, I.D., Sears, M., and Inglis, D. (2015) The influence of Harmonia axyridis morbidity on 2-Isopropyl-3-methoxypyrazine in “Cabernet Sauvignon” wine. VITIS - Journal of Grapevine Research 227 Pages.

Pino, J., Almora, K., and Marbot, R. (2003) Volatile components of papaya (Carica papaya L., Maradol variety) fruit. Flavour Fragr J 18: 492–496.

Pino, J.A., Mesa, J., Muñoz, Y., Martí, M.P., and Marbot, R. (2005) Volatile Components from Mango (Mangifera indica L.) Cultivars. J Agric Food Chem 53: 2213–2223.

Pistelli, L., Bertoli, A., Bilia, A.R., and Morelli, I. (1996) Minor constituents from Bupleurum fruticosum roots. Phytochemistry 41: 1579–1582.

Podstolski, A., Havkin-Frenkel, D., Malinowski, J., Blount, J.W., Kourteva, G., and Dixon, R.A. (2002) Unusual 4-hydroxybenzaldehyde synthase activity from tissue cultures of the vanilla orchid Vanilla planifolia. Phytochemistry 61: 611–620.

Potvin, J. and Péringer, P. (1993) Influence of n-propanol on growth and antibiotic production by an industrial strain of Streptomyces erythreus under different nutritional conditions. Biotechnol Lett 15: 455–460.

Pourhajibagher, M., Hosseini, N., and Bahador, A. (2023) Antimicrobial activity of D-amino acid in combination with photo-sonoactivated hypericin nanoparticles against Acinetobacter baumannii. BMC Microbiol 23: 23.

Pradhan, S., Nautiyal, V., and Dubey, R.C. (2022) Antioxidant potential and molecular docking of bioactive compound of Camellia sinensis and Camellia assamica with cytochrome P450. Arch Microbiol 204: 350.

Priyadarshi, S., Khanum, H., Ravi, R., Borse, B.B., and Naidu, M.M. (2016) Flavour characterisation and free radical scavenging activity of coriander (Coriandrum sativum L.) foliage. J Food Sci Technol 53: 1670–1678.

Proestos, C., Chorianopoulos, N., Nychas, G.-J.E., and Komaitis, M. (2005) RP-HPLC Analysis of the Phenolic Compounds of Plant Extracts. Investigation of Their Antioxidant Capacity and Antimicrobial Activity. J Agric Food Chem 53: 1190–1195.

Qi, P.-F., Johnston, A., Balcerzak, M., Rocheleau, H., Harris, L.J., Long, X.-Y., et al. (2012) Effect of salicylic acid on Fusarium graminearum, the major causal agent of fusarium head blight in wheat. Fungal Biology 116: 413–426.

Qiming, X., Haidong, C., Huixian, Z., and Daqiang, Y. (2006) Chemical composition of essential oils of two submerged macrophytes, Ceratophyllum demersum L. and Vallisneria spiralis L. Flavour Fragr J 21: 524–526.

Qiu, R., Qu, D., Trengove, R., Agarwal, M., Hardy, G.E.St.J., and Ren, Y. (2014) Headspace Solid-Phase Microextraction and Gas Chromatography-Mass Spectrometry for Analysis of VOCs Produced by Phytophthora cinnamomi. Plant Disease 98: 1099–1105.

Rajniak, J., Giehl, R.F.H., Chang, E., Murgia, I., von Wirén, N., and Sattely, E.S. (2018) Biosynthesis of redox-active metabolites in response to iron deficiency in plants. Nat Chem Biol 14: 442–450.

Ramabulana, A.-T., Steenkamp, P.A., Madala, N.E., and Dubery, I.A. (2020) Profiling of Altered Metabolomic States in Bidens pilosa Leaves in Response to Treatment by Methyl Jasmonate and Methyl Salicylate. Plants 9: 1275.

Ramaroson-Raonizafinimanana, B., Gaydou, É.M., and Bombarda, I. (1997) Hydrocarbons from Three Vanilla Bean Species: V. fragrans , V. madagascariensis , and V. tahitensis. J Agric Food Chem 45: 2542–2545.

Rees, C.A., Burklund, A., Stefanuto, P.-H., Schwartzman, J.D., and Hill, J.E. (2018) Comprehensive volatile metabolic fingerprinting of bacterial and fungal pathogen groups. J Breath Res 12: 026001.

Reinecke, M.G. and Zhao, Y.-Y. (1988) Phytochemical Studies of the Chinese Herb Tai-zi-shen, Pseudostellaria heterophylla. J Nat Prod 51: 1236–1240.

Rekoslavskaya, N.I. (1986) Possible role of N-malonyl-D-tryptophan as an auxin precursor. Biol Plant 28: 62–67.

Reveglia, P., Pacetti, A., Masi, M., Cimmino, A., Carella, G., Marchi, G., et al. (2021) Phytotoxic metabolites produced by Diaporthe eres involved in cane blight of grapevine in Italy. Natural Product Research 35: 2872–2880.

Reyes-Soto, C.Y., Villaseca-Flores, M., Ovalle-Noguez, E.A., Nava-Osorio, J., Galván-Arzate, S., Rangel-López, E., et al. (2022) Oleamide Reduces Mitochondrial Dysfunction and Toxicity in Rat Cortical Slices Through the Combined Action of Cannabinoid Receptors Activation and Induction of Antioxidant Activity. Neurotox Res 40: 2167–2178.

Rivero-Cruz, J.F. (2008) Antimicrobial compounds isolated from Haematoxylon brasiletto. Journal of Ethnopharmacology 119: 99–103.

Rohloff, J. (2002) Volatiles from rhizomes of Rhodiola rosea L. Phytochemistry 59: 655–661.

Romero-Olivares, A.L., Davie-Martin, C.L., Kramshøj, M., Rinnan, R., and Frey, S.D. (2022) Soil volatile organic compound emissions in response to soil warming and nitrogen deposition. Elementa: Science of the Anthropocene 10: 00065.

Rowen, E., Gutensohn, M., Dudareva, N., and Kaplan, I. (2017) Carnivore Attractant or Plant Elicitor? Multifunctional Roles of Methyl Salicylate Lures in Tomato Defense. J Chem Ecol 43: 573–585.

Rühl, V., Lotz-Winter, H., Neuss, A., Piepenbring, M., Zorn, H., and Rühl, M. (2018) Comprehensive analysis of the volatilome of Scytinostroma portentosum. Mycol Progress 17: 417–424.

Runkel, M., Moeller, A., Tegtmeier, M., Willigmann, I., and Legrum, W. (1997) Detection of scopolin in the grapefruit (Citrus paradisi M acf). Fresenius’ Journal of Analytical Chemistry 359: 516–520.

Sadraei, H., Ghanadian, M., Asghari, G., and Madadi, E. (2014) Antispasmodic activity of isovanillin and isoacetovanillon in comparison with Pycnocycla spinosa Decne.exBoiss extract on rat ileum. Res Pharm Sci 9: 187–192.

Sailler, B. and Glombitza, K.-W. (1999) Phlorethols and fucophlorethols from the brown alga Cystophora retroflexa. Phytochemistry 50: 869–881.

Sankawa, U. (1999) 1.01 - Overview. In Comprehensive Natural Products Chemistry. Elsevier, pp. 1–22.

Sarkar, D. and S. Naik, P. (2000) Phloroglucinol enhances growth and rate of axillary shoot proliferation in potato shoot tip cultures in vitro. Plant Cell, Tissue and Organ Culture 60: 139–149.

Sarmad, M., Mahalakshmipriya, A., and Senthil, K. (2012) Chemical Composition and In-vitro Antimicrobial Activity of Barleria lupulina Essential Oil. Journal of Herbs, Spices & Medicinal Plants 18: 101–109.

Saroglou, V., Marin, P.D., Rancic, A., Veljic, M., and Skaltsa, H. (2007) Composition and antimicrobial activity of the essential oil of six Hypericum species from Serbia. Biochemical Systematics and Ecology 35: 146–152.

Sashidhara, K.V., Verma, R.S., and Ram, P. (2006) Essential oil composition of Matricaria recutita L. from the lower region of the Himalayas. Flavour Fragr J 21: 274–276.

Schepetkin, I.A., Kushnarenko, S.V., Özek, G., Kirpotina, L.N., Utegenova, G.A., Kotukhov, Y.A., et al. (2015) Inhibition of Human Neutrophil Responses by the Essential Oil of Artemisia kotuchovii and Its Constituents. J Agric Food Chem 63: 4999–5007.

Schindler, M., Solar, S., and Sontag, G. (2005) Phenolic compounds in tomatoes. Natural variations and effect of gamma-irradiation. Eur Food Res Technol 221: 439–445.

Semmelroch, P. and Grosch, W. (1996) Studies on Character Impact Odorants of Coffee Brews. J Agric Food Chem 44: 537–543.

Seo, H.-Y., Yang, S.-H., Shim, S.-L., No, K.-M., Park, K.-S., Song, K.-D., and Kim, K.-S. (2007) Volatile organic compounds of Angelica gigas Nakai, Korean medicinal herb. Natural Product Research 21: 265–273.

Seo, W.H. and Baek, H.H. (2009) Characteristic Aroma-Active Compounds of Korean Perilla (Perilla frutescens Britton) Leaf. J Agric Food Chem 57: 11537–11542.

Servili, M. (2000) Relationships between the volatile compounds evaluated by solid phase microextraction and the thermal treatment of tomato juice: optimization of the blanching parameters. Food Chemistry 71: 407–415.

Shao, F., Zhang, L., Guo, J., Liu, X., Ma, W., Wilson, I.W., and Qiu, D. (2019) A comparative metabolomics analysis of the components of heartwood and sapwood in Taxus chinensis (Pilger) Rehd. Sci Rep 9: 17647.

Shao, J., He, Y., Li, F., Zhang, H., Chen, A., Luo, S., and Gu, J.-D. (2016) Growth inhibition and possible mechanism of oleamide against the toxin-producing cyanobacterium Microcystis aeruginosa NIES-843. Ecotoxicology 25: 225–233.

Sharma, P., Singh, V., and Ali, M. (2016) Chemical Composition and Antimicrobial Activity of Fresh Rhizome Essential Oil of Zingiber Officinale Roscoe. Phcogj 8: 185–190.

Sharopov, F., Valiev, A., Satyal, P., Gulmurodov, I., Yusufi, S., Setzer, W., and Wink, M. (2017) Cytotoxicity of the Essential Oil of Fennel (Foeniculum vulgare) from Tajikistan. Foods 6: 73.

Shettigar, M., Balotra, S., Cahill, D., Warden, A.C., Lacey, M.J., Kohler, H.-P.E., et al. (2018) Isolation of the (+)-Pinoresinol-Mineralizing Pseudomonas sp. Strain SG-MS2 and Elucidation of Its Catabolic Pathway. Appl Environ Microbiol 84: e02531-17.

Shilova, I.V. and Korotkova, E.I. (2017) Biologically Active Substances From Dropwort (Filipendula vulgaris) and Assessment of Their Antioxidant Properties. Pharm Chem J 51: 602–605.

Shin, B., Park, C., Imlay, J.A., and Park, W. (2018) 4-Hydroxybenzaldehyde sensitizes Acinetobacter baumannii to amphenicols. Appl Microbiol Biotechnol 102: 2323–2335.

Shiromi, P.S.A.I., Hewawasam, R.P., Jayalal, R.G.U., Rathnayake, H., Wijayaratne, W.M.D.G.B., and Wanniarachchi, D. (2021) Chemical Composition and Antimicrobial Activity of Two Sri Lankan Lichens, Parmotrema rampoddense, and Parmotrema tinctorum against Methicillin-Sensitive and Methicillin-Resistant Staphylococcus aureus. Evidence-Based Complementary and Alternative Medicine 2021: 1–18.

Showler, A.T. and Harlien, J.L. (2018) Effects of the Botanical Compound p-Anisaldehyde on Horn Fly (Diptera: Muscidae) Repellency, Mortality, and Reproduction. Journal of Medical Entomology 55: 183–192.

Silva, E., Matsuo, A., Figueiredo, C., Chaves, M., Sartorelli, P., and Lago, J. (2013) Chemical constituents and cytotoxic evaluation of essential oils from leaves of Porcelia macrocarpa ( Annonaceae ). Nat Prod Commun 8: 277–279.

Silva, M., Luz, A., Zoghbi, M., Ramos, L., and Maia, J. (1984) Essential oils of some amazonian Mikania species. Phytochemistry 23: 2374–2376.

Silva, W.P.K., Deraniyagala, S.A., Wijesundera, R.L.C., Karunanayake, E.H., and Priyanka, U.M.S. (2002) Isolation of scopoletin from leaves of Hevea brasiliensis and the effect of scopoletin on pathogens of H. brasiliensis. Mycopathologia 153: 199–202.

Simão, A.A., Marques, T.R., Marcussi, S., and Corrêa, A.D. (2017) Aqueous extract of Psidium guajava leaves: phenolic compounds and inhibitory potential on digestive enzymes. An Acad Bras Ciênc 89: 2155–2165.

Simmonds, J. and Robinson, G.K. (1998) Formation of benzaldehyde by Pseudomonas putida ATCC 12633. Applied Microbiology and Biotechnology 50: 353–358.

Šimonovičová, A., Kupka, D., Nosalj, S., Kraková, L., Drahovská, H., Bártová, Z., et al. (2020) Differences in metabolites production using the Biolog FF Microplate^TM^ system with an emphasis on some organic acids of Aspergillus niger wild type strains. Biologia 75: 1537–1546.

Singab, R.A., Elleboudy, N.S., Elkhatib, W.F., Yassein, M.A., and Hassouna, N.A. (2022) Improvement of caffeic acid biotransformation into para‐hydroxybenzoic acid by Candida albicans CI‐24 via gamma irradiation and model‐based optimization. Biotech and App Biochem 69: 469–478.

Singh, H.P., Kaur, S., Mittal, S., Batish, D.R., and Kohli, R.K. (2009) Essential Oil of Artemisia scoparia Inhibits Plant Growth by Generating Reactive Oxygen Species and Causing Oxidative Damage. J Chem Ecol 35: 154–162.

Sini, K., Flouri, F., and Balis, C. (1995) Influence of Asparagine and Aspartic Acid on Growth of Azospirillum. In Azospirillum VI and Related Microorganisms. Fendrik, I., del Gallo, M., Vanderleyden, J., and de Zamaroczy, M. (eds). Berlin, Heidelberg: Springer Berlin Heidelberg, pp. 331–334.

Sipailiene, A., Venskutonis, P.R., Sarkinas, A., and Cypiene, V. (2005) Composition and antimicrobial activity of celery (Apium Graveolens) leaf and root extracts obtained with liquid carbon dioxide. Acta Hortic 71–77.

Sircar, D., Dey, G., and Mitra, A. (2007) A Validated HPLC Method for Simultaneous Determination of 2-Hydroxy-4-methoxybenzaldehyde and 2-Hydroxy-4-methoxybenzoic Acid in Root Organs of Hemidesmus indicus. Chroma 65: 349–353.

Sircar, D. and Mitra, A. (2008) Evidence for p-hydroxybenzoate formation involving enzymatic phenylpropanoid side-chain cleavage in hairy roots of Daucus carota. Journal of Plant Physiology 165: 407–414.

Sircar, D., Mukherjee, C., Beuerle, T., Beerhues, L., and Mitra, A. (2011) Characterization of p-hydroxybenzaldehyde dehydrogenase, the final enzyme of p-hydroxybenzoic acid biosynthesis in hairy roots of Daucus carota. Acta Physiol Plant 33: 2019–2024.

Siri-udom, S., Suwannarach, N., and Lumyong, S. (2016) Existence of Muscodor vitigenus, M. equiseti and M. heveae sp. nov. in leaves of the rubber tree (Hevea brasiliensis Müll.Arg.), and their biocontrol potential. Ann Microbiol 66: 437–448.

Siwinska, J., Siatkowska, K., Olry, A., Grosjean, J., Hehn, A., Bourgaud, F., et al. (2018) Scopoletin 8-hydroxylase: a novel enzyme involved in coumarin biosynthesis and iron-deficiency responses in Arabidopsis. Journal of Experimental Botany 69: 1735–1748.

Sonboli, A., Salehi, P., and Nejad Ebrahimi, S. (2005) Essential Oil Composition and Antibacterial Activity of the Leaves of Stachys schtschegleevii from Iran. Chem Nat Compd 41: 171–174.

Song, J.E. and Lee, H.S. (2018) Mite color alteration and acaricidal activity of 3,7-dimethyl-2,6-octadienal and its structural analogues against the stored food pest mite Tyrophagus putrescentiae. Exp Appl Acarol 76: 355–363.

Sotiroudis, G., Melliou, E., Sotiroudis, T.G., and Chinou, I. (2010) Chemical analysis, antioxidant and antimicrobial activity of three greek cucumber (Cucumis sativus) cultivars. Journal of Food Biochemistry 34: 61–78.

Srikanta, B.M., Harish Nayaka, M.A., and Dharmesh, S.M. (2011) Inhibition of Helicobacter pylori growth and its cytotoxicity by 2-hydroxy 4-methoxy benzaldehyde of Decalepis hamiltonii (Wight & Arn); a new functional attribute. Biochimie 93: 678–688.

Stashenko, E.E., Martínez, J.R., Cárdenas-Vargas, S., Saavedra-Barrera, R., and Durán, D.C. (2013) GC-MS study of compounds isolated from Coffea arabica flowers by different extraction techniques: Sample preparation. J Sep Science 36: 2901–2914.

Stephen, J., Shabanamol, S., Rishad, K.S., and Jisha, M.S. (2015) Growth enhancement of rice (Oryza sativa) by phosphate solubilizing Gluconacetobacter sp. (MTCC 8368) and Burkholderia sp. (MTCC 8369) under greenhouse conditions. 3 Biotech 5: 831–837.

Sukatar, A., Karabay-Yavaşsoglu, N.U., Ozdemir, G., and Horzum, Z. (2006) Antimicrobial activity of volatile component and various extracts of Enteromorpha linza (Linnaeus) J. Agardh from the coast of Izmir, Turkey. Ann Microbiol 56: 275–279.

Sun, M., Fu, D., Teng, Y., Shen, Y., Luo, Y., Li, Z., and Christie, P. (2011) In situ phytoremediation of PAH-contaminated soil by intercropping alfalfa (Medicago sativa L.) with tall fescue (Festuca arundinacea Schreb.) and associated soil microbial activity. J Soils Sediments 11: 980–989.

Sun, N.-J., Ho, D.K., Hu, X.E., Sneddon, J.M., Stephens, R.E., and Cassady, J.M. (1995) New Cytotoxic Fatty Acid from Desmos cochinchinensis (Annonaceae). Natural Product Letters 7: 35–41.

Sun, Y., Ren, G., Shi, Q., Zhu, H., Zhou, N., Kong, X., et al. (2023) Identification of a Novel Coumarins Biosynthetic Pathway in the Endophytic Fungus Fusarium oxysporum GU-7 with Antioxidant Activity. Appl Environ Microbiol 89: e01601-22.

Sung, C., Jung, E., Choi, K.-Y., Bae, J., Kim, M., Kim, J., et al. (2015) The production of ω-hydroxy palmitic acid using fatty acid metabolism and cofactor optimization in Escherichia coli. Appl Microbiol Biotechnol 99: 6667–6676.

Surwase, S.N. and Jadhav, J.P. (2011) Bioconversion of l-tyrosine to l-DOPA by a novel bacterium Bacillus sp. JPJ. Amino Acids 41: 495–506.

Tabanca, N., Demirci, F., Ozek, T., Tumen, G., and Baser, K.H.C. (2001) Composition and Antimicrobial Activity of the Essential Oil of Origanum × dolichosiphon P. H. Davis. Chemistry of Natural Compounds 37: 238–241.

Tabanca, N., Gao, Z., Demirci, B., Techen, N., Wedge, D.E., Ali, A., et al. (2014) Molecular and Phytochemical Investigation of Angelica dahurica and Angelica pubescentis Essential Oils and Their Biological Activity against Aedes aegypti , Stephanitis pyrioides and Colletotrichum Species. J Agric Food Chem 62: 8848–8857.

Taira, J., Toyoshima, R., Ameku, N., Iguchi, A., and Tamaki, Y. (2018) Vanillin production by biotransformation of phenolic compounds in fungus, Aspergillus luchuensis. AMB Expr 8: 40.

Takeuchi, S., Kochi, M., Sakaguchi, K., Nakagawa, K., and Mizutani, T. (1978) Benzaldehyde as a Carcinostatic Principle in Figs. Agricultural and Biological Chemistry 42: 1449–1451.

Talapatra, B., Bhaumik, A., and Talapatra, S.K. (2010) ChemInform Abstract: 2-Hydroxy-1,2,3-propanetricarboxylic Acid 2-Methyl Ester, a New Natural Product from Rhus parviflora: A Simple Achiral Molecule Having Both Enantiotopic and Diastereotopic Hydrogens. ChemInform 25: no-no.

Tanaka, K. and Komatsu, K. (2007) Comparative study on volatile components of Nardostachys Rhizome. J Nat Med 62: 112–116.

Tang, X., Zhou, Y., Liu, Y., Chen, H., and Ge, H. (2022) Volatile compound metabolism during cherry tomato fruit development and ripening. Food Measure.

Taofiq, O., González-Paramás, A.M., Barreiro, M.F., and Ferreira, I.C.F.R. (2017) Hydroxycinnamic Acids and Their Derivatives: Cosmeceutical Significance, Challenges and Future Perspectives, a Review. Molecules 22: 281.

Tava, A. and Pecetti, L. (1997) Volatiles from Medicago sativa complex flowers. Phytochemistry 45: 1145–1148.

Thanomsub, B., Watcharachaipong, T., Chotelersak, K., Arunrattiyakorn, P., Nitoda, T., and Kanzaki, H. (2004) Monoacylglycerols: glycolipid biosurfactants produced by a thermotolerant yeast, Candida ishiwadae. J Appl Microbiol 96: 588–592.

Thöming, G., Koczor, S., Szentkirályi, F., Norli, H.R., Tasin, M., and Knudsen, G.K. (2020) Attraction of Chrysotropia ciliata (Neuroptera, Chrysopidae) Males to P-Anisaldehyde, a Compound with Presumed Pheromone Function. J Chem Ecol 46: 597–609.

Thompson, M.L., Marriott, R., Dowle, A., and Grogan, G. (2010) Biotransformation of β-myrcene to geraniol by a strain of Rhodococcus erythropolis isolated by selective enrichment from hop plants. Appl Microbiol Biotechnol 85: 721–730.

Thuong, P.T., Hung, T.M., Ngoc, T.M., Ha, D.T., Min, B.S., Kwack, S.J., et al. (2010) Antioxidant activities of coumarins from Korean medicinal plants and their structure–activity relationships. Phytother Res 24: 101–106.

Tian, J., Zeng, X., Zhang, S., Wang, Y., Zhang, P., Lü, A., and Peng, X. (2014) Regional variation in components and antioxidant and antifungal activities of Perilla frutescens essential oils in China. Industrial Crops and Products 59: 69–79.

Tolstorozhev, G.B., Skornyakov, I.V., Belkov, M.V., Shadyro, O.I., Brinkevich, S.D., and Samovich, S.N. (2012) Hydrogen bonds and antiviral activity of benzaldehyde derivatives. J Appl Spectrosc 79: 645–650.

Tomás‐Gallardo, L., Gómez‐Álvarez, H., Santero, E., and Floriano, B. (2014) Combination of degradation pathways for naphthalene utilization in R hodococcus sp. strain TFB. Microbial Biotechnology 7: 100–113.

Tomas-Hernandez, S., Blanco, J., Garcia-Vallvé, S., Pujadas, G., Ojeda-Montes, M.J., Gimeno, A., et al. (2021) Anti-Inflammatory and Immunomodulatory Effects of the Grifola frondosa Natural Compound o-Orsellinaldehyde on LPS-Challenged Murine Primary Glial Cells. Roles of NF-κβ and MAPK. Pharmaceutics 13: 806.

Tomczykowa, M., Leszczyńska, K., Tomczyk, M., Tryniszewska, E., and Kalemba, D. (2011) Composition of the Essential Oil of Bidens tripartita L. Roots and Its Antibacterial and Antifungal Activities. Journal of Medicinal Food 14: 428–433.

Tonouchi, A. (2004) Anaerobic 2-Propanol Degradation in Anoxic Paddy Soil and the Possible Role of Methanogens in Its Degradation. Curr Microbiol 49:.

Toyoda, K., Yaoita, Y., and Kikuchi, M. (2006) Constituents of the leaves and roots of Ligularia stenocephala MATSUM. et KOIDZ. J Nat Med 60: 329–330.

Tunchai, M., Hida, A., Oku, S., Nakashimada, Y., Tajima, T., and Kato, J. (2017) Identification and characterization of chemosensors for d-malate, unnatural enantiomer of malate, in Ralstonia pseudosolanacearum. Microbiology 163: 233–242.

Turgumbayeva, A.A., Ustenova, G.O., Yeskalieva, B.K., Ramazanova, B.A., Rahimov, K.D., Aisa, H., and Juszkiewicz, K.T. (2018) Volatile oil composition of Carthamus Tinctorius L. flowers grown in Kazakhstan. Ann Agric Environ Med 25: 87–89.

Turner, C.E., Elsohly, M.A., and Boeren, E.G. (1980) Constituents of Cannabis sativa L. XVII. A Review of the Natural Constituents. J Nat Prod 43: 169–234.

Ulrich, K.R., Feldlaufer, M.F., Kramer, M., and St. Leger, R.J. (2015) Inhibition of the entomopathogenic fungus Metarhizium anisopliae sensu lato in vitro by the bed bug defensive secretions (E)-2-hexenal and (E)-2-octenal. BioControl 60: 517–526.

Urano, N., Fujii, M., Kaino, H., Matsubara, M., and Kataoka, M. (2015) Fermentative production of 1-propanol from sugars using wild-type and recombinant Shimwellia blattae. Appl Microbiol Biotechnol 99: 2001–2008.

Ursem, R., Tikunov, Y., Bovy, A., van Berloo, R., and van Eeuwijk, F. (2008) A correlation network approach to metabolic data analysis for tomato fruits. Euphytica 161: 181.

Ushio, K., Nakagawa, Kouzou, Nakagawa, Katsuhiko, and Watanabe, K. (1992) An easy access to optically pure (R)-malic acid via enantioselective hydrolysis of diethyl malate byRhizopus lipase. Biotechnol Lett 14: 795–800.

Vandendriessche, T., Keulemans, J., Geeraerd, A., Nicolai, B.M., and Hertog, M.L.A.T.M. (2012) Evaluation of fast volatile analysis for detection of Botrytis cinerea infections in strawberry. Food Microbiology 32: 406–414.

Vasilevskaya, N.A., Utkina, N.K., Maksimov, O.B., and Boguslavskaya, L.V. (1994) Phenolic antioxidants of the bark ofFraxinus mandshurica. Chem Nat Compd 30: 629–630.

Vaughn, S.F. and Gardner, H.W. (1993) Lipoxygenase-derived aldehydes inhibit fungi pathogenic on soybean. J Chem Ecol 19: 2337–2345.

Vélez-Bermúdez, I.C. and Schmidt, W. (2023) Plant strategies to mine iron from alkaline substrates. Plant Soil 483: 1–25.

Veneziani, R.C.S., Camilo, D., and Oliveira, R. de (1999) Constituents of Mikania glomerata Sprengel. Biochemical Systematics and Ecology 27: 99–102.

Vidotti, G.J., Zimmermann, A., Sarragiotto, M.H., Nakamura, C.V., and Dias Filho, B.P. (2006) Antimicrobial and phytochemical studies on Pedilanthus tithymaloides. Fitoterapia 77: 43–46.

Voegtle, H.L., Jones, T.H., Davidson, D.W., and Snelling, R.R. (2008) E-2-Ethylhexenal, E-2-Ethyl-2-Hexenol, Mellein, and 4-Hydroxymellein in Camponotus species from Brunei. J Chem Ecol 34: 215–219.

Vu, H.-N.T., Nguyen, D.T., Nguyen, H.Q., Chu, H.H., Chu, S.K., Chau, M.V., and Phi, Q.-T. (2018) Antimicrobial and Cytotoxic Properties of Bioactive Metabolites Produced by Streptomyces cavourensis YBQ59 Isolated from Cinnamomum cassia Prels in Yen Bai Province of Vietnam. Curr Microbiol 75: 1247–1255.

Wagner, H., Kreher, B., Lotter, H., Hamburger, M.O., and Cordell, G.A. (1989) Structure Determination of New Isomeric Naphtho[2,3-b]furan-4,9-diones from Tabebuia avellanedae by the selective-INEPT technique. Helv Chim Acta 72: 659–667.

Wahab, I.R.A., Blagojević, P.D., Radulović, N.S., and Boylan, F. (2011) Volatiles of Curcuma mangga Val. & Zijp (Zingiberaceae) from Malaysia. Chemistry & Biodiversity 8: 2005–2014.

Walker, J.R.L. and Taylor, B.G. (1983) Metabolism of phloroglucinol by Fusarium solani. Arch Microbiol 134: 123–126.

Wang, G., Fan, J., Wu, H., Zhang, X., Li, G., Zhang, H., et al. (2013) Nonhongiella spirulinensis gen. nov., sp. nov., a bacterium isolated from a cultivation pond of Spirulina platensis in Sanya, China. Antonie van Leeuwenhoek 104: 933–939.

Wang, J., Liu, H., Zhao, J., Gao, H., Zhou, L., Liu, Z., et al. (2010) Antimicrobial and Antioxidant Activities of the Root Bark Essential Oil of Periploca sepium and Its Main Component 2-Hydroxy-4-methoxybenzaldehyde. Molecules 15: 5807–5817.

Wang, J., Zhao, J., Liu, H., Zhou, L., Liu, Z., Wang, Jingguo, et al. (2010) Chemical Analysis and Biological Activity of the Essential Oils of Two Valerianaceous Species from China: Nardostachys chinensis and Valeriana officinalis. Molecules 15: 6411–6422.

Wang, L., Wang, Y., Wang, W., Zheng, F., and Chen, F. (2019) Comparison of volatile compositions of 15 different varieties of Chinese jujube (Ziziphus jujuba Mill.). J Food Sci Technol 56: 1631–1640.

Wang, L., Wang, Z., Li, X., Zhang, Huihui, Zhou, X., and Zhang, Hanqi (2010) Analysis of Volatile Compounds in the Pericarp of Zanthoxylum bungeanum Maxim. by Ultrasonic Nebulization Extraction Coupled with Headspace Single-Drop Microextraction and GC–MS. Chroma 71: 455–459.

Wang, P.-C., Ran, X.-H., Luo, H.-R., Ma, Q.-Y., Liu, Y.-Q., Zhou, J., and Zhao, Y.-X. (2013) Phenolic Compounds from the Roots of Valeriana officinalis var. latifolia. Journal of the Brazilian Chemical Society.

Wang, X., Luo, Y., Liu, D., Wang, J., Wei, S., and Zhao, L. (2018) Complete genome sequence of the Robinia pseudoacacia L. symbiont Mesorhizobium amorphae CCNWGS0123. Stand in Genomic Sci 13: 18.

Wei, Xiaopeng, Lu, W., Mao, L., Han, X., Wei, Xiaobo, Zhao, X., et al. (2020) ABF2 and MYB transcription factors regulate feruloyl transferase FHT involved in ABA-mediated wound suberization of kiwifruit. Journal of Experimental Botany 71: 305–317.

Wen, Y.-Q., He, F., Zhu, B.-Q., Lan, Y.-B., Pan, Q.-H., Li, C.-Y., et al. (2014) Free and glycosidically bound aroma compounds in cherry (Prunus avium L.). Food Chemistry 152: 29–36.

Wesolowska, A., Grzeszczuk, M., and Kulpa, D. (2015) Propagation Method and Distillation Apparatus Type Affect Essential Oil from Different Parts of Matricaria recutita L. Plants. Journal of Essential Oil Bearing Plants 18: 179–194.

Whang, W.K., Park, H.S., Ham, I., Oh, M., Namkoong, H., Kim, H.K., et al. (2005) Natural compounds, fraxin and chemicals structurally related to fraxin protect cells from oxidative stress. Exp Mol Med 37: 436–446.

Williams, C.A. (2003) GALACTOSE. In Encyclopedia of Food Sciences and Nutrition. Elsevier, pp. 2843–2846.

Wilson, C.W., Shaw, P.E., Knight, R.J., Nagy, S., and Klim, M. (1985) Volatile constituents of carambola (Averrhoa carambola L.). J Agric Food Chem 33: 199–201.

Wisespongpand, P. and Kuniyoshi, M. (2003) Bioactive phloroglucinols from the brown alga Zonaria diesingiana. Journal of Applied Phycology 15: 225–228.

Witkowska-Banaszczak, E. and Długaszewska, J. (2017) Essential oils and hydrophilic extracts from the leaves and flowers of Succisa pratensis Moench. and their biological activity. Journal of Pharmacy and Pharmacology 69: 1531–1539.

Wu, P., Kuo, M.C., and Ho, C.T. (1990) Glycosidically bound aroma compounds in ginger (Zingiber officinale Roscoe). J Agric Food Chem 38: 1553–1555.

Xanthis, V., Fitsiou, E., Voulgaridou, G.-P., Bogadakis, A., Chlichlia, K., Galanis, A., and Pappa, A. (2021) Antioxidant and Cytoprotective Potential of the Essential Oil Pistacia lentiscus var. chia and Its Major Components Myrcene and α-Pinene. Antioxidants 10: 127.

Xiong, C., Li, Q., Li, S., Chen, C., Chen, Z., and Huang, W. (2017) In vitro Antimicrobial Activities and Mechanism of 1-Octen-3-ol against Food-related Bacteria and Pathogenic Fungi. J Oleo Sci 66: 1041–1049.

Xiong, Q., Sun, C., Shi, H., Cai, S., Xie, H., Liu, F., and Zhu, J. (2022) Analysis of Related Metabolites Affecting Taste Values in Rice under Different Nitrogen Fertilizer Amounts and Planting Densities. Foods 11: 1508.

Xu, F., Wang, Q., and Haji, A.A. (2011) Analysis of essential oil extracted from Lactuca sativa seeds growing in Xinjiang by GC-MS. Zhong Yao Cai 34: 1887–1891.

Xu, Y. and Barringer, S. (2010) Comparison of Tomatillo and Tomato Volatile Compounds in the Headspace by Selected Ion Flow Tube Mass Spectrometry (SIFT-MS). Journal of Food Science 75: C268–C273.

Xu, Z., Huang, S., Du, F., Peng, C., and Tian, Y. (2003) Studies on constituents of Rosa bracteata fruits (II). Zhong Yao Cai 26: 867–869.

Xue, Y., Liu, Y., Xie, Y., Cong, C., Wang, G., An, L., et al. (2020) Antioxidant activity and mechanism of dihydrochalcone C-glycosides: Effects of C-glycosylation and hydroxyl groups. Phytochemistry 179: 112393.

Yamaguchi, K. and Shibamoto, T. (1979) Volatile constituents of Castanopsis flower. J Agric Food Chem 27: 847–850.

Yang, F. and Cao, Y. (2012) Biosynthesis of phloroglucinol compounds in microorganisms—review. Appl Microbiol Biotechnol 93: 487–495.

Yang, J.-Y., Park, J.-H., Lee, M.-J., Lee, J.-H., and Lee, H.-S. (2017) Antimicrobial Effects of 7,8-Dihydroxy-6-Methoxycoumarin and 7-Hydroxy-6-Methoxycoumarin Analogues against Foodborne Pathogens and the Antimicrobial Mechanisms Associated with Membrane Permeability. Journal of Food Protection 80: 1784–1790.

Yang, Z.-G., Jia, L.-N., Shen, Y., Ohmura, A., and Kitanaka, S. (2011) Inhibitory Effects of Constituents from Euphorbia lunulata on Differentiation of 3T3-L1 Cells and Nitric Oxide Production in RAW264.7 Cells. Molecules 16: 8305–8318.

Yanishlieva, N.V. and Marinova, E.M. (1996) Antioxidative effectiveness of some natural antioxidants in sunflower oil. Z Lebensm Unters Forch 203: 220–223.

Yastrebova, O.V., Malysheva, A.A., and Plotnikova, E.G. (2022) Halotolerant Terephthalic Acid-Degrading Bacteria of the Genus Glutamicibacter. Appl Biochem Microbiol 58: 590–597.

Yi, X., Gu, H., Gao, Q., Liu, Z.L., and Bao, J. (2015) Transcriptome analysis of Zymomonas mobilis ZM4 reveals mechanisms of tolerance and detoxification of phenolic aldehyde inhibitors from lignocellulose pretreatment. Biotechnol Biofuels 8: 153.

Yin, G., Zeng, H., He, M., and Wang, M. (2009) Extraction of Teucrium manghuaense and Evaluation of the Bioactivity of Its Extract. IJMS 10: 4330–4341.

Youssef, F.S., Altyar, A.E., Omar, A.M., and Ashour, M.L. (2021) Phytoconstituents, In Vitro Anti-Infective Activity of Buddleja indica Lam., and In Silico Evaluation of its SARS-CoV-2 Inhibitory Potential. Front Pharmacol 12: 619373.

Yuan, G., Zhao, L., Du, Y., Yu, H., Shi, X., Chen, W., and Chen, G. (2022) Repellence or attraction: secondary metabolites in pepper mediate attraction and defense against Spodoptera litura. Pest Management Science 78: 4859–4870.

Yun, L.J. and Chen, W.L. (2011) SA and ROS are involved in methyl salicylate-induced programmed cell death in Arabidopsis thaliana. Plant Cell Rep 30: 1231–1239.

Zai-Chang, Y., Bo-Chu, W., Xiao-Sheng, Y., and Qiang, W. (2005) Chemical composition of the volatile oil from Cynanchum stauntonii and its activities of anti-influenza virus. Colloids and Surfaces B: Biointerfaces 43: 198–202.

Zakaria, I., Ahmat, N., Jaafar, F.M., and Widyawaruyanti, A. (2012) Flavonoids with antiplasmodial and cytotoxic activities of Macaranga triloba. Fitoterapia 83: 968–972.

Zamponi, L., Michelozzi, M., and Capretti, P. (2006) Effects of four monoterpenes on the growth in vitro of some Heterobasidion spp. and two Leptographium species. J Plant Dis Prot 113: 164–167.

Zeng, R.S. and Mallik, A.U. (2006) Selected Ectomycorrhizal Fungi of Black Spruce (Picea mariana) can Detoxify Phenolic Compounds of Kalmia angustifolia. J Chem Ecol 32: 1473–1489.

Zeringue, H.J. and McCormick, S.P. (1989) Relationships between cotton leaf-derived volatiles and growth of Aspergillus flavus. JAOCS 66: 581–585.

Zhang, D., Chen, Wenhao, Chen, Wenxing, Song, X., Han, C., Wang, Y., and Chen, G. (2013) Three New Ursane-Type Triterpenoids from the Stems of Saprosma merrillii. Molecules 18: 14496–14504.

Zhang, L.-S., Xu, P., Chu, M.-Y., Zong, M.-H., Yang, J.-G., and Lou, W.-Y. (2019) Using 1-propanol to significantly enhance the production of valuable odd-chain fatty acids by Rhodococcus opacus PD630. World J Microbiol Biotechnol 35: 164.

Zhang, M.-Y., Xie, J., Zhang, T.-Y., Xu, H., Cheng, J., Li, S.-H., et al. (2014) Sinomonas notoginsengisoli sp. nov., isolated from the rhizosphere of Panax notoginseng. Antonie van Leeuwenhoek 106: 827–835.

Zhang, Y. and Ezeji, T.C. (2014) Elucidating and alleviating impacts of lignocellulose-derived microbial inhibitors on Clostridium beijerinckii during fermentation of Miscanthus giganteus to butanol. Journal of Industrial Microbiology and Biotechnology 41: 1505–1516.

Zhang, Y., Jiao, R., Lu, Y., and Yao, L. (2016) Improvement of chaetominine production by tryptophan feeding and medium optimization in submerged fermentation of Aspergillus fumigatus CY018. Bioresour Bioprocess 3: 45.

Zhao, X., Shen, J., Chang, K.J., and Kim, S.H. (2013) Analysis of Fatty Acids and Phytosterols in Ethanol Extracts of Nelumbo nucifera Seeds and Rhizomes by GC-MS. J Agric Food Chem 61: 6841–6847.

Zhao, Y., Wang, X., Wang, Z., Lu, Y., Fu, C., and Chen, S. (2006) Essential oil of Actinidia macrosperma, a catnip response kiwi endemic to China. J Zhejiang Univ - Sci B 7: 708–712.

Zhao, Y., Zhan, P., Tian, H.-L., Wang, P., Lu, C., Tian, P., and Zhang, Y.-Y. (2021) Insights into the Aroma Profile in Three Kiwifruit Varieties by HS-SPME-GC-MS and GC-IMS Coupled with DSA. Food Anal Methods 14: 1033–1042.

Zheng, G., jia, Y., Zhao, X., Zhang, F., Luo, S., Li, S., and Li, W. (2012) o-Coumaric acid from invasive Eupatorium adenophorum is a potent phytotoxin. Chemoecology 22: 131–138.

Zhiqun, T., Jian, Z., Junli, Y., Chunzi, W., and Danju, Z. (2017) Allelopathic effects of volatile organic compounds from Eucalyptus grandis rhizosphere soil on Eisenia fetida assessed using avoidance bioassays, enzyme activity, and comet assays. Chemosphere 173: 307–317.

Zhou, H., Xu, Z., Cai, C., Li, J., and Jin, M. (2022) Deciphering the metabolic distribution of vanillin in Rhodococcus opacus during lignin valorization. Bioresource Technology 347: 126348.

Zhou, N.Y., Fuenmayor, S.L., and Williams, P.A. (2001) nag genes of Ralstonia (formerly Pseudomonas) sp. strain U2 encoding enzymes for gentisate catabolism. J Bacteriol 183: 700–708.

Zhou, Y.-B., Ye, R.-R., Lu, X.-F., Lin, P.-C., Yang, S.-B., Yue, P.-P., et al. (2009) GC–MS analysis of liposoluble constituents from the stems of Cynomorium songaricum. Journal of Pharmaceutical and Biomedical Analysis 49: 1097–1100.

Zhu, L. and Tian, Y. (2013) Chemical composition and larvicidal activity of essential oil of Artemisia gilvescens against Anopheles anthropophagus. Parasitol Res 112: 1137–1142.

Zou, C., Li, Z., and Yu, D. (2010) Bacillus megaterium strain XTBG34 promotes plant growth by producing 2-pentylfuran. J Microbiol 48: 460–466.

Zou, Y., Qian, Z.-J., Li, Y., Kim, M.-M., Lee, S.-H., and Kim, S.-K. (2008) Antioxidant Effects of Phlorotannins Isolated from Ishige okamurae in Free Radical Mediated Oxidative Systems. J Agric Food Chem 56: 7001–7009.
